# Feasibility of a drug allergy registry-based excipient allergy database and call for universal mandatory drug ingredient disclosure: the case of PEG

**DOI:** 10.3389/falgy.2023.1331036

**Published:** 2024-01-16

**Authors:** Andy Ka Chun Kan, Valerie Chiang, Chinmoy Saha, Elaine Y. L. Au, Philip Hei Li

**Affiliations:** ^1^ Division of Rheumatology and Clinical Immunology, Department of Medicine, Queen Mary Hospital, The University of Hong Kong, Hong Kong, Hong Kong SAR, China; ^2^ Division of Clinical Immunology, Department of Pathology, Queen Mary Hospital, Hong Kong, Hong Kong SAR, China

**Keywords:** excipient, polyethylene glycol, drug allergy, big data, registry

## Abstract

**Background:**

Excipient allergy is a rare, but potentially lethal, form of drug allergy. Diagnosing excipient allergy remains difficult in regions without mandatory drug ingredient disclosure and is a significant barrier to drug safety.

**Objective:**

To investigate the feasibility of a drug allergy registry-based excipient database to identify potential excipient culprits in patients with history of drug allergy, using polyethylene glycol (PEG) as an example.

**Methods:**

An excipient registry was created by compiling the excipient lists pertaining to all available formulations of the top 50 most reported drug allergy culprits in Hong Kong. Availability of excipient information, and its relationship with total number of formulations of individual drugs were analysed. All formulations were checked for the presence or absence of PEG.

**Results:**

Complete excipient information was available for 36.5% (729/2,000) of all formulations of the top 50 reported drug allergy culprits in Hong Kong. The number of formulations for each drug was associated with proportion of available excipient information (*ρ* = 0.466, *p* = 0.001). Out of 729 formulations, 109 (15.0%) and 620 (85.0%) were confirmed to contain and not contain PEG, respectively. Excipient information was not available for the other 1,271 (63.6%) formulations. We were unable to confirm the presence or absence of PEG in any of the top 50 drug allergy culprits in Hong Kong.

**Conclusion:**

In countries without mandatory drug ingredient disclosure, excipient databases are unlikely able to identify potential excipient allergy in drug allergy patients. Legislations to enforce mandatory and universal ingredient disclosure are urgently needed.

## 1 Introduction

Excipient allergy is an uncommon form of drug allergy which can pose as a significant diagnostic challenge. Excipients often present as hidden allergens within certain drug or vaccine formulations, which can be incorrectly mistaken for factitious or multiple drug allergies (1–3). Among oral medications, over 90% of formulations contain at least one potential allergenic substance (4). Missed or misdiagnosis of excipient allergy can be potentially lethal and lead to inappropriate drug avoidance. Polyethylene glycol (PEG) is one example of the several clinically significant and common excipients associated with life-threatening anaphylaxis (5). Although an uncommon entity, PEG and other excipient allergies can almost never be confirmed or excluded in countries without pharmaceutical legislations mandating complete drug ingredient disclosure. This devastating impact was exemplified during global COVID-19 vaccination campaigns—with superfluous fear of “potential” excipient allergy among patients with history of drug allergy, which greatly fuelled vaccine hesitancy and impeded uptake (6–8).

In many parts of the world, pharmaceutical companies are not mandated to publicly disclose the complete list of ingredients and excipients in individual drug formulations. This makes it often near-impossible to confidently identify specific culprits within drug formulations or select safe medications for patients with excipient allergy. In Hong Kong, patients with history of life-threatening reactions have been misdiagnosed with drug allergies prior to confirming excipients as culprits (9). To address this, we developed an excipient database based on our territory-wide drug allergy registry in Hong Kong to help facilitate the diagnosis and management of excipient allergy in our region—the Hong Kong-Excipient Registry (HK-ER). By systematically compiling all publicly available excipient data, HK-ER aimed to identify excipients in all registered formulations of the most commonly reported drug allergy culprits. We present findings of a pilot study using HK-ER to identify drug formulations, using PEG as an example for selecting safe medications for patients with excipient allergy.

## 2 Materials and methods

HK-ER was created by compiling the excipient lists pertaining to all available oral and injectable formulations of each of the top 50 most reported drug allergies in Hong Kong (either by extracting from product inserts or contacting individual pharmaceutical companies). The top 50 reported drug culprits, as of 31st December 2022, was retrieved from a unified, population-wide reported drug allergy registry representing 7.3 million (99%) of the Hong Kong population as previously described (10). In view of the recent relevance for mRNA vaccines, PEG was selected for this pilot study (5). Depending on the available excipient information from HK-ER, all drug formulations were then categorised as either “contains PEG”, “does not contain PEG”, or “unknown”. The relationship between the total number of formulations for a drug and the percentage of drugs with excipient information available was analysed using Spearman correlation, with two-sided *p*-values <0.05 considered statistically significant.

## 3 Results

**Figure 1:**
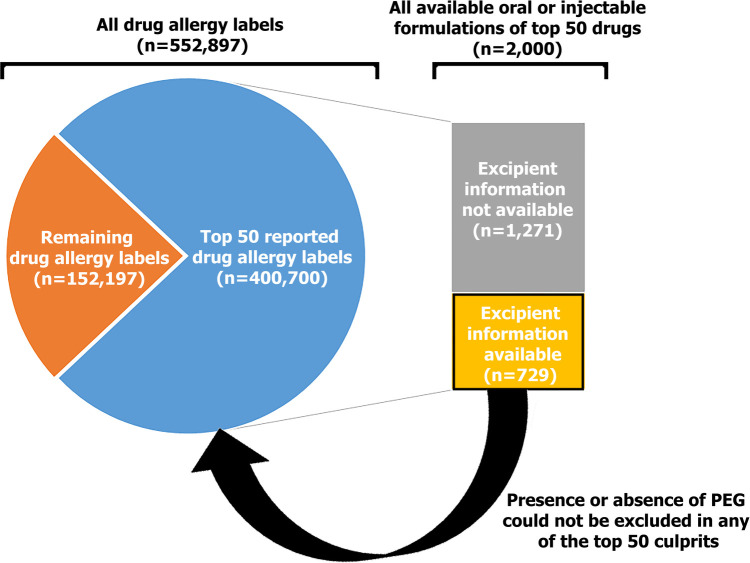
Breakdown of the formulations and excipient information for PEG available of the top 50 reported drug allergies in Hong Kong.

The top 50 reported drug allergy culprits in Hong Kong and detailed list of the HK-ER are shown in Table 1 and Supplementary Text 1, respectively. These 50 drugs accounted for 72.5% (400,700/552,897 patients) of all suspected drug allergy reactions in our region (Figure 1). In total, there were 2,000 unique formulations of these 50 drugs registered in Hong Kong. Complete excipient information was available for 36.5% (729/2,000) of formulations. The total number of formulations for a drug was associated with the proportion of formulations with excipient information available (Spearman correlation coefficient: 0.466, *p* = 0.001) (Table 1). Out of those 729 formulations, we were able to confirm that 109 (15.0%) contained PEG while the remaining 620 (85.0%) did not contain PEG. However, excipient information was not available for the remaining 1,271 (63.6%) formulations and we were unable to confirm presence or absence of PEG despite attempts at contacting registered pharmaceutical companies. After re-mapping all 729 formulations with available excipient information back into the original 50 drugs, we were unable to confirm the presence or absence of PEG in any single one of these drugs registered in Hong Kong (i.e., there were formulations with unknown excipient information for each of the top 50 allergy culprits).

**Table 1:** Top 50 reported drug allergy culprits in Hong Kong and the individual proportion of formulations with excipient information for PEG available.

Drug name	No. of formulations with excipient information/Total no. of formulations	Drug name (continued)	No. of formulations with excipient information/Total no. of formulations
Acetylsalicylic acid (aspirin)	7/18 (38.9%)	Sulphur	–^a^
Amoxicillin-clavulanate	0/4 (0.0%)	Clarithromycin	16/33 (48.5%)
Phenoxypenicillin	0/2 (0.0%)	Naproxen	4/19 (21.1%)
Amoxicillin	34/101 (33.7%)	Chloramphenicol	0/2 (0.0%)
Tetracycline	3/11 (27.3%)	Celecoxib	9/28 (32.1%)
Diclofenac	34/112 (30.4%)	Tramadol	15/42 (35.7%)
Ampicillin	8/27 (29.6%)	Ketorolac	0/6 (0.0%)
Mefenamic acid	27/74 (36.5%)	Hyoscine	22/49 (44.9%)
Cotrimoxazole	4/13 (30.8%)	Prochlorperazine	7/13 (53.8%)
Ibuprofen	28/78 (35.9%)	Metoclopramide	5/19 (26.3%)
Paracetamol	270/722 (37.4%)	Ofloxacin	4/11 (36.4%)
Cloxacillin	9/26 (34.6%)	Brimonidine	–^a^
Erythromycin	7/20 (35.0%)	Azithromycin	18/39 (46.2%)
Alcohol	2/8 (25.0%)	Acetaminophen-phenyltoloxamine	–^a^
Allopurinol	10/27 (37.0%)	Vancomycin	1/7 (14.3%)
Cefuroxime	10/27 (37.0%)	Ceftriaxone	11/24 (45.8%)
Benzylpenicillin	0/2 (0.0%)	Piperacillin + Tazobactam	10/16 (62.5%)
Ciprofloxacin	15/47 (31.9%)	Doxycycline	8/25 (32.0%)
Carbimazole	1/4 (25.0%)	Nifedipine	2/8 (25.0%)
Cephalexin	18/37 (48.6%)	Lisinopril	4/14 (28.6%)
Methyl salicylate compound	–^a^	Cefaclor	7/19 (36.8%)
Levofloxacin	8/32 (25.0%)	Amlodipine	25/79 (31.6%)
Metronidazole	9/32 (28.1%)	Simvastatin	9/40 (22.5%)
Phenytoin	0/4 (0.0%)	Carbamazepine	4/7 (57.1%)
Etoricoxib	10/18 (55.6%)	Indomethacin	34/54 (63.0%)
Median total number of formulations available for a drug: 22 (IQR: 10–39)			Spearman correlation coefficient: 0.466, *p* = 0.001**
Median percentage of formulations with excipient information available: 32.1 (IQR: 25.0–37.8)			

PEG, polyethylene glycol; IQR, inter-quartile range.

^a^ There were no or no longer oral or injectable formulations registered in Hong Kong for this drug.

*p < 0.05. **p < 0.01. ***p < 0.001.

## 4 Discussion

Deficiencies in drug ingredient listing impedes comprehensive drug allergy evaluation, undermining drug safety and public confidence in the healthcare system. The European Union has mandated the labelling of all excipients in the package leaflet for all medicines (11). Despite our past efforts to advocate for pharmaceutical legislation, transparent ingredient disclosure remains to be a persistent issue in our locality and many other parts of the world, and legislative changes have yet to materialise (6). Using PEG as an example, we were only able to confirm its presence or absence among 729 of 2,000 formulations investigated. This made it impossible to exclude or confirm presence of PEG in all formulations of any of the top 50 reported drug allergy culprits in Hong Kong. This problem also pertains to all possible excipients including other commonly implicated culprits of drug-associated allergies such as carboxymethylcellulose (a polymer derived from cellulose commonly used in barium contrast or corticosteroid formulations), gelatin (hydrolysed form of collagen, commonly used in tablets/capsules or colloid fluids) and polysorbate 80 (a common excipient found in injectable medications) (3, 12, 13). Further studies into the prevalence and impact of other excipient allergies are warranted; nonetheless, this study illustrates the danger and inability to select safe formulations for excipient allergy patients in our region. Interestingly, we identified that the number of available formulations for each drug was associated with higher proportion of excipient information available. Therefore, in regions where excipient listings are still not mandated, registration of more alternative formulations (especially with excipient data available) may be an effective way to facilitate excipient list disclosure overall. This may be a temporary measure to boost the availability of excipient data prior to establishment of mandatory excipient listings.

Limitations of this study include its observational nature and uncertain accuracy of reported drug allergies. Although we only included the top 50 drug allergy culprits as reported by physicians (rather than self-reported by patients), most suspected drug culprits were not verified and many reported “allergies” could be mislabelled (14). However, lack of excipient information further exacerbates the inability of comprehensive drug allergy evaluation and, therefore, it would be pertinent to make excipient listings available as soon as possible (9).

In conclusion, our findings further support the global need and urgency of mandatory drug ingredient listing. However, given the deficiencies in pharmaceutical legislation, excipient details were still unavailable in a substantial proportion of drug formulations despite attempts of our excipient database. Thus, we advocate for an international effort towards universal mandating ingredient disclosure and excipient listing for all registered drug formulations.

## Data Availability

The datasets presented in this article are not readily available because of ethical and privacy regulations. Requests to access the datasets should be directed to the corresponding author.

## References

[1] Caballero ML Krantz MS Quirce S Phillips EJ Stone CA Jr. Hidden dangers: recognizing excipients as potential causes of drug and vaccine hypersensitivity reactions. J Allergy Clin Immunol Pract. (2021) 9:2968–82. 10.1016/j.jaip.2021.03.002 33737254 PMC8355062

[2] Venturini Díaz M Vidal Oribe I D'Elia Torrence D Hernández Alfonso P Alarcón Gallardo E. New challenges in drug allergy: the resurgence of excipients. Curr Treat Options Allergy. (2022) 9:273–91. 10.1007/s40521-022-00313-6 35910462 PMC9308858

[3] Caballero ML Quirce S. Immediate hypersensitivity reactions caused by drug excipients: a literature review. J Investig Allergol Clin Immunol. (2020) 30:86–100. 10.18176/jiaci.0476 32327401

[4] Reker D Blum SM Steiger C Anger KE Sommer JM Fanikos J “Inactive” ingredients in oral medications. Sci Transl Med. (2019) 11(483):eaau6753. 10.1126/scitranslmed.aau6753 30867323 PMC7122736

[5] Khan DA Banerji A Blumenthal KG Phillips EJ Solensky R White AA Drug allergy: a 2022 practice parameter update. J Allergy Clin Immunol. (2022) 150:1333–93. 10.1016/j.jaci.2022.08.028 36122788

[6] Chiang V Leung ASY Au EYL Ho MHK Lee TH Wu AYY Updated consensus statements on COVID-19 vaccine allergy safety in Hong Kong. Asia Pac Allergy. (2022) 12:e8. 10.5415/apallergy.2022.12.e8 35174059 PMC8819424

[7] Kan AKC Wong TTH Chiang V Lau CS Li PH. Chronic spontaneous Urticaria in Hong Kong: clinical characteristics, real-world practice and implications for COVID-19 vaccination. Allergy Asthma Immunol Res. (2023) 15:32–42. 10.4168/aair.2023.15.1.32 36693356 PMC9880305

[8] Chiang V Saha C Yim J Au EYL Kan AKC Hui KSH The role of the allergist in coronavirus disease 2019 vaccine allergy safety: a pilot study on a “hub-and-spoke” model for population-wide allergy service. Ann Allergy Asthma Immunol. (2022) 129:308–12.e1. 10.1016/j.anai.2022.05.011 35605815 PMC9121691

[9] Li PH Yeung HHF Lau CS Au EYL. Excipient allergy and importance of complete allergy histories. J Allergy Clin Immunol Pract. (2020) 8:2122–3. 10.1016/j.jaip.2020.04.010 32499043

[10] Li PH Yeung HHF Lau CS Au EYL. Prevalence, incidence, and sensitization profile of beta-lactam antibiotic allergy in Hong Kong. JAMA Netw Open. (2020) 3:e204199. 10.1001/jamanetworkopen.2020.4199 32374398 PMC7203606

[11] European Commisson Directorate-General for Health and Food Safety. Excipients in the labelling and package leaflet of medicinal products for human use (2018).

[12] Li PH Wagner A Thomas I Watts TJ Rutkowski R Rutkowski K. Steroid allergy: clinical features and the importance of excipient testing in a diagnostic algorithm. J Allergy Clin Immunol Pract. (2018) 6:1655–61. 10.1016/j.jaip.2018.01.007 29408496

[13] Au EYL Mak HWF Yeung MHY Chiang V Lam K Wong JCY Ten-year outcomes of perioperative anaphylaxis workup study in Hong Kong (PAWS-HK): performance of diagnostic modalities. Ann Allergy Asthma Immunol. (2023) 130:752–9.e1. 10.1016/j.anai.2023.02.017 36842494

[14] Li PH Siew LQC Thomas I Watts TJ Ue KL Rutkowski K Beta-lactam allergy in Chinese patients and factors predicting genuine allergy. World Allergy Organ J. (2019) 12:100048. 10.1016/j.waojou.2019.100048 31692961 PMC6822230

