# Supplementary material for: Feasibility of a drug allergy registry-based excipient allergy database and call for universal mandatory drug ingredient disclosure: the case of PEG
Source: Front Allergy. 2024 Jan 16;4:1331036. doi: 10.3389/falgy.2023.1331036 (PMC10824824; doi:10.3389/falgy.2023.1331036)
Supplement: Supplementary file 1 [file Datasheet1.pdf]

Hong Kong-Excipient Registry (HK-ER)

	Generic Name	Product Name	Company	Registered No.	Contains PEG? (Yes/No/Unknown)
1	**ASPIRIN**	ACEPRIN TAB 100MG	HOVID LIMITED	HK-57543	Unknown
2		ALKA-SELTZER EFFER TAB 324MG LEMON FLAV	BAYER HEALTHCARE LIMITED	HK-44990	Unknown
3		ASCOT 81 ENTERIC-COATED TAB 81MG	NATURAL HEALTH RESOURCES COMPANY LIMITED	HK-59150	Yes
4		ASP TABLETS 100MG	CNW (HK) LTD	HK-62059	Unknown
5		ASPIRIN KUKJE GASTRO-RESISTANT TABLETS 100MG	SB PHARMA LIMITED	HK-67345	Unknown
6		ASPIRIN PROTECT 100 TAB (ENTERIC-COATED)	BAYER HEALTHCARE LIMITED	HK-42077	Unknown
7		ASPIRIN TAB 300MG (VIDA)	VICKMANS LABORATORIES LTD	HK-19389	No
8		ASPIRIN TAB 80MG (ORANGE)-SYNCO	SYNCO (H.K.) LIMITED	HK-06252	No
9		ASPIRIN TAB BP 300MG	ASIA CHEM IND LTD	HK-04324	Unknown
10		ASPIRIN TABLETS (ORANGE) 80MG	SYNCO (H.K.) LIMITED	HK-63686	No
11		BAYER ASPIRIN TAB 500MG	BAYER HEALTHCARE LIMITED	HK-47567	Unknown
12		BOKEY ENTERIC-MICROENCAPSULATED CAP100MG	YUNG SHIN CO LTD	HK-41106	Unknown
13		CARDIPRIN 100 TAB 100MG	RECKITT BENCKISER HONG KONG LTD	HK-34034	No
14		COPLAVIX TAB 75MG/100MG	SANOFI HONG KONG LIMITED	HK-59807	Unknown
15		CORTAL TAB FOR ADULTS	EUROPHARM LAB CO LTD	HK-02227	No
16		FUSEN E.M. CAP 100MG	HITPHARM PHARMACEUTICAL CO LTD	HK-49463	Unknown
17		PROPIRIN ENTERIC COATED TAB 100MG	CHRISTO PHARM LTD	HK-48244	Yes
18		SAMJIN ASPIRIN ENTERIC-COATED TABLETS 100MG	LSB (HK) LIMITED	HK-65427	Unknown
19	**AUGMENTIN**	AUGMENTIN FOR IV INJ 1.2G/VIAL	GLAXOSMITHKLINE LIMITED	HK-27421	Unknown
20		AUGMENTIN FOR SYRUP 156MG/5ML	GLAXOSMITHKLINE LIMITED	HK-24658	Unknown
21		AUGMENTIN POWDER FOR SYRUP 457MG/5ML	GLAXOSMITHKLINE LIMITED	HK-42735	Unknown
22		AUGMENTIN TAB 1G	GLAXOSMITHKLINE LIMITED	HK-42252	Unknown
23	**PHENOXYMETHYLPENICILLIN**	PENICILLIN V ELIXIR 125MG/5ML	DCH AURIGA (HONG KONG) LIMITED - UNIVERSAL DIVISION	HK-33029	Unknown
24		PENICILLIN VK TAB 250MG	DCH AURIGA (HONG KONG) LIMITED - UNIVERSAL DIVISION	HK-33028	Unknown
25	**AMOXYCILLIN**	ALVOCLAV POWDER FOR SOLUTION FOR INJECTION 1000MG/200MG	HONG KONG MEDICAL SUPPLIES LTD	HK-64389	No
26		AMK 156 POWDER FOR ORAL SUSPENSION 156.25MG/5ML	HIND WING CO LTD	HK-64465	Unknown
27		AMK TAB 1000MG	HIND WING CO LTD	HK-60547	Unknown
28		AMOKSIKLAV ORAL SUSPENSION	NOVARTIS PHARMACEUTICALS (HK) LIMITED	HK-41947	No
29		AMOKSIKLAV POWDER FOR ORAL SUSPENSION 457MG/5ML	NOVARTIS PHARMACEUTICALS (HK) LIMITED	HK-62668	No
30		AMOKSIKLAV TAB 1000MG	NOVARTIS PHARMACEUTICALS (HK) LIMITED	HK-61455	No
31		AMON-I CAP 250MG	VICKMANS LABORATORIES LTD	HK-50577	No
32		AMON-S CAP 250MG	VICKMANS LABORATORIES LTD	HK-50576	No
33		AMOX CAP 250MG	VICKMANS LABORATORIES LTD	HK-50832	No
34		AMOXICAP 250 CAP	HOVID LIMITED	HK-35653	Unknown
35		AMOXICILLIN AND CLAVULANIC ACID POWDER FOR ORAL SUSPENSION 200/28.5MG	BRIGHT FUTURE PHARMACEUTICALS FACTORY O/B BRIGHT FUTURE PHARMACEUTICAL LABORATORIES LIMITED	HK-62343	Unknown
36		AMOXICILLIN AND CLAVULANIC ACID POWDER FOR SOLUTION FOR INJECTION 1.2G (NORTH CHINA PHARMACEUTICAL)	JINDUN PHARMA (H.K.) LIMITED	HK-61355	Unknown
37		AMOXICILLIN AND CLAVULANIC ACID TABLETS 375MG	JINDUN PHARMA (H.K.) LIMITED	HK-64907	Unknown
38		AMOXICILLIN CAP 250MG	YUNG SHIN CO LTD	HK-34413	Unknown
39		AMOXICILLIN CAP 250MG	DCH AURIGA (HONG KONG) LIMITED - UNIVERSAL DIVISION	HK-55780	Unknown
40		AMOXICILLIN CAPSULES 500MG	WINGS PHARMACEUTICAL LTD	HK-62144	No
41		AMOXICILLIN ORAL SUSPENSION BP 125MG/5ML	DCH AURIGA (HONG KONG) LIMITED - UNIVERSAL DIVISION	HK-55778	Unknown
42		AMOXICILLIN POWDER FOR SOLUTION FOR IM/IV INJECTION 1G (NORTH CHINA PHARMACEUTICAL)	JINDUN PHARMA (H.K.) LIMITED	HK-61172	Unknown
43		AMOXICILLIN SODIUM AND CLAVULANATE POTASSIUM FOR INJ 0.6G (SHANDONG LUKANG)	JINDUN PHARMA (H.K.) LIMITED	HK-58181	Unknown
44		AMOXICILLIN/CLAVULANIC ACID-TEVA POWDER FOR SOLUTION FOR INJECTION/INFUSION 1000MG/200MG	THE INTERNATIONAL MEDICAL COMPANY LIMITED	HK-63481	Unknown
45		AMOXIGRAN GRANULES 125MG/5ML	HOVID LIMITED	HK-37662	Unknown
46		AMOXITAB 250 FLAVOURED TAB 250MG	HOVID LIMITED	HK-43431	Unknown
47		AMOXON-G CAP 500MG	VICKMANS LABORATORIES LTD	HK-48496	No
48		AMOXON-P CAP 500MG	VICKMANS LABORATORIES LTD	HK-48497	No
49		AMOXON-S CAP 500MG	VICKMANS LABORATORIES LTD	HK-50449	No
50		AMOXY DRY SYRUP 125MG PER PACKET	THE UNITED LABORATORIES LTD	HK-43894	Unknown
51		AMOXYCILLIN 2 CAP 250MG	VICKMANS LABORATORIES LTD	HK-32898	No
52		AMOXYCILLIN 500 CAP M/Y (BRIGHT FUTURE)	BRIGHT FUTURE PHARMACEUTICALS FACTORY O/B BRIGHT FUTURE PHARMACEUTICAL LABORATORIES LIMITED	HK-37315	Unknown
53		AMOXYCILLIN 500MG CAP	VICKMANS LABORATORIES LTD	HK-37001	No
54		AMOXYCILLIN CAP 250MG (BRIGHT FUTURE)	BRIGHT FUTURE PHARMACEUTICALS FACTORY O/B BRIGHT FUTURE PHARMACEUTICAL LABORATORIES LIMITED	HK-39546	Unknown
55		AMOXYCILLIN CAP 500MG (UNITED LAB)	THE UNITED LABORATORIES LTD	HK-44537	Unknown
56		AMOXYCILLIN CAPSULE 250MG	NEW HEALTHY MEDICAL LIMITED	HK-64168	Unknown
57		AMOXYCILLIN DRY SUSPENSION POWDER 250MG/SACHET (BRIGHT FUTURE)	BRIGHT FUTURE PHARMACEUTICALS FACTORY O/B BRIGHT FUTURE PHARMACEUTICAL LABORATORIES LIMITED	HK-55934	Unknown
58		AMOXYCILLIN DRY SYRUP 125MG/5ML	VICKMANS LABORATORIES LTD	HK-32897	No
59		AMOXYCILLIN GRANULES FOR SUSPENSION 250MG/5ML	VICKMANS LABORATORIES LTD	HK-55828	No
60		AMOXYCILLIN GRANULES FOR SYRUP 125MG/5ML	VICKMANS LABORATORIES LTD	HK-35591	No
61		AMOXYCILLIN SOD/CLAVULANATE POT F/ INJ 1.2G (ZHUHAI UNITED LAB)	THE UNITED LABORATORIES LTD	HK-52053	Unknown
62		AMYN-250 CAP 250MG	NIDOWAY INVESTMENT LTD	HK-44497	Unknown
63		CLABAT FORTE DRY SYRUP 312.5MG/5ML	NATURAL HEALTH RESOURCES COMPANY LIMITED	HK-50439	No
64		CLAMOVID 625 TAB 625MG	HOVID LIMITED	HK-50621	Unknown
65		CLAMOVID BID 1000 TAB 1000MG	HOVID LIMITED	HK-57828	Unknown
66		CLAMOVID BID FORTE GRANULES 457MG/5ML	HOVID LIMITED	HK-57827	Unknown
67		CLAMOVID BID GRANULES FOR SUSP 228MG/5ML	HOVID LIMITED	HK-57826	Unknown
68		CLAMOVID GRANULES 156.25MG/5ML	HOVID LIMITED	HK-50620	Unknown
69		CLAMOXIN DRY SYRUP	JULIUS CHEN & CO (HK) LTD	HK-56453	Unknown
70		CLAMOXIN DUO DRY SYRUP	JULIUS CHEN & CO (HK) LTD	HK-56268	Unknown
71		CLAMOXIN TAB 625MG	JULIUS CHEN & CO (HK) LTD	HK-59166	Unknown
72		CLAVAMOX 125 DRY SYRUP 125MG/5ML	HANG LUNG TRADING (H.K.) CO	HK-42003	Unknown
73		CLAVOMID 375 TABLETS 375MG	HEALTHCARE PHARMASCIENCE LIMITED	HK-67038	Unknown
74		CLAVOMID 625 FILM-COATED TABLETS 625MG	HEALTHCARE PHARMASCIENCE LIMITED	HK-63125	Unknown
75		CLEDOMOX 1000 TABLETS	SWEDISH TRADING COMPANY LIMITED	HK-62186	Unknown
76		CLEDOMOX 156.25 ORAL SUSPENSION 156.25MG/5ML	SWEDISH TRADING COMPANY LIMITED	HK-64216	Unknown
77		CLEDOMOX 375 TABLETS	SWEDISH TRADING COMPANY LIMITED	HK-62184	Unknown
78		CLOXAMOX CAP	VICKMANS LABORATORIES LTD	HK-50448	No
79		CO-AMOXICLAV DRY SUSP 200/28.5MG (BF)	BRIGHT FUTURE PHARMACEUTICALS FACTORY O/B BRIGHT FUTURE PHARMACEUTICAL LABORATORIES LIMITED	HK-50392	Unknown
80		CO-AMOXICLAV TAB 1000MG (BRIGHT FUTURE)	BRIGHT FUTURE PHARMACEUTICALS FACTORY O/B BRIGHT FUTURE PHARMACEUTICAL LABORATORIES LIMITED	HK-49813	Unknown
81		COMPLICILLIN CAP	HANG LUNG TRADING (H.K.) CO	HK-55107	Unknown
82		COMPLIPEN CAP	HANG LUNG TRADING (H.K.) CO	HK-35276	Unknown
83		CURAM 1000MG+200MG FOR INJ	NOVARTIS PHARMACEUTICALS (HK) LIMITED	HK-56403	No
84		CURAM FOR ORAL SUSPENSION 457MG/5ML	NOVARTIS PHARMACEUTICALS (HK) LIMITED	HK-56738	No
85		CURAM TAB 1000MG	NOVARTIS PHARMACEUTICALS (HK) LIMITED	HK-56737	No
86		DICLOMOX CAP	BRIGHT FUTURE PHARMACEUTICALS FACTORY O/B BRIGHT FUTURE PHARMACEUTICAL LABORATORIES LIMITED	HK-53646	Unknown
87		EDAMOX 500 CAP 500MG	HEALTHCARE PHARMASCIENCE LIMITED	HK-44885	Unknown
88		FLEMING FOR ORAL SUSPENSION 457MG/5ML	APT PHARMA LIMITED	HK-56072	Unknown
89		FLEMING TAB 1G	APT PHARMA LIMITED	HK-52242	Unknown
90		KOACT POWDER FOR ORAL SUSPENSION 250MG/62.5MG/5ML	HANG LUNG TRADING (H.K.) CO	HK-66480	Unknown
91		LIDINGSHA FOR I.V. INJ 1.2G	JINDUN PHARMA (H.K.) LIMITED	HK-51188	Unknown
92		MA-AMOXYCILLIN CAP 250MG	VICKMANS LABORATORIES LTD	HK-47446	No
93		MEDOMOX-500 CAP 500 MG	JULIUS CHEN & CO (HK) LTD	HK-54077	Unknown
94		MOXARIN 500 CAP 500MG	CEUTICAL TRADING COMPANY LIMITED	HK-39419	Unknown
95		MOXCILLIN CAP 250MG	DELTAPHARM LIMITED	HK-44042	Unknown
96		MOXCILLIN SYRUP 250MG/5ML	DELTAPHARM LIMITED	HK-41596	Unknown
97		MOXCIN CAP 250MG	TRENTON-BOMA LTD	HK-49694	No
98		MOXICLAV 375 TAB	STAR MEDICAL SUPPLIES LTD	HK-42573	Yes
99		MOXICLAV FORTE POWDER FOR ORAL SUSP	STAR MEDICAL SUPPLIES LTD	HK-43109	No

100		MOXICLAV POWDER FOR ORAL SUSP	STAR MEDICAL SUPPLIES LTD	HK-43108	No
101		MOXICLAV POWDER FOR SOLUTION FOR INJECTION OR INFUSION 1.2G	STAR MEDICAL SUPPLIES LTD	HK-60976	No
102		MOXICLAV TAB 1G	STAR MEDICAL SUPPLIES LTD	HK-54868	Yes
103		MOXILEN 250 CAP 250MG	MEDOCHEMIE (HONG KONG) LIMITED	HK-25156	Unknown
104		MOXILEN FORTE DRY PDR FOR ORAL 250MG/5ML	STAR MEDICAL SUPPLIES LTD	HK-25155	No
105		MOXIPEN SUSPENSION 125MG/5ML	FP HEALTHCARE LIMITED	HK-36163	Unknown
106		NEOMOX CAP 500MG	VICKMANS LABORATORIES LTD	HK-47237	No
107		OSPAMOX CAP 250 MG	NOVARTIS PHARMACEUTICALS (HK) LIMITED	HK-32357	No
108		PANMOX CAP 250MG	VAST RESOURCES PHARMACEUTICAL LTD	HK-52582	Unknown
109		PENCLA DUO POWDER FOR ORAL SUSPENSION 200/28.5MG/5ML	LSB (HK) LIMITED	HK-65426	Unknown
110		PENCLA DUO TABLETS 1 G	LSB (HK) LIMITED	HK-63944	Unknown
111		PENCLA TABLETS 250MG/125MG	LSB (HK) LIMITED	HK-63945	Unknown
112		PHARMANIAGA CO-AMOXICLAV TAB 375MG	HEALTHCARE PHARMASCIENCE LIMITED	HK-52769	Unknown
113		PROMOX CAP 250MG	APT PHARMA LIMITED	HK-52758	Unknown
114		PROMOX ORAL SUSPENSION 250MG/5ML	APT PHARMA LIMITED	HK-54864	Unknown
115		RAPICLAV-1G TABLETS 875MG/125MG	EUGENPHARM INTERNATIONAL LIMITED	HK-63094	Unknown
116		REICHAMOX CAP 250MG	MEDREICH FAR EAST LIMITED	HK-52873	Unknown
117		SYNCO AMOXYCILLIN CAP 250MG (GREY/CARAMEL)	VICKMANS LABORATORIES LTD	HK-54649	No
118		SYNTOCLAV 375 TAB (FILM COATED)	CNW FAR EAST LIMITED	HK-46883	Unknown
119		SYNTOCLAV FOR SUSP. 156.25MG/5ML	CNW FAR EAST LIMITED	HK-46931	Unknown
120		SYNTOCLAV FORTE FOR SUSP. 312.5MG/5ML	CNW FAR EAST LIMITED	HK-46885	Unknown
121		T.V. MOX CAP 500MG	GAILY PHARMACEUTICAL COMPANY LIMITED	HK-56083	Unknown
122		UNI-AMOCIN CAP 250MG	THE UNITED LABORATORIES LTD	HK-35473	Unknown
123		UNICIL FORTE CAP	UNICORN LABORATORIES O/B AMERICAN UNICORN LABORATORIES LIMITED	HK-35711	Unknown
124		UNIMOX 500 CAP 500MG	HEALTH ALLIANCE INTERNATIONAL CO LTD	HK-45379	No
125		YUCLA TABLETS 375MG	JACOBSON MEDICAL (HONG KONG) LTD	HK-67266	No
126	**TETRACYCLINE**	APO-TETRA CAP 250MG	HIND WING CO LTD	HK-34922	Unknown
127		MEDOCYCLINE CAP 250MG	STAR MEDICAL SUPPLIES LTD	HK-30208	No
128		MEYERCYCLINE CAP 250MG	MEYER PHARMACEUTICALS LTD	HK-08180	Unknown
129		NEO-CYCLIN CAP 250MG	NEOCHEM PHARMACEUTICAL LABORATORIES LTD.	HK-05419	Unknown
130		NICE-TETRA CAP 250MG	NICE LABORATORIES LTD	HK-07350	Unknown
131		TETRACYCLINE CAP 250MG RED/GREY	CHRISTO PHARM LTD	HK-22271	No
132		OXYLIM INJ 50MG/ML	ATLANTIC PHARMACEUTICAL LIMITED	HK-44440	Unknown
133		TETRACAP 250 CAP 250MG	HOVID LIMITED	HK-36081	Unknown
134		TETRACLIN CAP 250MG ORANGE/YELLOW	NEOCHEM PHARMACEUTICAL LABORATORIES LTD.	HK-20019	Unknown
135		TETRACYCLINE CAP 250MG	CHRISTO PHARM LTD	HK-35714	No
136		TETRALYSAL CAP 300MG	GALDERMA HONG KONG LIMITED	HK-06231	Unknown
137	**DICLOFENAC**	A-FENAC K50 TAB 50MG	DELTAPHARM LIMITED	HK-58266	Unknown
138		ALMIRAL 50 TAB 50MG ENTERIC COATED	MEDOCHEMIE (HONG KONG) LIMITED	HK-29260	Unknown
139		ALMIRAL INJ 25MG/ML	MEDOCHEMIE (HONG KONG) LIMITED	HK-35076	Unknown
140		ALMIRAL TAB ENTERIC-COATED 25MG	STAR MEDICAL SUPPLIES LTD	HK-25677	No
141		ANALPAN INJ 25MG/ML	WINGS PHARMACEUTICAL LTD	HK-42749	No
142		APO-DICLO TAB 25MG (ENTERIC COATED)	HIND WING CO LTD	HK-36326	Unknown
143		ARSPAN DICLOFENAC SODIUM TABLETS 25MG	WELLDONE PHARMACEUTICALS LIMITED	HK-67392	Unknown
144		ARTHAREN E.C. TAB 25MG	TRENTON-BOMA LTD	HK-49680	Yes
145		CATAFLAM TAB 25MG	NOVARTIS PHARMACEUTICALS (HK) LIMITED	HK-35993	Yes
146		CLOFEC TAB 25MG	ATLANTIC PHARMACEUTICAL LIMITED	HK-35616	Unknown
147		CLOFENAC SR TAB 100MG	HOVID LIMITED	HK-47041	Unknown
148		CLOFENAC-50 TAB 50MG (ENTERIC-COATED)	HOVID LIMITED	HK-38583	Unknown
149		DICLO 25 ENTERIC COATED TAB 25MG	WAI LUN TRADING CO	HK-58261	Unknown
150		DICLO 50 TAB 50MG (ENTERIC-COATED)	WAI LUN TRADING CO	HK-60068	Unknown
151		DICLOFEN ENTERIC-MICRO-ENCAPSULED CAPS 50MG	VAST RESOURCES PHARMACEUTICAL LTD	HK-59849	Unknown
152		DICLOFEN TAB 25MG	EUROPHARM LAB CO LTD	HK-30746	Yes
153		DICLOFEN TAB 50MG	JEAN-MARIE PHARMACAL CO LTD	HK-30745	Yes
154		DICLOFEN TAB 50MG ENTERIC COATED	EUROPHARM LAB CO LTD	HK-34963	Yes
155		DICLOFENAC 100 STADA RETARD TAB 100MG	HONG KONG MEDICAL SUPPLIES LTD	HK-38246	No
156		DICLOFENAC E.C. TAB 50MG	VAST RESOURCES PHARMACEUTICAL LTD	HK-53181	Unknown
157		DICLOFENAC E.C. TAB 50MG (CHIN TENG)	HITPHARM PHARMACEUTICAL CO LTD	HK-49110	Unknown
158		DICLOFENAC POTASSIUM TAB 50MG	SYNCO (H.K.) LIMITED	HK-56424	No
159		DICLOFENAC SOD INJ 25MG/ML	STAR MEDICAL SUPPLIES LTD	HK-25362	No
160		DICLOFENAC SODIUM E.C. TAB 50MG	WELLDONE PHARMACEUTICALS LIMITED	HK-54684	Unknown
161		DICLOFENAC SODIUM ENTERIC FC TAB (SHOU CHAN)	WEIJIAN MEDICAL COMPANY LIMITED	HK-45384	Unknown
162		DICLOFENAC SODIUM INJ 75MG/3ML	STAR MEDICAL SUPPLIES LTD	HK-54405	No
163		DICLOFENACO CINFA ENTERIC-COATED TAB 50MG	REICH PHARM LIMITED	HK-51134	Unknown
164		DICLOGESIC 50 TAB 50MG (E.C.)	PRIMAL CHEMICAL CO LTD	HK-44447	Unknown
165		DICLOREN E.M. CAP 50MG	YAT SENG TRADING CO	HK-55072	Unknown
166		DICLOSET TAB 25MG ENTERIC COATED	EUROPHARM LAB CO LTD	HK-42972	Yes
167		DICOFEN SR TAB 100MG	MEDREICH FAR EAST LIMITED	HK-55484	Unknown
168		DIFEN GASTRO-RESISTANT TABLETS 50MG	HITPHARM PHARMACEUTICAL CO LTD	HK-65612	Unknown
169		DIFENA INJ 25MG/ML	KAI YUEN PHARMACEUTICAL CO	HK-29257	Unknown
170		DIFENA TAB 25MG ENTERIC FILM COATED	KAI YUEN PHARMACEUTICAL CO	HK-33067	Unknown
171		DIFENA TAB 50MG ENTERIC COATED	KAI YUEN PHARMACEUTICAL CO	HK-33899	Unknown
172		DIFENAC INJ. 75MG/3ML	DELTAPHARM LIMITED	HK-55928	Unknown
173		DIFENOL 25 TAB 25MG	EUROPHARM LAB CO LTD	HK-43450	Yes
174		DIFENOL TAB 100MG ER	APT PHARMA LIMITED	HK-54303	Unknown
175		DIFISAL TAB 50MG	NIDOWAY INVESTMENT LTD	HK-45004	Unknown
176		DIFLAM 50 TAB 50MG	NATURAL HEALTH RESOURCES COMPANY LIMITED	HK-59733	No
177		DILTAREN DICLOFENAC SODIUM GASTRO-RESISTANT TABLETS 50MG	WELLDONE PHARMACEUTICALS LIMITED	HK-67187	Unknown
178		DINAC ENTERIC COATED TABLETS 25MG	VAST RESOURCES PHARMACEUTICAL LTD	HK-64402	Unknown
179		DYNAPAR DICLOFENAC SODIUM AND PARACETAMOL TAB	EVERCARE PHARMACEUTICAL CO. LTD.	HK-59693	Unknown
180		DYNAPAR EC ENTERIC COATED TABLETS 50MG	EVERCARE PHARMACEUTICAL CO. LTD.	HK-62142	Unknown
181		DYNAPAR SR 100 TAB 100MG	EVERCARE PHARMACEUTICAL CO. LTD.	HK-59692	Unknown
182		EUNAC SR/F.C. TAB 100MG	SYNMOSA BIOPHARMA (HONG KONG) COMPANY LIMITED	HK-52467	Unknown
183		EUROFENAC TAB 50MG	EUROPHARM LAB CO LTD	HK-41993	No
184		FENAC TAB 25MG (ENTERIC COATED)	NATURAL HEALTH RESOURCES COMPANY LIMITED	HK-47018	No
185		FENAC-50 TAB 50MG	PRIMAL CHEMICAL CO LTD	HK-47145	Unknown
186		FENADIUM ENTERIC-COATED TAB 25MG	PRIMAL CHEMICAL CO LTD	HK-55034	Unknown
187		FORMIN INJ 25MG/ML	YAT SENG TRADING CO	HK-46910	Unknown
188		GASICA 25 ENTERIC COATED TABLETS 25MG	LEAMYK INVESTMENT LIMITED	HK-66084	Unknown
189		HOULUONING CAP 50MG	UNICORN LABORATORIES O/B AMERICAN UNICORN LABORATORIES LIMITED	HK-45420	Unknown
190		INFLANAC TAB 25MG ENTERIC COATED	MEKIM LTD	HK-24838	Unknown
191		JOYISH DICLOFENAC SODIUM TABLETS 25MG	WELLDONE PHARMACEUTICALS LIMITED	HK-67393	Unknown
192		KJ DICLOFEN ENTERIC FILM COATED TABLETS 25MG	HITPHARM PHARMACEUTICAL CO LTD	HK-64032	Unknown
193		KOJAR-DICLOFEN TAB 50MG	JULIUS CHEN & CO (HK) LTD	HK-47711	Unknown
194		LESFLAM 25 TAB 25MG	HEALTH ALLIANCE INTERNATIONAL CO LTD	HK-53943	Yes
195		LOPPER CAP	ADVANCE PHARMACEUTICAL COMPANY LIMITED	HK-48432	Unknown
196		NEO-CLOFEN TAB 25MG	EUROPHARM LAB CO LTD	HK-45459	No
197		NEUTALIN CAP	ADVANCE PHARMACEUTICAL COMPANY LIMITED	HK-48431	Unknown
198		OCIPIL DICLOFENAC SODIUM GASTRO-RESISTANT TABLETS 50MG	WELLDONE PHARMACEUTICALS LIMITED	HK-67188	Unknown
199		OLFEN-100 SR DEPOCAP 100MG SUST-RELEASE	CEUTICAL TRADING COMPANY LIMITED	HK-30019	Unknown
200		OLFEN-50 LACTAB 50MG	CEUTICAL TRADING COMPANY LIMITED	HK-30018	Unknown
201		OLFEN-75 INJ 75MG/2ML	CEUTICAL TRADING COMPANY LIMITED	HK-30020	Unknown
202		PAINOFF TAB 50MG	EUROPHARM LAB CO LTD	HK-42272	Yes
203		PAINSTOP CAP 25MG	WILCOME PHARMACEUTICAL CO LTD	HK-18528	Unknown
204		PAINSTOP INJ 25MG/ML	WILCOME PHARMACEUTICAL CO LTD	HK-41026	Unknown
205		PARNAC TAB	MEYER PHARMACEUTICALS LTD	HK-39332	Unknown
206		PHARMA DICLOFENAC SODIUM TAB 50MG EC	JULIUS CHEN & CO (HK) LTD	HK-44927	Unknown
207		REMAFEN TAB 25MG	FP HEALTHCARE LIMITED	HK-35268	Unknown
208		REMETHAN 25 TAB 25MG (ENTERIC COATED)	HEALTHCARE PHARMASCIENCE LIMITED	HK-45116	Unknown
209		REN ENTERIC MICROENCAPSULATED CAP 50MG	YUNG SHIN CO LTD	HK-38899	Unknown
210		REN ENTERIC-COATED TAB 25MG	YUNG SHIN CO LTD	HK-38900	Unknown

211		REN INJ 25MG/ML	YUNG SHIN CO LTD	HK-38901	Unknown
212		RHEMOFENAX CAP 25MG	WINGS PHARMACEUTICAL LTD	HK-42680	No
213		SAWTO E.M. CAP 50MG	HITPHARM PHARMACEUTICAL CO LTD	HK-49143	Unknown
214		SCANTAREN 50 TAB 50MG	HANG LUNG TRADING (H.K.) CO	HK-47677	Unknown
215		SYNFENAC TAB 100MG SR	APT PHARMA LIMITED	HK-55960	Unknown
216		TAKS TAB 50MG	CEUTICAL TRADING COMPANY LIMITED	HK-57633	Unknown
217		TAKS TAB ENTERIC-COATED 25MG	CEUTICAL TRADING COMPANY LIMITED	HK-26005	Unknown
218		TAPAIN INJ	HEALTHCARE PHARMASCIENCE LIMITED	HK-50756	Unknown
219		TENDUM CAP	ADVANCE PHARMACEUTICAL COMPANY LIMITED	HK-48433	Unknown
220		U-TAREN TAB 25MG	EUROPHARM LAB CO LTD	HK-29487	Yes
221		UMERAN SR 100 TAB 100MG	STAR MEDICAL SUPPLIES LTD	HK-60718	Yes
222		UMERAN-50 TAB 50MG	STAR MEDICAL SUPPLIES LTD	HK-60944	Yes
223		UNIREN ENTERIC-COATED TAB 25MG	HEALTH ALLIANCE INTERNATIONAL CO LTD	HK-43636	Yes
224		VARTELON TAB 25MG	CHRISTO PHARM LTD	HK-34581	Yes
225		VARTELON-50 ENTERIC-COATED TAB 50MG	CHRISTO PHARM LTD	HK-47303	Yes
226		VETIN INJ 25MG/ML	KAI YUEN PHARMACEUTICAL CO	HK-37818	Unknown
227		VICLOFENAC ENTERIC-COATED TAB 25MG	EUROPHARM LAB CO LTD	HK-51286	Yes
228		VIGOLIN CAP 50MG	UNICORN LABORATORIES O/B AMERICAN UNICORN LABORATORIES LIMITED	HK-45423	Unknown
229		VOLNA-K F.C. TAB 25MG	KAI YUEN PHARMACEUTICAL CO	HK-51590	Unknown
230		VOLTA ENTERIC FILM COATED TAB 25MG	GAILY PHARMACEUTICAL COMPANY LIMITED	HK-53579	Unknown
231		VOLTAREN ENTERIC COATED TAB 25MG (TURKEY)	NOVARTIS PHARMACEUTICALS (HK) LIMITED	HK-61557	Yes
232		VOLTAREN INJ 25 MG/ML	NOVARTIS PHARMACEUTICALS (HK) LIMITED	HK-00160	No
233		VOLTAREN SR 100 TAB 100MG SLOW-RELEASE	NOVARTIS PHARMACEUTICALS (HK) LIMITED	HK-15318	Yes
234		VOLTAREN SR PROLONGED-RELEASE TABLETS 100MG	NOVARTIS PHARMACEUTICALS (HK) LIMITED	HK-66111	Yes
235		VOLTAREN TAB 25 MG ENTERIC COATED	NOVARTIS PHARMACEUTICALS (HK) LIMITED	HK-00170	Yes
236		VOLTON-CR CAP 100MG	BRIGHT FUTURE PHARMACEUTICALS FACTORY O/B BRIGHT FUTURE PHARMACEUTICAL LABORATORIES LIMITED	HK-43808	Unknown
237		VOREN CAP 50MG (ENTERIC MICROENCAPSULATED)	YUNG SHIN CO LTD	HK-40171	Unknown
238		VOREN SR TAB 100MG	YUNG SHIN CO LTD	HK-40427	Unknown
239		VOREN TAB 25MG (ENTERIC COATED)	YUNG SHIN CO LTD	HK-40172	Unknown
240		VOTALEN TAB 50MG	EUROPHARM LAB CO LTD	HK-42304	No
241		VOTAN SR F.C. TAB 100MG "S.T."	JACOBSON MARKETING LIMITED	HK-58438	No
242		WILLIPO DICLOFENAC SODIUM GASTRO-RESISTANT TABLETS 50MG	WELLDONE PHARMACEUTICALS LIMITED	HK-67189	Unknown
243		XOEASE CAP 50MG	UNICORN LABORATORIES O/B AMERICAN UNICORN LABORATORIES LIMITED	HK-45422	Unknown
244		XTRA CAP 50MG	UNICORN LABORATORIES O/B AMERICAN UNICORN LABORATORIES LIMITED	HK-45419	Unknown
245		YUGOKIN CAP 50MG	UNICORN LABORATORIES O/B AMERICAN UNICORN LABORATORIES LIMITED	HK-45421	Unknown
246		ZOLASE CAP 50MG	UNICORN LABORATORIES O/B AMERICAN UNICORN LABORATORIES LIMITED	HK-45418	Unknown
247		ZOLTEROL SR TAB 100MG	PHARMALINK HEALTHCARE LTD	HK-44412	Unknown
248		ZUCON TAB 25MG	VAST RESOURCES PHARMACEUTICAL LTD	HK-52573	Unknown
249	**AMPICILLIN**	A P CAP 250MG	THE UNITED LABORATORIES LTD	HK-10610	Unknown
250		AMPICILLIN 500 CAP 500MG	APT PHARMA LIMITED	HK-40521	Unknown
251		AMPICILLIN CAP 500MG	VAST RESOURCES PHARMACEUTICAL LTD	HK-52442	Unknown
252		AMPICILLIN CAP 500MG (ATHLONE)	DCH AURIGA (HONG KONG) LIMITED - UNIVERSAL DIVISION	HK-51371	Unknown
253		AMPICILLIN CAP 500MG (UNITED LAB)	THE UNITED LABORATORIES LTD	HK-44538	Unknown
254		AMPICILLIN POWDER FOR SOLUTION FOR INJECTION 500MG	CEUTICAL TRADING COMPANY LIMITED	HK-63787	Unknown
255		AMPICILLIN SODIUM FOR INJ 0.5G	REGAL MEDICAL HEALTHCARE LIMITED	HK-56898	Unknown
256		AMPICLOXA-500 CAP	NIDOWAY INVESTMENT LTD	HK-43969	Unknown
257		AMPIK-250 CAP 250MG	NIDOWAY INVESTMENT LTD	HK-44495	Unknown
258		APT-AMPICILLIN CAP 250MG	APT PHARMA LIMITED	HK-40704	Unknown
259		APT-AMPICLOXA CAP	VICKMANS LABORATORIES LTD	HK-41426	No
260		BEST 250 CAP 250MG	CEUTICAL TRADING COMPANY LIMITED	HK-25561	Unknown
261		CLOXAMPI CAP	VICKMANS LABORATORIES LTD	HK-21260	No
262		CLOXAMPICIN CAP	YAT SENG TRADING CO	HK-45681	Unknown
263		KINTAMVY FOR INJ 0.75G	JINDUN PHARMA (H.K.) LIMITED	HK-56070	Unknown
264		LAMPICIN FORT CAP	BRIGHT FUTURE PHARMACEUTICALS FACTORY O/B BRIGHT FUTURE PHARMACEUTICAL LABORATORIES LIMITED	HK-37322	Unknown
265		LOXAMP CAPSULES	SWEDISH TRADING COMPANY LIMITED	HK-63700	Unknown
266		NEO A C CAP	VICKMANS LABORATORIES LTD	HK-17913	No
267		NEO BRITIN CAP 500MG	VICKMANS LABORATORIES LTD	HK-14294	No
268		NICE AMCLOX 2 CAP	VICKMANS LABORATORIES LTD	HK-26072	No
269		NORAPILIN POWDER FOR SOLUTION FOR IM/IV INJECTION 500MG	JINDUN PHARMA (H.K.) LIMITED	HK-61293	Unknown
270		PAMECIL FOR INJ 500MG	STAR MEDICAL SUPPLIES LTD	HK-34597	No
271		PAMECIL FORTE FOR SYRUP 250MG/5ML	STAR MEDICAL SUPPLIES LTD	HK-40801	No
272		PAN-AMPICILLIN FOR INJ 500MG	DCH AURIGA (HONG KONG) LIMITED - UNIVERSAL DIVISION	HK-51213	Unknown
273		REICHLIN CAP 250MG	MEDREICH FAR EAST LIMITED	HK-52945	Unknown
274		SYNPICLOX CAP	VICKMANS LABORATORIES LTD	HK-55753	No
275		UNASYN 750 FOR INJ	PFIZER CORPORATION HONG KONG LIMITED	HK-27636	Unknown
276	**MEFENAMIC ACID**	ALFOXAN CAPSULES 250MG	HEALTHCARE PHARMASCIENCE LIMITED	HK-66854	Unknown
277		ANALGIC-500 TABLETS 500MG	MEDILINE (HONG KONG) COMPANY LIMITED	HK-66661	Unknown
278		ANALMIN CAP 250MG	MEYER PHARMACEUTICALS LTD	HK-20657	Unknown
279		ANALMIN FORTE TAB 500MG	MEYER PHARMACEUTICALS LTD	HK-40133	Unknown
280		EUROTAN-F SUSP 250MG/5ML	EUROPHARM LAB CO LTD	HK-42367	No
281		EUROTAN-F TAB 500MG	EUROPHARM LAB CO LTD	HK-41547	Yes
282		FEMINAR TABLETS 500MG	WELLDONE PHARMACEUTICALS LIMITED	HK-63661	Unknown
283		FENAGESIC-500 TAB 500MG	ORIENTAL INT'L HEALTH PRODUCTS CO LTD	HK-46982	Unknown
284		FENAPON TAB 500MG	NEOCHEM PHARMACEUTICAL LABORATORIES LTD.	HK-47034	Unknown
285		GANDIN CAP 250MG	NATURAL HEALTH RESOURCES COMPANY LIMITED	HK-47771	No
286		GYNOGESIC 500 TAB 500MG	TRENTON-BOMA LTD	HK-50544	No
287		GYNOGESIC CAP 250MG	TRENTON-BOMA LTD	HK-50543	No
288		HOSTAN 250 CAP 250MG	HOVID LIMITED	HK-35625	Unknown
289		MACROMEFA 500 TAB 500MG	NATURAL HEALTH RESOURCES COMPANY LIMITED	HK-48133	No
290		MEDICAP CAP 250MG	FP HEALTHCARE LIMITED	HK-29287	Unknown
291		MEFA TAB 250MG	JEAN-MARIE PHARMACAL CO LTD	HK-01689	Yes
292		MEFAMIC TAB 250MG	NEOCHEM PHARMACEUTICAL LABORATORIES LTD.	HK-44562	Unknown
293		MEFANIC CAP 250MG	APT PHARMA LIMITED	HK-41903	Unknown
294		MEFEC 250 TAB 250MG	NATURAL HEALTH RESOURCES COMPANY LIMITED	HK-56004	No
295		MEFEMIC 500 TAB 500MG	WAI LUN TRADING CO	HK-56583	Unknown
296		MEFEN CAP 250MG	HEALTHCARE PHARMASCIENCE LIMITED	HK-38108	Unknown
297		MEFENA F.C. TAB 500MG	VAST RESOURCES PHARMACEUTICAL LTD	HK-52898	Unknown
298		MEFENAM CAPSULES 250MG	HITPHARM PHARMACEUTICAL CO LTD	HK-64262	Unknown
299		MEFENAM TABLETS 500MG	HITPHARM PHARMACEUTICAL CO LTD	HK-65305	Unknown
300		MEFENAMA CAPSULES 250MG	WELLDONE PHARMACEUTICALS LIMITED	HK-63299	Unknown
301		MEFENAMIC ACID 250 SUSP 250MG/5ML (QUALITY)	QUALITY PHARM LAB LTD	HK-25512	Unknown
302		MEFENAMIC ACID CAP 250MG	EUROPHARM LAB CO LTD	HK-27942	No
303		MEFENAMIC ACID CAP 250MG	ADVANCE PHARMACEUTICAL COMPANY LIMITED	HK-20046	Unknown
304		MEFENAMIC ACID CAP 250MG	NATIONAL PHARMACEUTICAL CO LTD	HK-15472	No
305		MEFENAMIC ACID CAP 250MG	NEOCHEM PHARMACEUTICAL LABORATORIES LTD.	HK-06351	Unknown
306		MEFENAMIC ACID CAP 250MG	VICKMANS LABORATORIES LTD	HK-50267	No
307		MEFENAMIC ACID CAP 250MG (BRIGHT FUTURE)	BRIGHT FUTURE PHARMACEUTICALS FACTORY O/B BRIGHT FUTURE PHARMACEUTICAL LABORATORIES LIMITED	HK-39771	Unknown
308		MEFENAMIC ACID CAP 250MG (JM)	JEAN-MARIE PHARMACAL CO LTD	HK-47687	No
309		MEFENAMIC ACID CAP 250MG (VIDA)	VICKMANS LABORATORIES LTD	HK-06179	No
310		MEFENAMIC ACID CAPSULES 250MG	PRUDENTLINK LIMITED	HK-65984	Unknown
311		MEFENAMIC ACID TAB 125MG	NEOCHEM PHARMACEUTICAL LABORATORIES LTD.	HK-12918	Unknown
312		MEFENAMIC ACID TAB 250MG	VICKMANS LABORATORIES LTD	HK-29271	No
313		MEFENAMIC ACID TAB 250MG	EUROPHARM LAB CO LTD	HK-27958	No
314		MEFENCID CAP 250MG	CHRISTO PHARM LTD	HK-13376	No
315		MEFENCID CAP GREY/WHITE 250MG	CHRISTO PHARM LTD	HK-30324	No
316		MEFENCID TAB 250MG	CHRISTO PHARM LTD	HK-27721	No

317		MEFENE F.C. TAB 500MG	HITPHARM PHARMACEUTICAL CO LTD	HK-48980	Unknown
318		MEFENSTAN CAP 250MG (IVORY)	NICE LABORATORIES LTD	HK-07380	Unknown
319		MEFETAB TABLETS 500MG	VAST RESOURCES PHARMACEUTICAL LTD	HK-67305	Unknown
320		MEFETON TABLETS 500MG	WELLDONE PHARMACEUTICALS LIMITED	HK-66441	Unknown
321		MEFIC CAP 250MG	LUEN CHEONG HONG LTD	HK-33003	Unknown
322		METSYN CAP 250MG	SYNCO (H.K.) LIMITED	HK-05365	No
323		METSYN CAP 250MG (BROWN/YELLOW)	VICKMANS LABORATORIES LTD	HK-31402	No
324		METSYN CAP 250MG IVORY	NICE LABORATORIES LTD	HK-19258	Unknown
325		METSYN TABLETS 250MG	NEOCHEM PHARMACEUTICAL LABORATORIES LTD.	HK-62754	Unknown
326		NAMIC CAP 250MG	ATLANTIC PHARMACEUTICAL LIMITED	HK-50264	Unknown
327		NAMIC TAB 500MG	ATLANTIC PHARMACEUTICAL LIMITED	HK-50225	Unknown
328		PAINNOX TAB 500MG	DELTAPHARM LIMITED	HK-41144	Unknown
329		PHARMA MEFENAMIC ACID TAB 250MG	JULIUS CHEN & CO (HK) LTD	HK-43896	Unknown
330		PHARMANIAGA MEFENAMIC TAB 500MG	HEALTHCARE PHARMASCIENCE LIMITED	HK-52899	Unknown
331		PONGESIC 250 TAB 250MG	HEALTHCARE PHARMASCIENCE LIMITED	HK-47701	Unknown
332		PONSGESIC CAP 250MG	UNICORN LABORATORIES O/B AMERICAN UNICORN LABORATORIES LIMITED	HK-20490	Unknown
333		PONSIS TAB 250MG	VICKMANS LABORATORIES LTD	HK-51797	No
334		PONSPAIN TABLETS 500MG	EUROPHARM LAB CO LTD	HK-63186	Yes
335		PONSTAL F.C. TAB 500MG	WINGS PHARMACEUTICAL LTD	HK-45716	No
336		PONSTAN CAP 250MG	PFIZER CORPORATION HONG KONG LIMITED	HK-06133	Unknown
337		PONSTAN FILM COATED TAB 500MG	PFIZER CORPORATION HONG KONG LIMITED	HK-08232	Unknown
338		PONSTEL TAB 500MG	JULIUS CHEN & CO (HK) LTD	HK-38157	Unknown
339		PONTACID CAP 250MG	WINGS PHARMACEUTICAL LTD	HK-46687	No
340		PONTALON TABLETS 500MG	YUNG SHIN CO LTD	HK-67356	Unknown
341		POTARLON FILM COATED TAB 500MG	YUNG SHIN CO LTD	HK-34425	Unknown
342		POTARLON TAB 250MG	YUNG SHIN CO LTD	HK-43475	Unknown
343		PRESITON TABLETS 250MG	WELLDONE PHARMACEUTICALS LIMITED	HK-66476	Unknown
344		S-MEFENAMIC ACID CAP 250MG	ADVANCE PHARMACEUTICAL COMPANY LIMITED	HK-42862	Unknown
345		SEFMIC 500 TAB 500MG	HEALTH ALLIANCE INTERNATIONAL CO LTD	HK-50373	Yes
346		SEFMIC TAB 250MG	HEALTH ALLIANCE INTERNATIONAL CO LTD	HK-45150	Yes
347		TOEEFON TAB 250MG.	VAST RESOURCES PHARMACEUTICAL LTD	HK-56387	Unknown
348		U-PONOL FORTE SUSPENSION 250MG/5ML	EUROPHARM LAB CO LTD	HK-49167	No
349		U-PONOL FORTE TAB 500MG	NEOCHEM PHARMACEUTICAL LABORATORIES LTD.	HK-28637	Unknown
350	**COTRIMOXAZOLE**	APO-SULFATRIM 400-80MG TAB	HIND WING CO LTD	HK-09295	Unknown
351		B S CO-TRIMOXAZOLE TAB	FP HEALTHCARE LIMITED	HK-34423	Unknown
352		CO-SEPTIC TAB	JEAN-MARIE PHARMACAL CO LTD	HK-43066	No
353		COBACIDE TAB	VAST RESOURCES PHARMACEUTICAL LTD	HK-59021	Unknown
354		DBL SULFAMETHOXAZOLE AND TRIMETHOPRIM CONCENTRATE FOR SOLUTION FOR INFUSION 400MG/80MG	PFIZER CORPORATION HONG KONG LIMITED	HK-65292	Unknown
355		PO-TRIM TABLETS	HEALTHCARE PHARMASCIENCE LIMITED	HK-64692	Unknown
356		RESPRIM TAB	LUEN CHEONG HONG LTD	HK-33795	Unknown
357		SEPTOL TAB	CHRISTO PHARM LTD	HK-21145	No
358		SUPRIM 480 TAB	HOVID LIMITED	HK-35654	Unknown
359		SUPRIM SUSPENSION	HOVID LIMITED	HK-36007	Unknown
360		TRIMETHOPRIM SUSP 50MG/5ML	SINO-ASIA PHARMACEUTICAL SUPPLIES LTD	HK-56426	No
361		TRIMETRIN CAP	VICKMANS LABORATORIES LTD	HK-21598	No
362		TRIMEZOLE SUSPENSION	QUALITY PHARM LAB LTD	HK-09263	Unknown
363	**IBUPROFEN**	ADVIL LIQUID CAPSULES 200MG	GLAXOSMITHKLINE CONSUMER HEALTHCARE (HONG KONG) LIMITED	HK-61573	Unknown
364		AMBUFEN 400 TAB 400MG	NATURAL HEALTH RESOURCES COMPANY LIMITED	HK-51805	Yes
365		APO-IBUPROFEN TAB 200MG	HIND WING CO LTD	HK-35198	Unknown
366		APT-IBUPROFEN TAB 200MG	APT PHARMA LIMITED	HK-43232	Unknown
367		BIFEN SUSP 100MG/5ML	TEVA PHARMACEUTICAL HONG KONG O/B TEVA PHARMACEUTICAL HONG KONG LIMITED	HK-45967	Unknown
368		BRUFEN SUSPENSION 100MG/5ML	ABBOTT LAB LTD	HK-54288	No
369		BRUFEN TAB 400MG	ABBOTT LAB LTD	HK-54601	No
370		BRUMED TAB 200MG	MEDREICH FAR EAST LIMITED	HK-54667	Unknown
371		BRUPRON TAB 200MG	MEYER PHARMACEUTICALS LTD	HK-32635	Unknown
372		BUPOGESIC 200 TAB 200MG	VICKMANS LABORATORIES LTD	HK-43233	No
373		CHEMISTS' OWN IBUPROFEN TAB 200MG	LUEN CHEONG HONG LTD	HK-60468	Unknown
374		CROWN IBUPROFEN TAB 400MG	WINGS PHARMACEUTICAL LTD	HK-60247	Yes
375		CUFENIN-200 TAB 200MG	JEAN-MARIE PHARMACAL CO LTD	HK-26009	Yes
376		DAE HWA IBUPROFEN TAB 400MG	WELL FAVOURED LTD	HK-57683	Unknown
377		DOLO-SPEDIFEN GRANULES 200MG/SACHET	ZENFIELDS (H.K.) LIMITED	HK-50442	Unknown
378		DYNA IBUPROFEN TAB 400MG	WINGS PHARMACEUTICAL LTD	HK-59885	No
379		ESPEN 200 TABLETS 200MG	JULIUS CHEN & CO (HK) LTD	HK-65844	Unknown
380		FEBRYN SUSPENSION 100MG/5ML	HEALTH ALLIANCE INTERNATIONAL CO LTD	HK-65452	No
381		GOFEN 200 CAPSULES 200MG	ZUELLIG PHARMA LTD	HK-63458	Unknown
382		GOODSENSE CHILDREN'S IBUPROFEN ORAL SUSPENSION 100MG/5ML	WILSON TRADING COMPANY LIMITED	HK-65627	Unknown
383		IBRUFEN TABLETS 400MG	HITPHARM PHARMACEUTICAL CO LTD	HK-65524	Unknown
384		IBUFEN TAB 400MG	WELL FAVOURED LTD	HK-52574	Unknown
385		IBUPEN SUSP 100MG/5ML	EUROPHARM LAB CO LTD	HK-44771	No
386		IBUPROFEN FILM COATED TABLETS 200MG	WELLDONE PHARMACEUTICALS LIMITED	HK-63300	Unknown
387		IBUPROFEN TAB 200MG	FORTUNE NATIONAL (HONG KONG) LIMITED	HK-43020	Unknown
388		IBUPROFEN TAB 200MG (SHANDONG XINHUA)	JINDUN PHARMA (H.K.) LIMITED	HK-49319	Unknown
389		IBUPROFEN TAB 200MG (VICKMANS)	VICKMANS LABORATORIES LTD	HK-35229	No
390		IBUPROFEN TAB 400MG	WELLDONE PHARMACEUTICALS LIMITED	HK-59527	Unknown
391		IBUPROFEN TAB 400MG	VAST RESOURCES PHARMACEUTICAL LTD	HK-54803	Unknown
392		IBUPROFEN TAB S C 200MG	JEAN-MARIE PHARMACAL CO LTD	HK-26090	Yes
393		IBURON SUSPENSION 20MG/ML	WINGS PHARMACEUTICAL LTD	HK-65272	No
394		IBUSPAN TAB 200MG	KAI YUEN PHARMACEUTICAL CO	HK-49327	Unknown
395		ILOFEN ORAL SUSP 100MG/5ML	BRIGHT FUTURE PHARMACEUTICALS FACTORY O/B BRIGHT FUTURE PHARMACEUTICAL LABORATORIES LIMITED	HK-56635	Unknown
396		ILOFEN ORAL SUSPENSION DROPS 40MG/ML	BRIGHT FUTURE PHARMACEUTICALS FACTORY O/B BRIGHT FUTURE PHARMACEUTICAL LABORATORIES LIMITED	HK-56874	Unknown
397		INFACALM IBUPROFEN INFANT DROPS 40MG/ML	TIANDA PHARMACEUTICALS LIMITED	HK-55058	Unknown
398		INFACALM IBUPROFEN ORAL SUSP 20MG/ML	TIANDA PHARMACEUTICALS LIMITED	HK-55059	Unknown
399		IPOGESIC 200 TAB 200MG	APT PHARMA LIMITED	HK-43234	Unknown
400		IPUFEN F.C. TAB 400MG	HITPHARM PHARMACEUTICAL CO LTD	HK-51080	Unknown
401		JEANIFEN TABLET 200MG	VICKMANS LABORATORIES LTD	HK-64159	Yes
402		JMP IBUPROFEN TAB 200MG	JEAN-MARIE PHARMACAL CO LTD	HK-54194	Yes
403		KELANG F.C. TAB 200MG	VAST RESOURCES PHARMACEUTICAL LTD	HK-54455	Unknown
404		KENJAR IBUPROFEN TABLETS 200MG	WELLDONE PHARMACEUTICALS LIMITED	HK-67022	Unknown
405		KOFENE TABLETS 200MG	HITPHARM PHARMACEUTICAL CO LTD	HK-65615	Unknown
406		KORUS IBUPROFEN TAB 200MG	LSB (HK) LIMITED	HK-60919	Unknown
407		MAXIGESIC TABLETS	DKSH HONG KONG LIMITED	HK-65649	Unknown
408		NEUTROPAIN SR CAP 300MG	US NANO FOOD AND DRUG (HK) LIMITED	HK-54138	Unknown
409		NEUTROPAIN TAB 200MG	US NANO FOOD AND DRUG (HK) LIMITED	HK-49141	Unknown
410		NUROFEN COLD & FLU TAB	RECKITT BENCKISER HONG KONG LTD	HK-40772	No
411		NUROFEN FOR CHILDREN SYRUP 100MG/5ML	RECKITT BENCKISER HONG KONG LTD	HK-58322	No
412		NUROFEN SUGAR-COATED TABLETS 200MG	RECKITT BENCKISER HONG KONG LTD	HK-64198	Yes
413		P-FEN 400 TABLETS 400MG	MEDILINE (HONG KONG) COMPANY LIMITED	HK-64678	Unknown
414		P-FEN SUSPENSION 100MG/5ML	MEDILINE (HONG KONG) COMPANY LIMITED	HK-62871	Unknown
415		PARKINS TAB 400MG	WELLDONE PHARMACEUTICALS LIMITED	HK-59526	Unknown
416		PEDEA SOLUTION FOR INJECTION 10MG/2ML	TALENT INTERNATIONAL TRADING (HK) LIMITED	HK-66722	Unknown
417		PEROFEN 200 TAB 200MG	HEALTHCARE PHARMASCIENCE LIMITED	HK-44424	Unknown
418		POTOFEN CAP 200MG	EUROPHARM LAB CO LTD	HK-55478	No
419		POTOFEN SUSPENSION (NEW FORMULA) 100MG/5ML	EUROPHARM LAB CO LTD	HK-63465	No
420		PROFEN TAB 200MG	CHRISTO PHARM LTD	HK-21299	No
421		RUPAN TAB 200MG	STAR MEDICAL SUPPLIES LTD	HK-33585	Yes
422		SCHUFEN TAB 400MG	HEALTHCARE PHARMASCIENCE LIMITED	HK-48665	Unknown
423		SLOW-IBUPROFEN TAB 300MG SUSTAIN RELEASE	VICKMANS LABORATORIES LTD	HK-40359	Yes
424		SPEDIFEN GRANULES 400MG/SACHET	ZENFIELDS (H.K.) LIMITED	HK-49714	Unknown
425		SPEDIFEN TAB 400MG	ZENFIELDS (H.K.) LIMITED	HK-53233	Unknown

426		SYN-IBUPROFEN SUSPENSION 100MG/5ML	EUROPHARM LAB CO LTD	HK-63666	No
427		SYNPROFEN TAB 200MG	SYNCO (H.K.) LIMITED	HK-24228	No
428		TAI GUK IBUPROFEN TABLETS 200MG	WELL FAVOURED LTD	HK-63935	Unknown
429		TOSPAN IBUPROFEN TABLETS 200MG	WELLDONE PHARMACEUTICALS LIMITED	HK-67023	Unknown
430		TRIFENE 200 FILM-COATED TABLETS 200MG	CNW (HK) LTD	HK-63476	Unknown
431		TRIFENE DISPERSIBLE TABLETS 200MG	CNW (HK) LTD	HK-63083	Unknown
432		TRIFENE ORAL SUSPENSION 100MG/5ML	CNW (HK) LTD	HK-62000	Unknown
433		TUOAN DISPERSIBLE TAB	TIANDA PHARMACEUTICALS LIMITED	HK-61262	Unknown
434		U-IBUPROFEN SUSPENSION 100MG/5ML	EUROPHARM LAB CO LTD	HK-63472	No
435		U-IBUPROFEN TAB 200MG	JEAN-MARIE PHARMACAL CO LTD	HK-28629	Yes
436		UNI-IBUPROFEN SUSPENSION 100MG/5ML	EUROPHARM LAB CO LTD	HK-63665	No
437		VICBROFEN ORAL SUSPENSION 100MG/5ML	EUROPHARM LAB CO LTD	HK-63664	No
438		WELANT IBUPROFEN TABLETS 200MG	WELLDONE PHARMACEUTICALS LIMITED	HK-67024	Unknown
439		WILLIPO IBUPROFEN TABLETS 200MG	WELLDONE PHARMACEUTICALS LIMITED	HK-67025	Unknown
440		ZOFEN TAB 200MG	FP HEALTHCARE LIMITED	HK-31542	Unknown
441	**PARACETAMOL**	5-WAY COUGH AND FLU CAP	UNICORN LABORATORIES O/B AMERICAN UNICORN LABORATORIES LIMITED	HK-46805	Unknown
442		ABERCROM CAP	EUROPHARM LAB CO LTD	HK-52204	No
443		ACEPHEN TAB 300MG	NICE LABORATORIES LTD	HK-38754	Unknown
444		ACET 120MG SUPP	TRENTON-BOMA LTD	HK-56375	No
445		ACETAMINOPHEN CAP 500MG	VICKMANS LABORATORIES LTD	HK-46309	No
446		ACETAMOL (B) TAB 250MG	VICKMANS LABORATORIES LTD	HK-50578	No
447		ACETAMOL SUSP 250MG/5ML PINK	EUROPHARM LAB CO LTD	HK-23964	No
448		ACETAMOL SUSP 250MG/5ML YELLOW	VICKMANS LABORATORIES LTD	HK-23965	No
449		ACETAMOL TAB 500MG	NEOCHEM PHARMACEUTICAL LABORATORIES LTD.	HK-25380	Unknown
450		ACTIVELIN CAP	VICKMANS LABORATORIES LTD	HK-55371	No
451		ADVANCE PARAMOL EXTRA TAB	ADVANCE PHARMACEUTICAL COMPANY LIMITED	HK-52766	Unknown
452		AIRACOLIN CAP	VICKMANS LABORATORIES LTD	HK-55375	No
453		ALCONASE CAP	EUROPHARM LAB CO LTD	HK-55545	No
454		AMANOLIN CAPSULE	VICKMANS LABORATORIES LTD	HK-55530	No
455		AMERICA CAP	BRIGHT FUTURE PHARMACEUTICALS FACTORY O/B BRIGHT FUTURE PHARMACEUTICAL LABORATORIES LIMITED	HK-37752	Unknown
456		ANALGISER TAB 500MG	CNW FAR EAST LIMITED	HK-26859	Unknown
457		ANAPAR TAB 500MG	MEYER PHARMACEUTICALS LTD	HK-38748	Unknown
458		ANAPHEN TAB 500MG	MEYER PHARMACEUTICALS LTD	HK-38749	Unknown
459		ANAREX TAB	VAST RESOURCES PHARMACEUTICAL LTD	HK-53566	Unknown
460		ANGENOL TAB 500MG	APT PHARMA LIMITED	HK-45065	Unknown
461		ANGTAB TAB 500MG	APT PHARMA LIMITED	HK-43034	Unknown
462		ANSPANED COLD TABLETS	WELLDONE PHARMACEUTICALS LIMITED	HK-67020	Unknown
463		ANTI-COLD JUNIOR TAB	QUALITY PHARM LAB LTD	HK-49582	Unknown
464		ANTI-COLD TAB	UNICORN LABORATORIES O/B AMERICAN UNICORN LABORATORIES LIMITED	HK-50183	Unknown
465		ANTICOL TAB	VICKMANS LABORATORIES LTD	HK-08373	No
466		ANTICOLD CAP	CHRISTO PHARM LTD	HK-21107	No
467		ANTIFLU CAP	VICKMANS LABORATORIES LTD	HK-04941	No
468		ANTIFLU FORTE CAP	VICKMANS LABORATORIES LTD	HK-22812	No
469		ANTIFLU FORTE CAP (BLUE/GREY)	SYNCO (H.K.) LIMITED	HK-64689	No
470		ANTIFLU-N FORTE CAP	EUROPHARM LAB CO LTD	HK-29425	No
471		ANTINO TABLETS	LSB (HK) LIMITED	HK-62385	Unknown
472		APAP CAPLET 500MG	SHERATON WORLDWIDE DRUGS CO LTD	HK-38851	Unknown
473		APO-TRAMADOL/ACET TAB 37.5/325MG	HIND WING CO LTD	HK-61588	Unknown
474		APT-KENAMOSE TABLETS 500MG	APT PHARMA LIMITED	HK-64439	Unknown
475		APT-PARACETAMOL 250 TAB	APT PHARMA LIMITED	HK-43033	Unknown
476		ARCOMIN CAP	VICKMANS LABORATORIES LTD	HK-54443	No
477		ARFEN 125 SUPP 125MG	STAR MEDICAL SUPPLIES LTD	HK-37213	No
478		ARFEN PLUS TAB	STAR MEDICAL SUPPLIES LTD	HK-46310	No
479		ARFEN TAB 500MG	MEDOCHEMIE (HONG KONG) LIMITED	HK-34043	Unknown
480		ARIKAN CAP	EUROPHARM LAB CO LTD	HK-55546	No
481		ARIZEN CAP	EUROPHARM LAB CO LTD	HK-55547	No
482		ARKINAS CAP	EUROPHARM LAB CO LTD	HK-55544	No
483		ASTONI CAP	KAREN LABORATORIES O/B KAREN PHARMACEUTICAL CO LTD	HK-58795	Unknown
484		AXCEL NASATAB TABLETS	KOTRA PHARMA (HONG KONG) COMPANY	HK-64468	Unknown
485		AXCEL PARACETAMOL TAB 500MG	KOTRA PHARMA (HONG KONG) COMPANY	HK-54358	Unknown
486		AXCEL PARACETAMOL-250 SUSP 250MG/5ML	KOTRA PHARMA (HONG KONG) COMPANY	HK-47844	Unknown
487		BACTERMIN CAP	VICKMANS LABORATORIES LTD	HK-54447	No
488		BEAFLU-PLUS TAB	WAI LUN TRADING CO	HK-59307	Unknown
489		BEECHAMS HOT LEMON POWDER	GLAXOSMITHKLINE CONSUMER HEALTHCARE (HONG KONG) LIMITED	HK-37100	Unknown
490		BENITAPP CAP	EUROPHARM LAB CO LTD	HK-52206	No
491		BERITON CAP	KAREN LABORATORIES O/B KAREN PHARMACEUTICAL CO LTD	HK-58798	Unknown
492		BETACAPINE CAP	KAREN LABORATORIES O/B KAREN PHARMACEUTICAL CO LTD	HK-59277	Unknown
493		BETHOLIN CAP	VICKMANS LABORATORIES LTD	HK-55532	No
494		BF-PARACETAL CAP 500MG	BRIGHT FUTURE PHARMACEUTICALS FACTORY O/B BRIGHT FUTURE PHARMACEUTICAL LABORATORIES LIMITED	HK-46137	Unknown
495		BF-PARACETAMOL ORAL SOLUTION 160MG/5ML	BRIGHT FUTURE PHARMACEUTICALS FACTORY O/B BRIGHT FUTURE PHARMACEUTICAL LABORATORIES LIMITED	HK-63929	Unknown
496		BF-PARADAC TAB 500MG	BRIGHT FUTURE PHARMACEUTICALS FACTORY O/B BRIGHT FUTURE PHARMACEUTICAL LABORATORIES LIMITED	HK-48445	Unknown
497		BIOGESIC SUSPENSION 100MG/ML (ORANGE)	UNAM CORPORATION LTD	HK-53600	Unknown
498		BIOGESIC SUSPENSION 120MG/5ML (STRAWBERRY)	UNAM CORPORATION LTD	HK-53258	Unknown
499		BIOGESIC TABLETS 500MG	UNAM CORPORATION LTD	HK-63024	Unknown
500		BISOMINIL CAP	APT PHARMA LIMITED	HK-46935	Unknown
501		BOBOMED TAB	VICKMANS LABORATORIES LTD	HK-54223	No
502		BODI COUGH & FLU TAB	UNICORN LABORATORIES O/B AMERICAN UNICORN LABORATORIES LIMITED	HK-50242	Unknown
503		BONCOL-FORTE CAP	APT PHARMA LIMITED	HK-44235	Unknown
504		BONOMED CAP	KAREN LABORATORIES O/B KAREN PHARMACEUTICAL CO LTD	HK-59363	Unknown
505		BRENDOLIN CAP	VICKMANS LABORATORIES LTD	HK-55379	No
506		BRIANOLIN CAP	VICKMANS LABORATORIES LTD	HK-55531	No
507		BROMETONE TAB	VICKMANS LABORATORIES LTD	HK-50764	No
508		BROMOCAP CAP	APT PHARMA LIMITED	HK-58533	Unknown
509		C. AMY COLD AND COUGH CAP	NEOCHEM PHARMACEUTICAL LABORATORIES LTD.	HK-54768	Unknown
510		C18 CAP	KAREN LABORATORIES O/B KAREN PHARMACEUTICAL CO LTD	HK-59365	Unknown
511		CAD-FORTE CAP	EUROPHARM LAB CO LTD	HK-52323	No
512		CALPENOL TAB 500MG	APT PHARMA LIMITED	HK-45152	Unknown
513		CAMGESIC TAB	WAI LUN TRADING CO	HK-39489	Unknown
514		CAMILOLIN CAP	VICKMANS LABORATORIES LTD	HK-55529	No
515		CAMPO SYRUP 125MG/5ML	QUALITY PHARM LAB LTD	HK-55659	Unknown
516		CANNEX TAB	EUROPHARM LAB CO LTD	HK-56262	No
517		CARBOMED CAP	KAREN LABORATORIES O/B KAREN PHARMACEUTICAL CO LTD	HK-59759	Unknown
518		CARBROSO CAP	KAREN LABORATORIES O/B KAREN PHARMACEUTICAL CO LTD	HK-54479	Unknown
519		CARECTAL TAB	UNICORN LABORATORIES O/B AMERICAN UNICORN LABORATORIES LIMITED	HK-46222	Unknown
520		CARHILL CAP	KAREN LABORATORIES O/B KAREN PHARMACEUTICAL CO LTD	HK-59361	Unknown
521		CARILOX CAP	EUROPHARM LAB CO LTD	HK-52209	No
522		CARINAS CAP	EUROPHARM LAB CO LTD	HK-52208	No
523		CARLYSO CAP	KAREN LABORATORIES O/B KAREN PHARMACEUTICAL CO LTD	HK-54477	Unknown
524		CARMOL TAB 500MG	NICE LABORATORIES LTD	HK-47561	Unknown
525		CARPARSO CAP	KAREN LABORATORIES O/B KAREN PHARMACEUTICAL CO LTD	HK-54486	Unknown
526		CELLTIC TAB	EUROPHARM LAB CO LTD	HK-61214	No
527		CETAMIN CAP	EUROPHARM LAB CO LTD	HK-49245	No
528		CHAISENTOMG COLD CAP	MEYER PHARMACEUTICALS LTD	HK-50855	Unknown
529		CHELSOLIN CAP	VICKMANS LABORATORIES LTD	HK-55528	No
530		CHEMISTS' OWN PARACETAMOL GELTABS TABLETS 500MG	LUEN CHEONG HONG LTD	HK-64393	Unknown
531		CHILDCOLD TAB	LOYAL ADVANCE LIMITED	HK-58005	Unknown
532		CHILDREN'S COLTALIN B1 TABLETS	FORTUNE PHARMACAL CO LTD	HK-65412	Unknown
533		CHILDREN'S COLTALIN COLD & FLU CHEW TAB	FORTUNE PHARMACAL CO LTD	HK-49363	Unknown

534	CHILDREN'S COLTALIN WITH VIT B1 TAB	FORTUNE PHARMACAL CO LTD	HK-09668	Unknown
535	CHILDREN'S FORTOLIN CHEWABLE TAB 120MG	FORTUNE PHARMACAL CO LTD	HK-48963	Unknown
536	CHRISTAMOL EXTRA TAB	CHRISTO PHARM LTD	HK-47795	No
537	CHRISTAMOL SUSP (O) 250MG/5ML	CHRISTO PHARM LTD	HK-45869	No
538	CHRISTAMOL SUSP FORTE, 250MG/5CC	CHRISTO PHARM LTD	HK-20450	No
539	CHRISTAMOL TAB 500MG	CHRISTO PHARM LTD	HK-35550	No
540	CHRISTAMOL(B) TAB 500MG	CHRISTO PHARM LTD	HK-46020	No
541	CHRISTAMOL(G) TAB 500MG	CHRISTO PHARM LTD	HK-46021	No
542	CHRISTAMOL(O) TAB 500MG	CHRISTO PHARM LTD	HK-46019	No
543	CHRISTAMOL-125 SUSP 125MG/5ML	CHRISTO PHARM LTD	HK-47296	No
544	CHRISTAMOL-2 (B) TAB 500MG	CHRISTO PHARM LTD	HK-47996	No
545	CHRISTAMOL-2 (Y) TAB 500MG	CHRISTO PHARM LTD	HK-49336	No
546	CHRISTAMOL-2 TAB 500MG	CHRISTO PHARM LTD	HK-45182	No
547	CHRISTAMOL-2 TAB 500MG (W)	CHRISTO PHARM LTD	HK-47590	No
548	CHRISTAMOL-300 (G) TAB 300MG	CHRISTO PHARM LTD	HK-48494	No
549	CHRISTAMOL-300 (Y) TAB 300MG	CHRISTO PHARM LTD	HK-48698	No
550	CHRISTAMOL-300 TAB (B) 300MG	CHRISTO PHARM LTD	HK-48422	No
551	CHRISTAMOL-300 TAB 300MG	CHRISTO PHARM LTD	HK-46141	No
552	CHRISTAMOL-500 CAP	CHRISTO PHARM LTD	HK-30729	No
553	CINFATOS COMPLEX ORAL SUSPENSION	REICH PHARM LIMITED	HK-52654	Unknown
554	CLARIFLU SUSTAINED RELEASE TAB	BAYER HEALTHCARE LIMITED	HK-47205	Unknown
555	CO AD F TABLET	WELL FAVOURED LTD	HK-62641	Unknown
556	CO-CHYMIN CAP	VICKMANS LABORATORIES LTD	HK-43933	No
557	CO-CODAMOL TAB	TEVA PHARMACEUTICAL HONG KONG O/B TEVA PHARMACEUTICAL HONG KONG LIMITED	HK-33414	Unknown
558	CO-FLUENZA CAP	VICKMANS LABORATORIES LTD	HK-06348	No
559	CO-FLUZA CAP	KAREN LABORATORIES O/B KAREN PHARMACEUTICAL CO LTD	HK-38820	Unknown
560	CO-SENDEN FLU & COUGH CAP	EUROPHARM LAB CO LTD	HK-55810	No
561	CO-SNEZET CAP	VICKMANS LABORATORIES LTD	HK-43934	No
562	CODACIN CAP	VICKMANS LABORATORIES LTD	HK-43070	No
563	CODACON TABLETS	WELL FAVOURED LTD	HK-63744	Unknown
564	CODANTIN TAB	LOYAL ADVANCE LIMITED	HK-36052	Unknown
565	COFACOL (NF) CAP	NEOCHEM PHARMACEUTICAL LABORATORIES LTD.	HK-53776	Unknown
566	COFENZA CAP	UNICORN LABORATORIES O/B AMERICAN UNICORN LABORATORIES LIMITED	HK-46482	Unknown
567	COFLUCOF CAP	UNICORN LABORATORIES O/B AMERICAN UNICORN LABORATORIES LIMITED	HK-46483	Unknown
568	COL-GAN TAB (NEW FORMULA)	KAREN LABORATORIES O/B KAREN PHARMACEUTICAL CO LTD	HK-37797	Unknown
569	COL-GAN TAB FOR CHILDREN	KAREN LABORATORIES O/B KAREN PHARMACEUTICAL CO LTD	HK-37796	Unknown
570	COLD & INFLUENZA CAP	KAREN LABORATORIES O/B KAREN PHARMACEUTICAL CO LTD	HK-30579	Unknown
571	COLD AND ALLERGY RELIEF TAB	SHERATON WORLDWIDE DRUGS CO LTD	HK-39860	Unknown
572	COLD BLOCKER TAB	UNICORN LABORATORIES O/B AMERICAN UNICORN LABORATORIES LIMITED	HK-50238	Unknown
573	COLD PEACE CAP	NATIONAL PHARMACEUTICAL CO LTD	HK-49492	No
574	COLD-CURER CAP	VICKMANS LABORATORIES LTD	HK-15685	No
575	COLD-Q FLU & COUGH CAP	EUROPHARM LAB CO LTD	HK-55863	No
576	COLDAFED CAP	VICKMANS LABORATORIES LTD	HK-43932	No
577	COLDARIN CAP	VICKMANS LABORATORIES LTD	HK-55290	No
578	COLDCAP CAP	CHRISTO PHARM LTD	HK-22637	No
579	COLDEC-F COLD RELIEF CAP	JEAN-MARIE PHARMACAL CO LTD	HK-28058	No
580	COLDESLIN-FORTE CAP	VICKMANS LABORATORIES LTD	HK-54518	No
581	COLDIKA FLU & COUGH CAP	EUROPHARM LAB CO LTD	HK-55862	No
582	COLDINA-S CAP	APT PHARMA LIMITED	HK-44251	Unknown
583	COLDMAX TAB	LOYAL ADVANCE LIMITED	HK-36055	Unknown
584	COLDMINLIN CAP	VICKMANS LABORATORIES LTD	HK-53959	No
585	COLDOMIN CAP	VICKMANS LABORATORIES LTD	HK-53570	No
586	COLDOTAPP-N CAP	KAREN LABORATORIES O/B KAREN PHARMACEUTICAL CO LTD	HK-59815	Unknown
587	COLDOZET CAP	VICKMANS LABORATORIES LTD	HK-43925	No
588	COLDPIX TABLET	EUROPHARM LAB CO LTD	HK-56408	No
589	COLDREX + C TAB	GLAXOSMITHKLINE CONSUMER HEALTHCARE (HONG KONG) LIMITED	HK-50258	Unknown
590	COLDSCAP CAP	NATIONAL PHARMACEUTICAL CO LTD	HK-49172	No
591	COLDSED COLD & FLU & COUGH CAP	EUROPHARM LAB CO LTD	HK-55864	No
592	COLDSEDIN CAP	EUROPHARM LAB CO LTD	HK-27946	No
593	COLDSIN TAB	LOYAL ADVANCE LIMITED	HK-36053	Unknown
594	COLDSPAN CAPSULES	KAREN LABORATORIES O/B KAREN PHARMACEUTICAL CO LTD	HK-59206	Unknown
595	COLDSTOP TAB	FRANKIN PHARMACEUTICAL LABORATORIES CO LTD	HK-47165	No
596	COLDTAB-2 TAB	CHRISTO PHARM LTD	HK-48285	No
597	COLDTOP-FORTE CAP	VICKMANS LABORATORIES LTD	HK-54521	No
598	COLDTROL CAP	NEOCHEM PHARMACEUTICAL LABORATORIES LTD.	HK-45302	Unknown
599	COLDZEP TAB	MEYER PHARMACEUTICALS LTD	HK-07880	Unknown
600	COLFALIN COLD RELIEF CAP	JEAN-MARIE PHARMACAL CO LTD	HK-49448	No
601	COLIDIN TAB	MEYER PHARMACEUTICALS LTD	HK-31316	Unknown
602	COLINIX CAP	NEOCHEM PHARMACEUTICAL LABORATORIES LTD.	HK-45378	Unknown
603	COLKOF-DAY CAPSULE	JULIUS CHEN & CO (HK) LTD	HK-62140	Unknown
604	COLTALIN B1 TABLETS	FORTUNE PHARMACAL CO LTD	HK-65325	Unknown
605	COLTALIN COUGH HOT REMEDY POWDER FOR ORAL SOLUTION	FP HEALTHCARE LIMITED	HK-66115	Unknown
606	COLTALIN DAY & NIGHT TABLETS	FORTUNE PHARMACAL CO LTD	HK-65806	Unknown
607	COLTALIN WITH VIT B1 TAB (FOR ADULT)	FORTUNE PHARMACAL CO LTD	HK-09667	Unknown
608	COLTALIN-GP EXTRA TAB	FORTUNE PHARMACAL CO LTD	HK-48961	Unknown
609	COLTALIN-GP TAB	FORTUNE PHARMACAL CO LTD	HK-44550	Unknown
610	COLTALIN-ND TAB	FORTUNE PHARMACAL CO LTD	HK-45133	Unknown
611	COLTRAN CAP	APT PHARMA LIMITED	HK-45659	Unknown
612	COMYLLONE CAP	VICKMANS LABORATORIES LTD	HK-43928	No
613	CONTROCOLD CAP	VICKMANS LABORATORIES LTD	HK-55288	No
614	CONTROMIN CAP	VICKMANS LABORATORIES LTD	HK-54445	No
615	COPENGESIC TABLETS	SYNCO (H.K.) LIMITED	HK-62230	No
616	CORICAP CAP	APT PHARMA LIMITED	HK-45619	Unknown
617	CORION CAP	NEOCHEM PHARMACEUTICAL LABORATORIES LTD.	HK-47486	Unknown
618	CORISTOP CAP	KAREN LABORATORIES O/B KAREN PHARMACEUTICAL CO LTD	HK-58796	Unknown
619	CORITAB TAB	ATLANTIC PHARMACEUTICAL LIMITED	HK-51023	Unknown
620	CORTAL(NON-ASPIRIN) CHEW TAB 80MG-CHILD.	ASPEN ASIA COMPANY LIMITED	HK-27626	Unknown
621	COSIC TABLETS	MEDILINE (HONG KONG) COMPANY LIMITED	HK-62872	Unknown
622	COTRAMINE CAP	APT PHARMA LIMITED	HK-46936	Unknown
623	COTUSSIC CAP	MEYER PHARMACEUTICALS LTD	HK-29147	Unknown
624	CPC APAP CAP 500MG	SHERATON WORLDWIDE DRUGS CO LTD	HK-40593	Unknown
625	CPC APAP TAB 500MG	SHERATON WORLDWIDE DRUGS CO LTD	HK-40594	Unknown
626	CRAMPOLI CAP	APT PHARMA LIMITED	HK-54559	Unknown
627	DALIKAN CAPSULE	EUROPHARM LAB CO LTD	HK-55570	No
628	DANATAN CAP	KAREN LABORATORIES O/B KAREN PHARMACEUTICAL CO LTD	HK-46013	Unknown
629	DARAFFIN TAB "S.C."	WINGS PHARMACEUTICAL LTD	HK-59839	No
630	DARLIN COLD CAP	QUALITY PHARM LAB LTD	HK-49474	Unknown
631	DECOLDAN CAP	VICKMANS LABORATORIES LTD	HK-55289	No
632	DECOZEP COLD & COUGH TAB	UNICORN LABORATORIES O/B AMERICAN UNICORN LABORATORIES LIMITED	HK-50270	Unknown
633	DELTA COLD & FLU TAB	UNICORN LABORATORIES O/B AMERICAN UNICORN LABORATORIES LIMITED	HK-50239	Unknown
634	DENACOL TABLET	EUROPHARM LAB CO LTD	HK-61813	No
635	DETROLI CAP	EUROPHARM LAB CO LTD	HK-55875	No
636	DEXA COLD RELIEF CAP	JEAN-MARIE PHARMACAL CO LTD	HK-28060	No
637	DHAMOL SUSP 500MG/5ML	TEVA PHARMACEUTICAL HONG KONG O/B TEVA PHARMACEUTICAL HONG KONG LIMITED	HK-34323	Unknown
638	DHAMOL SUSP PINK 250MG/5ML	TEVA PHARMACEUTICAL HONG KONG O/B TEVA PHARMACEUTICAL HONG KONG LIMITED	HK-31774	Unknown
639	DIKUTON TAB	WILCOME PHARMACEUTICAL CO LTD	HK-18549	Unknown
640	DIME-COLD JUNIOR TAB	QUALITY PHARM LAB LTD	HK-49467	Unknown

641	DIMECAP CAP	APT PHARMA LIMITED	HK-45620	Unknown
642	DR FREEMAN COUGH & COLD CAP	APT PHARMA LIMITED	HK-44613	Unknown
643	DR FREEMAN MULTI-SYMPTOM CAP	APT PHARMA LIMITED	HK-44590	Unknown
644	DR. BROWN FLU & COUGH CAP	EUROPHARM LAB CO LTD	HK-55865	No
645	DRISTAN COLD DAY & NIGHT TABLETS	PFIZER CORPORATION HONG KONG LIMITED	HK-66255	Unknown
646	DUOFLUE CHILDREN COLD TAB	ADVANCE PHARMACEUTICAL COMPANY LIMITED	HK-57285	Unknown
647	DUOFLUE COLD TAB	ADVANCE PHARMACEUTICAL COMPANY LIMITED	HK-37595	Unknown
648	DURACOLD COLD & COUGH TAB	UNICORN LABORATORIES O/B AMERICAN UNICORN LABORATORIES LIMITED	HK-50182	Unknown
649	DYNAPAR DICLOFENAC SODIUM AND PARACETAMOL TAB	EVERCARE PHARMACEUTICAL CO. LTD.	HK-59693	Unknown
650	EAST GREAT COLDCAP CAP	VICKMANS LABORATORIES LTD	HK-43069	No
651	EAST GREAT FLUCOMIN CAP	VICKMANS LABORATORIES LTD	HK-53574	No
652	EEZE COUGH & COLD TAB	UNICORN LABORATORIES O/B AMERICAN UNICORN LABORATORIES LIMITED	HK-50243	Unknown
653	ELOKWEI COLD TABLETS	WELLDONE PHARMACEUTICALS LIMITED	HK-66007	Unknown
654	ENDOPAIN II TAB 500MG	MEDIPHARMA LTD	HK-36718	Unknown
655	ENDOPAIN SUSP FOR CHILDREN 125MG/5ML	MEDIPHARMA LTD	HK-42035	Unknown
656	ENERGEX TAB 500MG	APT PHARMA LIMITED	HK-43118	Unknown
657	ENSID-ER EXTENDED RELEASE TABLET 650MG	LSB (HK) LIMITED	HK-62272	Unknown
658	ENZACOV CAP	UNICORN LABORATORIES O/B AMERICAN UNICORN LABORATORIES LIMITED	HK-46480	Unknown
659	EUROCETAMOL PLUS TAB	EUROPHARM LAB CO LTD	HK-55973	Yes
660	EUROCOLD CAP	EUROPHARM LAB CO LTD	HK-52212	No
661	EUROPAIN PLUS TAB	EUROPHARM LAB CO LTD	HK-48656	Yes
662	EUROPAIN SYRUP 125MG/5ML	EUROPHARM LAB CO LTD	HK-47233	No
663	EUROPAIN TAB 300MG (PINK COLOUR)	EUROPHARM LAB CO LTD	HK-59629	Yes
664	EXCELOLIN CAP	VICKMANS LABORATORIES LTD	HK-55378	No
665	EXTRA COLD & COUGH TAB	UNICORN LABORATORIES O/B AMERICAN UNICORN LABORATORIES LIMITED	HK-50176	Unknown
666	EXTRA FAST COLTALIN COLD & FLU TAB	FORTUNE PHARMACAL CO LTD	HK-55217	Unknown
667	EXTRA-STRENGTH PAIN RELIEF CAPLET 500MG	FORTUNE NATIONAL (HONG KONG) LIMITED	HK-42936	Unknown
668	EXTRA-STRENGTH PAIN RELIEF TAB 500MG	FORTUNE NATIONAL (HONG KONG) LIMITED	HK-42935	Unknown
669	F-ANTICOLD TAB	FRANKIN PHARMACEUTICAL LABORATORIES CO LTD	HK-47166	No
670	F-BEMILING FORTE CAP	VICKMANS LABORATORIES LTD	HK-55233	No
671	F-PATARMOL TAB	FRANKIN PHARMACEUTICAL LABORATORIES CO LTD	HK-48137	No
672	FARA TAB 500MG	HEALTHCARE PHARMASCIENCE LIMITED	HK-51236	Unknown
673	FASCOMIN CAP	VICKMANS LABORATORIES LTD	HK-53571	No
674	FAST (FLU, COLD AND COUGH) MULTI-SYMPTOM FORMULA CAP	NEOCHEM PHARMACEUTICAL LABORATORIES LTD.	HK-54769	Unknown
675	FAST FLU TAB	UNICORN LABORATORIES O/B AMERICAN UNICORN LABORATORIES LIMITED	HK-50245	Unknown
676	FAST-ACT COLD AND COUGH CAP	NEOCHEM PHARMACEUTICAL LABORATORIES LTD.	HK-54764	Unknown
677	FASTCOLIN CAP	VICKMANS LABORATORIES LTD	HK-53573	No
678	FASTLIN-FORTE CAP	VICKMANS LABORATORIES LTD	HK-54519	No
679	FATELIN COLD CAP	QUALITY PHARM LAB LTD	HK-49470	Unknown
680	FEBRICOL TAB	FP HEALTHCARE LIMITED	HK-50347	Unknown
681	FEDDAR CAP	EUROPHARM LAB CO LTD	HK-49248	No
682	FELPENOL TAB 500MG	APT PHARMA LIMITED	HK-45063	Unknown
683	FENCOLD CAP	KAREN LABORATORIES O/B KAREN PHARMACEUTICAL CO LTD	HK-58800	Unknown
684	FEVANDOL TAB 500MG	APT PHARMA LIMITED	HK-45155	Unknown
685	FIRMBO CAP	APT PHARMA LIMITED	HK-54380	Unknown
686	FLU-LAPZ CAP	APT PHARMA LIMITED	HK-44240	Unknown
687	FLU-OFF CAP	EUROPHARM LAB CO LTD	HK-27684	No
688	FLU-OFF TAB	EUROPHARM LAB CO LTD	HK-55028	No
689	FLU-ZEP TAB	EUROPHARM LAB CO LTD	HK-28086	No
690	FLU.COM CAP	UNICORN LABORATORIES O/B AMERICAN UNICORN LABORATORIES LIMITED	HK-46474	Unknown
691	FLUCOA TAB	JULIUS CHEN & CO (HK) LTD	HK-54593	Unknown
692	FLUCOL COLD RELIEF CAP	JEAN-MARIE PHARMACAL CO LTD	HK-28057	No
693	FLUCOLIEF-FORTE CAP	VICKMANS LABORATORIES LTD	HK-54517	No
694	FLUCOMED TAB	VICKMANS LABORATORIES LTD	HK-54224	No
695	FLUCOMINE CAP	MEYER PHARMACEUTICALS LTD	HK-37206	Unknown
696	FLUCOMINE EXTRA CAP	MEYER PHARMACEUTICALS LTD	HK-49019	Unknown
697	FLUCONIL CAP	APT PHARMA LIMITED	HK-45660	Unknown
698	FLUCOR DAY CAP	HOVID LIMITED	HK-55384	Unknown
699	FLUCOR NIGHT CAP	HOVID LIMITED	HK-57207	Unknown
700	FLUDANE CAP	VICKMANS LABORATORIES LTD	HK-21596	No
701	FLUGAL COLD RELIEF CAP	JEAN-MARIE PHARMACAL CO LTD	HK-47723	No
702	FLUGAL FLU & COUGH CAP	EUROPHARM LAB CO LTD	HK-55861	No
703	FLUSTAR CAP	VICKMANS LABORATORIES LTD	HK-53469	No
704	FLUSTOP CAP	NATIONAL PHARMACEUTICAL CO LTD	HK-49173	No
705	FLUTAMIN TABLETS	LSB (HK) LIMITED	HK-62134	Unknown
706	FLUZEP TAB	EUROPHARM LAB CO LTD	HK-30053	No
707	FOPPIZEP CAP	EUROPHARM LAB CO LTD	HK-49246	No
708	FORESTOMIN CAP	VICKMANS LABORATORIES LTD	HK-54414	No
709	FORTOLIN DAY & NIGHT TABLETS	FORTUNE PHARMACAL CO LTD	HK-66127	Unknown
710	FORTOLIN EXTRA TAB	FORTUNE PHARMACAL CO LTD	HK-49293	Unknown
711	FORTOLIN NITE TAB	FORTUNE PHARMACAL CO LTD	HK-50058	Unknown
712	FORTOLIN TAB 500MG	FORTUNE PHARMACAL CO LTD	HK-26505	Unknown
713	FURININ COLD CAPSULES	WELLDONE PHARMACEUTICALS LIMITED	HK-66976	Unknown
714	FUTALIN CAP	BRIGHT FUTURE PHARMACEUTICALS FACTORY O/B BRIGHT FUTURE PHARMACEUTICAL LABORATORIES LIMITED	HK-47164	Unknown
715	FUTARA CAP	BRIGHT FUTURE PHARMACEUTICALS FACTORY O/B BRIGHT FUTURE PHARMACEUTICAL LABORATORIES LIMITED	HK-37748	Unknown
716	G4 CAP	KAREN LABORATORIES O/B KAREN PHARMACEUTICAL CO LTD	HK-59362	Unknown
717	GABRICOSIN CAP	SYNCO (H.K.) LIMITED	HK-56265	No
718	GAVANCOSIN CAP	SYNCO (H.K.) LIMITED	HK-56418	No
719	GOLDEN COLD & COUGH TAB	UNICORN LABORATORIES O/B AMERICAN UNICORN LABORATORIES LIMITED	HK-50240	Unknown
720	GOLDEN MERIKA FLU & COUGH CAP	EUROPHARM LAB CO LTD	HK-55866	No
721	GRACICOSIN CAP	SYNCO (H.K.) LIMITED	HK-56320	No
722	GREAT WALL BRAND JINGZHI YINQIAO JIEDU PIAN TABLETS	MING WAH (H.K.) ENTERPRISE LIMITED	HK-67065	Unknown
723	GUAIDEX COLD TABLETS	WELLDONE PHARMACEUTICALS LIMITED	HK-66009	Unknown
724	GUARDPON CAP	APT PHARMA LIMITED	HK-54379	Unknown
725	HARDYCO TABLET	JULIUS CHEN & CO (HK) LTD	HK-61921	Unknown
726	HEALCOMIN CAP	VICKMANS LABORATORIES LTD	HK-53572	No
727	HEALCORIN CAP	VICKMANS LABORATORIES LTD	HK-53611	No
728	HEALTHEON CAP	VICKMANS LABORATORIES LTD	HK-52069	No
729	HELENA CAP	KAREN LABORATORIES O/B KAREN PHARMACEUTICAL CO LTD	HK-59274	Unknown
730	HINT'S TABLETS	EUROPHARM LAB CO LTD	HK-61812	No
731	HISTALIFE CAP	KAREN LABORATORIES O/B KAREN PHARMACEUTICAL CO LTD	HK-59271	Unknown
732	HO CHAI KUNG ANALGESIC TAB	KAREN LABORATORIES O/B KAREN PHARMACEUTICAL CO LTD	HK-01050	Unknown
733	HO CHAI KUNG TJI THUNG SAN	KAREN LABORATORIES O/B KAREN PHARMACEUTICAL CO LTD	HK-01025	Unknown
734	HOVID PARACETAMOL ORAL SUSPENSION SUGAR FREE 250MG/5ML	HOVID LIMITED	HK-67265	Unknown
735	HUNFANINE CAP	KAREN LABORATORIES O/B KAREN PHARMACEUTICAL CO LTD	HK-41480	Unknown
736	HYCEPHEN TABLET	LSB (HK) LIMITED	HK-61954	Unknown
737	ICOLOCE CAP	APT PHARMA LIMITED	HK-54382	Unknown
738	IGLOIE CAP	APT PHARMA LIMITED	HK-54558	Unknown
739	INFA COLD FLU & COUGH CAP	EUROPHARM LAB CO LTD	HK-55867	No
740	INFLU CAP	JEAN-MARIE PHARMACAL CO LTD	HK-23223	No
741	ISOMAZEP COLD TAB	MEYER PHARMACEUTICALS LTD	HK-47969	Unknown
742	JECEFARMA PARACETAMOL TABLETS 500MG	JULIUS CHEN & CO (HK) LTD	HK-62836	Unknown
743	JERICAP CAP	KAREN LABORATORIES O/B KAREN PHARMACEUTICAL CO LTD	HK-58797	Unknown
744	JMP PARACETAMOL & ORPHENADRINE CITRATE TABLETS	SYNCO (H.K.) LIMITED	HK-62231	No
745	JUCOXIN TAB	EUROPHARM LAB CO LTD	HK-49125	Yes
746	JUNAMED TAB	VICKMANS LABORATORIES LTD	HK-54222	No

747	JUSCOMIN CAP	VICKMANS LABORATORIES LTD	HK-54446	No
748	JUTORSIL TABLETS	WELLDONE PHARMACEUTICALS LIMITED	HK-62546	Unknown
749	KABOL CAP	UNICORN LABORATORIES O/B AMERICAN UNICORN LABORATORIES LIMITED	HK-46477	Unknown
750	KAISECOSIN CAP	SYNCO (H.K.) LIMITED	HK-56416	No
751	KALLERMIN CAP	KAREN LABORATORIES O/B KAREN PHARMACEUTICAL CO LTD	HK-54481	Unknown
752	KANGAROO COLD & FLU TAB	UNICORN LABORATORIES O/B AMERICAN UNICORN LABORATORIES LIMITED	HK-50244	Unknown
753	KAPLAM COLD & COUGH TAB	UNICORN LABORATORIES O/B AMERICAN UNICORN LABORATORIES LIMITED	HK-50175	Unknown
754	KARADOL EXTRA CAPLET	ADVANCE PHARMACEUTICAL COMPANY LIMITED	HK-52949	Unknown
755	KARDOSEP CAP	KAREN LABORATORIES O/B KAREN PHARMACEUTICAL CO LTD	HK-54556	Unknown
756	KARECIN CAP	KAREN LABORATORIES O/B KAREN PHARMACEUTICAL CO LTD	HK-54475	Unknown
757	KARENCODIN CAP	KAREN LABORATORIES O/B KAREN PHARMACEUTICAL CO LTD	HK-49010	Unknown
758	KARENCODIN-NF CAP	KAREN LABORATORIES O/B KAREN PHARMACEUTICAL CO LTD	HK-54480	Unknown
759	KARENCOSED CAP	KAREN LABORATORIES O/B KAREN PHARMACEUTICAL CO LTD	HK-49009	Unknown
760	KARENCOSED-NF CAP	KAREN LABORATORIES O/B KAREN PHARMACEUTICAL CO LTD	HK-54472	Unknown
761	KARETON CAP	KAREN LABORATORIES O/B KAREN PHARMACEUTICAL CO LTD	HK-54482	Unknown
762	KARETUSSOL CAP	KAREN LABORATORIES O/B KAREN PHARMACEUTICAL CO LTD	HK-54555	Unknown
763	KARFLUZA CAP	KAREN LABORATORIES O/B KAREN PHARMACEUTICAL CO LTD	HK-54474	Unknown
764	KARINCO CAP	KAREN LABORATORIES O/B KAREN PHARMACEUTICAL CO LTD	HK-54478	Unknown
765	KARNIZEP CAP	KAREN LABORATORIES O/B KAREN PHARMACEUTICAL CO LTD	HK-59276	Unknown
766	KARPHENIMIN CAP	KAREN LABORATORIES O/B KAREN PHARMACEUTICAL CO LTD	HK-54554	Unknown
767	KEEDEX CAP	EUROPHARM LAB CO LTD	HK-52322	No
768	KELANRIN COLD TABLETS	WELLDONE PHARMACEUTICALS LIMITED	HK-67021	Unknown
769	KENAMOSE TAB 500MG	APT PHARMA LIMITED	HK-43119	Unknown
770	KENTON CAP	EUROPHARM LAB CO LTD	HK-55568	No
771	KERENCOSIN CAP	SYNCO (H.K.) LIMITED	HK-56417	No
772	KIDFLU TAB	LOYAL ADVANCE LIMITED	HK-58004	Unknown
773	KILL-PAIN TAB	UNICORN LABORATORIES O/B AMERICAN UNICORN LABORATORIES LIMITED	HK-46220	Unknown
774	KIN-COLD TABLET	MEYER PHARMACEUTICALS LTD	HK-38005	Unknown
775	KINAX TAB	EUROPHARM LAB CO LTD	HK-56264	No
776	KING'S CAP	VICKMANS LABORATORIES LTD	HK-55377	No
777	KNOCKFLU CAP	APT PHARMA LIMITED	HK-46937	Unknown
778	KO-FLU CAP	NATIONAL PHARMACEUTICAL CO LTD	HK-49493	No
779	KO-FLU CAP (GREEN/WHITE)	NATIONAL PHARMACEUTICAL CO LTD	HK-12646	No
780	KOFLUZA CAP	UNICORN LABORATORIES O/B AMERICAN UNICORN LABORATORIES LIMITED	HK-46475	Unknown
781	KOLDAREX CAP	KAREN LABORATORIES O/B KAREN PHARMACEUTICAL CO LTD	HK-54487	Unknown
782	KOLDAXIN CAP	KAREN LABORATORIES O/B KAREN PHARMACEUTICAL CO LTD	HK-54484	Unknown
783	KONATON TABLETS 500MG	HITPHARM PHARMACEUTICAL CO LTD	HK-64204	Unknown
784	KOOLNOX CAP	DELTAPHARM LIMITED	HK-41146	Unknown
785	KOVZA CAP	UNICORN LABORATORIES O/B AMERICAN UNICORN LABORATORIES LIMITED	HK-46481	Unknown
786	LAINACOSIN CAP	SYNCO (H.K.) LIMITED	HK-56319	No
787	LECON-FORTE CAP	VICKMANS LABORATORIES LTD	HK-56501	No
788	LEMONIN POWDER	UNICORN LABORATORIES O/B AMERICAN UNICORN LABORATORIES LIMITED	HK-09662	Unknown
789	LEMSIP COLD & FLU LEMON SACHETS	RECKITT BENCKISER HONG KONG LTD	HK-51283	Yes
790	LEMSIP COLD + FLU MAX SACHETS 1000MG	RECKITT BENCKISER HONG KONG LTD	HK-52272	No
791	LEMSIP MAX COLD & FLU CAP	RECKITT BENCKISER HONG KONG LTD	HK-52554	No
792	LEOCOMIN CAP	VICKMANS LABORATORIES LTD	HK-54448	No
793	LEVOPHANE COLD TAB	MEYER PHARMACEUTICALS LTD	HK-41554	Unknown
794	LEVOPHANE EXTRA TAB	MEYER PHARMACEUTICALS LTD	HK-48825	Unknown
795	LINXER COLD TABLETS	WELLDONE PHARMACEUTICALS LIMITED	HK-66004	Unknown
796	LIVETON-FORTE CAP	VICKMANS LABORATORIES LTD	HK-54407	No
797	LOGICIN FLU STRENGTH DAY & NIGHT TAB	ASPEN ASIA COMPANY LIMITED	HK-43955	Unknown
798	LOSIVEN CAP	EUROPHARM LAB CO LTD	HK-55567	No
799	LOTRIX DAY & NIGHT TAB	EUROPHARM LAB CO LTD	HK-48883	Yes
800	LOYAL COLDCOM TAB	LOYAL ADVANCE LIMITED	HK-36056	Unknown
801	LYDONCOSIN CAP	SYNCO (H.K.) LIMITED	HK-56322	No
802	LYHEXINE CAP	MEYER PHARMACEUTICALS LTD	HK-37207	Unknown
803	LYSOCAPINE CAP	KAREN LABORATORIES O/B KAREN PHARMACEUTICAL CO LTD	HK-49014	Unknown
804	LYSOCAPINE-NF CAP	KAREN LABORATORIES O/B KAREN PHARMACEUTICAL CO LTD	HK-54476	Unknown
805	LYSOMOL CAP	MEYER PHARMACEUTICALS LTD	HK-35606	Unknown
806	MACHONIC CAP	EUROPHARM LAB CO LTD	HK-55571	No
807	MACROPOL TAB 500MG	NATURAL HEALTH RESOURCES COMPANY LIMITED	HK-50867	Yes
808	MADAME PEARL'S MP-PARACETAMOL SYRUP 125MG/5ML	LUXEMBOURG MEDICINE CO LTD	HK-63908	No
809	MAGNESI CAP	APT PHARMA LIMITED	HK-54383	Unknown
810	MAIRICOSIN CAP	SYNCO (H.K.) LIMITED	HK-56318	No
811	MANDAR COLD CAP	QUALITY PHARM LAB LTD	HK-49473	Unknown
812	MANGA TABLETS	LEAMYK INVESTMENT LIMITED	HK-65487	Unknown
813	MANLIN FLU & COUGH CAP	EUROPHARM LAB CO LTD	HK-56649	No
814	MANNINGS PAIN RELIEF TAB 500MG	ADVANCE PHARMACEUTICAL COMPANY LIMITED	HK-59055	Unknown
815	MANOSEP CAP	KAREN LABORATORIES O/B KAREN PHARMACEUTICAL CO LTD	HK-59364	Unknown
816	MANTA COLD CAP	MEYER PHARMACEUTICALS LTD	HK-49291	Unknown
817	MAPLE LEAF COLD & FLU TAB	UNICORN LABORATORIES O/B AMERICAN UNICORN LABORATORIES LIMITED	HK-50241	Unknown
818	MARYON CAP	EUROPHARM LAB CO LTD	HK-55573	No
819	MASSACTY CAP	EUROPHARM LAB CO LTD	HK-55548	No
820	MAX COLTALIN COUGH HOT REMEDY POWDER FOR ORAL SOLUTION	FP HEALTHCARE LIMITED	HK-65977	Unknown
821	MAXICLEAR COLD & FLU RELIEF TABLETS	DKSH HONG KONG LIMITED	HK-64742	Unknown
822	MAXICOLD TAB	EUROPHARM LAB CO LTD	HK-48879	No
823	MAXIGESIC TABLETS	DKSH HONG KONG LIMITED	HK-65649	Unknown
824	MCXY-COLD TABLETS	MEDILINE (HONG KONG) COMPANY LIMITED	HK-64720	Unknown
825	MECOLD (FORTE) CAP	MEYER PHARMACEUTICALS LTD	HK-07883	Unknown
826	MECOLD (SCT) RED TAB	MEYER PHARMACEUTICALS LTD	HK-07882	Unknown
827	MECOLD WAFER PLUS (IMPROVED FORMULA) TAB	MEYER PHARMACEUTICALS LTD	HK-59194	Unknown
828	MECOSTOP CAP	BRIGHT FUTURE PHARMACEUTICALS FACTORY O/B BRIGHT FUTURE PHARMACEUTICAL LABORATORIES LIMITED	HK-37749	Unknown
829	MEGA POWER COLD & FLU TAB	UNICORN LABORATORIES O/B AMERICAN UNICORN LABORATORIES LIMITED	HK-50178	Unknown
830	MENTA CAP	MEYER PHARMACEUTICALS LTD	HK-32868	Unknown
831	MERACET CAP	MEYER PHARMACEUTICALS LTD	HK-32867	Unknown
832	MERCENA CAP	APT PHARMA LIMITED	HK-54381	Unknown
833	MERICOL COLD FLU & COUGH CAP	EUROPHARM LAB CO LTD	HK-55868	No
834	MERIKA COLD RELIEF CAP	JEAN-MARIE PHARMACAL CO LTD	HK-28059	No
835	MERIZEP CAP	EUROPHARM LAB CO LTD	HK-52257	No
836	MEYER-COL EXTRA CAP	MEYER PHARMACEUTICALS LTD	HK-48829	Unknown
837	MIGAPHEN CAP	JULIUS CHEN & CO (HK) LTD	HK-44850	Unknown
838	MILANCOSIN CAP	SYNCO (H.K.) LIMITED	HK-56321	No
839	MYODRINE TAB	DELTAPHARM LIMITED	HK-44887	Unknown
840	MYOFLEX TAB	LEAMYK INVESTMENT LIMITED	HK-55766	Unknown
841	MYOPAS TABLETS	WAI LUN TRADING CO	HK-67164	Unknown
842	MYOSIC TABLET	ATLANTIC PHARMACEUTICAL LIMITED	HK-62007	Unknown
843	N2012 CAP	KAREN LABORATORIES O/B KAREN PHARMACEUTICAL CO LTD	HK-59275	Unknown
844	NAPA TAB 500MG	WILCOME PHARMACEUTICAL CO LTD	HK-18542	Unknown
845	NASOCOL CAP	MEYER PHARMACEUTICALS LTD	HK-38542	Unknown
846	NATIONCOLD CAP	NATIONAL PHARMACEUTICAL CO LTD	HK-49491	No
847	NAUTICON TABLET	EUROPHARM LAB CO LTD	HK-56410	No
848	NECTICAN CAP	EUROPHARM LAB CO LTD	HK-55566	No
849	NEOCOD TAB	LOYAL ADVANCE LIMITED	HK-36054	Unknown
850	NEOSED COUGH TAB	ADVANCE PHARMACEUTICAL COMPANY LIMITED	HK-47002	Unknown
851	NEOSED TAB	ADVANCE PHARMACEUTICAL COMPANY LIMITED	HK-11964	Unknown
852	NEOSOCOL CAP	NEOCHEM PHARMACEUTICAL LABORATORIES LTD.	HK-45452	Unknown

853	NEOZEP COLD TAB	UNAM CORPORATION LTD	HK-06539	Unknown
854	NEOZYME TAB	EUROPHARM LAB CO LTD	HK-49126	Yes
855	NEW COLD STOP FLU & COUGH CAP	EUROPHARM LAB CO LTD	HK-55806	No
856	NEW-EUROGESIC ORAL SUSPENSION 120MG/5ML (ORANGE)	EUROPHARM LAB CO LTD	HK-64272	No
857	NEWZEP CAP	EUROPHARM LAB CO LTD	HK-52325	No
858	NICE-COLD CAP	NEOCHEM PHARMACEUTICAL LABORATORIES LTD.	HK-52749	Unknown
859	NICHOCOSIN CAP	SYNCO (H.K.) LIMITED	HK-56415	No
860	NILDIZZ COLD TAB	EUROPHARM LAB CO LTD	HK-48658	Yes
861	NOLANCOSIN CAP	SYNCO (H.K.) LIMITED	HK-56419	No
862	NON-ASPIRIN PAIN RELIEF TAB 325MG	FORTUNE NATIONAL (HONG KONG) LIMITED	HK-42937	Unknown
863	NORGESIC TAB	INOVA PHARMACEUTICALS (HONG KONG) LIMITED	HK-19321	Unknown
864	NORGETEX TAB	DKSH HONG KONG LIMITED	HK-60498	Unknown
865	NORIKA FLU & COUGH CAP	EUROPHARM LAB CO LTD	HK-55807	No
866	NORMET TAB	VICKMANS LABORATORIES LTD	HK-50462	No
867	NOSCAPECT CAP	KAREN LABORATORIES O/B KAREN PHARMACEUTICAL CO LTD	HK-49013	Unknown
868	NOSCAPECT-NF CAP	KAREN LABORATORIES O/B KAREN PHARMACEUTICAL CO LTD	HK-54485	Unknown
869	NOSCOFED CAP	KAREN LABORATORIES O/B KAREN PHARMACEUTICAL CO LTD	HK-49011	Unknown
870	NOSEPINE CAP	KAREN LABORATORIES O/B KAREN PHARMACEUTICAL CO LTD	HK-59272	Unknown
871	NOSTRAMIN CAP	MEYER PHARMACEUTICALS LTD	HK-38543	Unknown
872	NUSAFE FLU & COUGH CAP	EUROPHARM LAB CO LTD	HK-55808	No
873	ONILENE TABLETS	WELLDONE PHARMACEUTICALS LIMITED	HK-62545	Unknown
874	ONWARD CAP	VICKMANS LABORATORIES LTD	HK-52071	No
875	OPTICOLD TAB	EUROPHARM LAB CO LTD	HK-48878	No
876	ORNADINE TABLET	VAST RESOURCES PHARMACEUTICAL LTD	HK-60792	Unknown
877	OTARAX TAB	EUROPHARM LAB CO LTD	HK-56173	No
878	PACIMOL TABLETS 500MG	HONG KONG MEDICAL SUPPLIES LTD	HK-65917	No
879	PAFFEICO TAB	BRIGHT FUTURE PHARMACEUTICALS FACTORY O/B BRIGHT FUTURE PHARMACEUTICAL LABORATORIES LIMITED	HK-57897	Unknown
880	PAINGOLIN CAP	VICKMANS LABORATORIES LTD	HK-55370	No
881	PAINLESS TAB	YUNG SHIN CO LTD	HK-34417	Unknown
882	PAMIDOL TAB 500MG	NEOCHEM PHARMACEUTICAL LABORATORIES LTD.	HK-43103	Unknown
883	PAMOL FORTE TAB	VICKMANS LABORATORIES LTD	HK-51732	No
884	PANADEINE TAB	SANOFI HONG KONG LIMITED	HK-02261	Unknown
885	PANADOL ACTIFAST SOLUBLE TAB 500MG	GLAXOSMITHKLINE CONSUMER HEALTHCARE (HONG KONG) LIMITED	HK-49789	Unknown
886	PANADOL ACTIFAST TAB 500MG	GLAXOSMITHKLINE CONSUMER HEALTHCARE (HONG KONG) LIMITED	HK-49376	Unknown
887	PANADOL CAPLET 500MG	GLAXOSMITHKLINE CONSUMER HEALTHCARE (HONG KONG) LIMITED	HK-53362	Unknown
888	PANADOL CLASSIC TAB 500MG	GLAXOSMITHKLINE CONSUMER HEALTHCARE (HONG KONG) LIMITED	HK-40505	Unknown
889	PANADOL COLD & COUGH TAB	GLAXOSMITHKLINE CONSUMER HEALTHCARE (HONG KONG) LIMITED	HK-53319	Unknown
890	PANADOL COLD & FLU (NEW FORMULATION) TAB	GLAXOSMITHKLINE CONSUMER HEALTHCARE (HONG KONG) LIMITED	HK-50505	Unknown
891	PANADOL COLD & FLU DAY & NIGHT CAPLET	GLAXOSMITHKLINE CONSUMER HEALTHCARE (HONG KONG) LIMITED	HK-61522	Unknown
892	PANADOL COLD & FLU DAY TAB	GLAXOSMITHKLINE CONSUMER HEALTHCARE (HONG KONG) LIMITED	HK-51085	Unknown
893	PANADOL COLD & FLU EXTRA HOT REMEDY - BERRY FRUITS	GLAXOSMITHKLINE CONSUMER HEALTHCARE (HONG KONG) LIMITED	HK-59581	Unknown
894	PANADOL COLD & FLU EXTRA HOT REMEDY POWDER-LEMON	GLAXOSMITHKLINE CONSUMER HEALTHCARE (HONG KONG) LIMITED	HK-53568	Unknown
895	PANADOL COLD & FLU HOT REMEDY POWDER	GLAXOSMITHKLINE CONSUMER HEALTHCARE (HONG KONG) LIMITED	HK-43594	Unknown
896	PANADOL COLD & FLU NASAL CAPLET	GLAXOSMITHKLINE CONSUMER HEALTHCARE (HONG KONG) LIMITED	HK-55615	Unknown
897	PANADOL COLD&FLU H.REMEDY PDR-BLACKCURR.	GLAXOSMITHKLINE CONSUMER HEALTHCARE (HONG KONG) LIMITED	HK-50933	Unknown
898	PANADOL COLD&FLU H.REMEDY PDR-LEMON/HONEY	GLAXOSMITHKLINE CONSUMER HEALTHCARE (HONG KONG) LIMITED	HK-50932	Unknown
899	PANADOL EXTEND TAB 665MG	GLAXOSMITHKLINE CONSUMER HEALTHCARE (HONG KONG) LIMITED	HK-51316	Unknown
900	PANADOL EXTRA ADVANCE CAPLET	GLAXOSMITHKLINE CONSUMER HEALTHCARE (HONG KONG) LIMITED	HK-61509	Unknown
901	PANADOL EXTRA SOLUBLE TAB	GLAXOSMITHKLINE CONSUMER HEALTHCARE (HONG KONG) LIMITED	HK-40539	Unknown
902	PANADOL FOR CHILDREN TAB CHEWABLE 120MG	GLAXOSMITHKLINE CONSUMER HEALTHCARE (HONG KONG) LIMITED	HK-31209	Unknown
903	PANADOL INFANT DROPS 100MG/ML (ORANGE/VANILLA)	GLAXOSMITHKLINE CONSUMER HEALTHCARE (HONG KONG) LIMITED	HK-52933	Unknown
904	PANADOL JOINT EXTENDED RELEASE CAPLET 665MG	GLAXOSMITHKLINE CONSUMER HEALTHCARE (HONG KONG) LIMITED	HK-59436	Unknown
905	PANADOL LONG LASTING TAB 665 MG	GLAXOSMITHKLINE CONSUMER HEALTHCARE (HONG KONG) LIMITED	HK-51314	Unknown
906	PANADOL MENSTRUAL CAPLET	GLAXOSMITHKLINE CONSUMER HEALTHCARE (HONG KONG) LIMITED	HK-53827	Unknown
907	PANADOL MINI CAP 500MG	GLAXOSMITHKLINE CONSUMER HEALTHCARE (HONG KONG) LIMITED	HK-53813	Unknown
908	PANADOL ONE TAB 1G	GLAXOSMITHKLINE CONSUMER HEALTHCARE (HONG KONG) LIMITED	HK-51472	Unknown
909	PANADOL SUPP 125MG	GLAXOSMITHKLINE CONSUMER HEALTHCARE (HONG KONG) LIMITED	HK-27104	Unknown
910	PANADOL SUSPENSION 120MG/5ML	GLAXOSMITHKLINE CONSUMER HEALTHCARE (HONG KONG) LIMITED	HK-52694	Unknown
911	PANADOL TAB 500MG	GLAXOSMITHKLINE CONSUMER HEALTHCARE (HONG KONG) LIMITED	HK-02280	Unknown
912	PANADOL ULTRA TAB	GLAXOSMITHKLINE CONSUMER HEALTHCARE (HONG KONG) LIMITED	HK-53555	Unknown
913	PANAMOL TAB 500MG	WAI LUN TRADING CO	HK-61273	Unknown
914	PANASIN TAB	VICKMANS LABORATORIES LTD	HK-50514	No
915	PANATON TABLETS 500MG	JULIUS CHEN & CO (HK) LTD	HK-66971	Unknown
916	PANCOL CAP	SYNCO (H.K.) LIMITED	HK-56323	No
917	PANOMORE TABLETS 500MG	JULIUS CHEN & CO (HK) LTD	HK-66972	Unknown
918	PANON TABLETS 500MG	WINGS PHARMACEUTICAL LTD	HK-61928	No
919	PANOZ-COL CAP	APT PHARMA LIMITED	HK-44237	Unknown
920	PARA-G TABLETS 500MG	MEDILINE (HONG KONG) COMPANY LIMITED	HK-64919	Unknown
921	PARACEFED-NF CAP	KAREN LABORATORIES O/B KAREN PHARMACEUTICAL CO LTD	HK-54471	Unknown
922	PARACET CAP 500MG	NEOCHEM PHARMACEUTICAL LABORATORIES LTD.	HK-48843	Unknown
923	PARACETAMOL	UNICO & CO	HK-64610	Unknown
924	PARACETAMOL	SUNRISE TRADING CO	HK-49098	Unknown
925	PARACETAMOL & ORPHENADRINE CITRATE TAB	SYNCO (H.K.) LIMITED	HK-64729	No
926	PARACETAMOL B TAB 500MG	NEOCHEM PHARMACEUTICAL LABORATORIES LTD.	HK-35464	Unknown
927	PARACETAMOL B. BRAUN SOLUTION FOR INFUSION 1000MG/100ML	B. BRAUN MEDICAL (HK) LTD	HK-64407	No
928	PARACETAMOL BP TAB 500MG	ASIA CHEM IND LTD	HK-08800	Unknown
929	PARACETAMOL CAP 500MG	NEOCHEM PHARMACEUTICAL LABORATORIES LTD.	HK-16308	Unknown
930	PARACETAMOL CAP 500MG	VICKMANS LABORATORIES LTD	HK-11490	No
931	PARACETAMOL CINFA TABLETS 1G	REICH PHARM LIMITED	HK-63023	Unknown
932	PARACETAMOL CINFA TABLETS 650MG	REICH PHARM LIMITED	HK-63129	Unknown
933	PARACETAMOL ELIXIR 120MG/5ML (BELL)	LINKLANDS TRADING COMPANY	HK-39020	Unknown
934	PARACETAMOL FORT SUSP 250MG/5ML (B.F.)	BRIGHT FUTURE PHARMACEUTICALS FACTORY O/B BRIGHT FUTURE PHARMACEUTICAL LABORATORIES LIMITED	HK-49046	Unknown
935	PARACETAMOL FU YUAN TABLETS 500MG	WELLDONE PHARMACEUTICALS LIMITED	HK-67331	Unknown
936	PARACETAMOL HUAWLIN TABLETS 500MG	WELLDONE PHARMACEUTICALS LIMITED	HK-67307	Unknown

937	PARACETAMOL KABI SOLUTION FOR INFUSION 1000MG/100ML	FRESENIUS KABI HONG KONG LIMITED	HK-66352	Unknown
938	PARACETAMOL KABI SOLUTION FOR INFUSION 1000MG/100ML (FREEFLEX BAG)	FRESENIUS KABI HONG KONG LIMITED	HK-66353	Unknown
939	PARACETAMOL ORAL SUSP 250MG/5ML	NEOCHEM PHARMACEUTICAL LABORATORIES LTD.	HK-11454	Unknown
940	PARACETAMOL ORAL SUSPENSION 250MG/5ML	BRIGHT FUTURE PHARMACEUTICALS FACTORY O/B BRIGHT FUTURE PHARMACEUTICAL LABORATORIES LIMITED	HK-64351	Unknown
941	PARACETAMOL ORAL SUSPENSION 250MG/5ML	SINO-ASIA PHARMACEUTICAL SUPPLIES LTD	HK-61155	No
942	PARACETAMOL ORAL SUSPENSION 250MG/5ML	PRUDENTLINK LIMITED	HK-65505	Unknown
943	PARACETAMOL SUSP 125MG/5ML	MEDIPHARMA LTD	HK-46319	Unknown
944	PARACETAMOL SUSP 250MG/5ML	EUROPHARM LAB CO LTD	HK-27401	No
945	PARACETAMOL SUSP 250MG/5ML	ADVANCE PHARMACEUTICAL COMPANY LIMITED	HK-42982	Unknown
946	PARACETAMOL SUSPENSION 250MG/5ML	EUROPHARM LAB CO LTD	HK-53451	No
947	PARACETAMOL SYRUP 250MG/5ML	JEAN-MARIE PHARMACAL CO LTD	HK-19450	Yes
948	PARACETAMOL TAB 500MG	DELTAPHARM LIMITED	HK-45099	Unknown
949	PARACETAMOL TAB 500MG	UNICORN LABORATORIES O/B AMERICAN UNICORN LABORATORIES LIMITED	HK-46660	Unknown
950	PARACETAMOL TAB 500MG	SYNCO (H.K.) LIMITED	HK-42555	No
951	PARACETAMOL TAB 500MG	TRENTON-BOMA LTD	HK-49784	No
952	PARACETAMOL TAB 500MG	JEAN-MARIE PHARMACAL CO LTD	HK-56620	No
953	PARACETAMOL TAB 500MG	STAR MEDICAL SUPPLIES LTD	HK-51731	No
954	PARACETAMOL TAB 500MG (CHINTA)	CHIN HONG & CO	HK-14628	Unknown
955	PARACETAMOL TAB 500MG (ORANGE)	NICE LABORATORIES LTD	HK-07356	Unknown
956	PARACETAMOL TAB 500MG (ORANGE)	NEOCHEM PHARMACEUTICAL LABORATORIES LTD.	HK-05450	Unknown
957	PARACETAMOL TAB 500MG (ORANGE/ORAL)	EUROPHARM LAB CO LTD	HK-27282	No
958	PARACETAMOL TAB 500MG (PLAIN)	NEOCHEM PHARMACEUTICAL LABORATORIES LTD.	HK-05452	Unknown
959	PARACETAMOL TAB 500MG (PT TEMPO SCAN P.)	SUNRAY COMPANY	HK-48760	Unknown
960	PARACETAMOL TAB 500MG (UNITED)	THE UNITED LABORATORIES LTD	HK-44242	Unknown
961	PARACETAMOL TAB 500MG (VIDA)	VICKMANS LABORATORIES LTD	HK-09456	No
962	PARACETAMOL TAB 500MG (WHITE/ROUND)	EUROPHARM LAB CO LTD	HK-27283	No
963	PARACETAMOL TAB 500MG BLUE	MEDIPHARMA LTD	HK-34108	Unknown
964	PARACETAMOL TAB 500MG GREEN	JEAN-MARIE PHARMACAL CO LTD	HK-23085	No
965	PARACETAMOL TAB 500MG WHITE (VIDA)	VICKMANS LABORATORIES LTD	HK-21361	No
966	PARACETAMOL TABLETS 500MG	PRUDENTLINK LIMITED	HK-65511	Unknown
967	PARACETAMOL TABLETS 500MG (WHITE)	APT PHARMA LIMITED	HK-62848	Unknown
968	PARACETAMOL TABLETS BP 500MG	JULIUS CHEN & CO (HK) LTD	HK-66818	Unknown
969	PARACETAMOL-AFT SOLUTION FOR INFUSION 1G/100ML	HIND WING CO LTD	HK-64803	Unknown
970	PARACODONE TAB	BRIGHT FUTURE PHARMACEUTICALS FACTORY O/B BRIGHT FUTURE PHARMACEUTICAL LABORATORIES LIMITED	HK-55815	Unknown
971	PARAFLUZED CAP	KAREN LABORATORIES O/B KAREN PHARMACEUTICAL CO LTD	HK-49015	Unknown
972	PARAFLUZED-NF CAP	KAREN LABORATORIES O/B KAREN PHARMACEUTICAL CO LTD	HK-54483	Unknown
973	PARAGESIC TAB	VICKMANS LABORATORIES LTD	HK-50513	No
974	PARAGRAM CAP 500MG	VICKMANS LABORATORIES LTD	HK-50613	No
975	PARAMOL 250 SYRUP	QUALITY PHARM LAB LTD	HK-09250	Unknown
976	PARAMOL FORTE TAB	VICKMANS LABORATORIES LTD	HK-43448	No
977	PARAMOL SUSPENSION 250MG/5ML	ADVANCE PHARMACEUTICAL COMPANY LIMITED	HK-42981	Unknown
978	PARAMOL TAB 500MG	ADVANCE PHARMACEUTICAL COMPANY LIMITED	HK-09193	Unknown
979	PARAMOLAN ORAL SOLUTION 120MG/5ML	CNW (HK) LTD	HK-65117	Unknown
980	PARAMOLAN SUPPOSITORIES 125MG	CNW (HK) LTD	HK-62167	Unknown
981	PARAMOLAN TABLETS 500MG	CNW (HK) LTD	HK-61939	Unknown
982	PARATAB TAB 500MG	PRIMAL CHEMICAL CO LTD	HK-47388	Unknown
983	PARAWAY TAB 500MG	SYNCO (H.K.) LIMITED	HK-50518	No
984	PARAZYME CAP	VICKMANS LABORATORIES LTD	HK-55372	No
985	PARCEMOL PLAIN TAB 300MG	NEOCHEM PHARMACEUTICAL LABORATORIES LTD.	HK-31745	Unknown
986	PARCEMOL SYRUP	MARCHING PHARMACEUTICAL LIMITED	HK-04932	No
987	PARCEMOL SYRUP FORTE 250MG/5ML (RED)	MARCHING PHARMACEUTICAL LIMITED	HK-25552	Yes
988	PARCOTEN TAB	DCH AURIGA (HONG KONG) LIMITED - UNIVERSAL DIVISION	HK-41616	Unknown
989	PARMOL 120 SYRUP 120MG/5ML	HOVID LIMITED	HK-35911	Unknown
990	PARNAC TAB	MEYER PHARMACEUTICALS LTD	HK-39332	Unknown
991	PAROMINE ORAL SOLUTION	BRIGHT FUTURE PHARMACEUTICALS FACTORY O/B BRIGHT FUTURE PHARMACEUTICAL LABORATORIES LIMITED	HK-53197	Unknown
992	PARTAMOL TAB 500MG	MEYER PHARMACEUTICALS LTD	HK-08172	Unknown
993	PENISUL CAP	EUROPHARM LAB CO LTD	HK-52201	No
994	PHARMACIN CAP 500MG	UNICORN LABORATORIES O/B AMERICAN UNICORN LABORATORIES LIMITED	HK-31680	Unknown
995	PHARMAGRIP CAP	REICH PHARM LIMITED	HK-53389	Unknown
996	PHARMAGRIP POWDER FOR ORAL SUSPENSION	REICH PHARM LIMITED	HK-53365	Unknown
997	PHARMANIAGA PARACETAMOL TABLET 650MG	HEALTHCARE PHARMASCIENCE LIMITED	HK-63920	Unknown
998	PHENTAMOL TAB	NATIONAL PHARMACEUTICAL CO LTD	HK-48584	No
999	PINOCINE ANALGESIC TAB 500MG	UNICORN LABORATORIES O/B AMERICAN UNICORN LABORATORIES LIMITED	HK-35518	Unknown
1000	PLABLIC CAP	EUROPHARM LAB CO LTD	HK-52202	No
1001	PLAVIDOX CAP	EUROPHARM LAB CO LTD	HK-52210	No
1002	PO ON LING POWDER 500MG	NATIONAL PHARMACEUTICAL CO LTD	HK-54183	No
1003	POCETMOL FORTE TAB	SYNCO (H.K.) LIMITED	HK-56511	No
1004	POCIPILL COLD TABLETS	WELLDONE PHARMACEUTICALS LIMITED	HK-66008	Unknown
1005	POLARMIN CAP	VICKMANS LABORATORIES LTD	HK-54444	No
1006	POLYME TAB	EUROPHARM LAB CO LTD	HK-49127	Yes
1007	PONTAMOL TAB	MEYER PHARMACEUTICALS LTD	HK-41208	Unknown
1008	POONS MOL-AC EXTRA CAPLET	ADVANCE PHARMACEUTICAL COMPANY LIMITED	HK-52948	Unknown
1009	PORMUS TAB	HEALTH ALLIANCE INTERNATIONAL CO LTD	HK-53672	No
1010	PORO SUPP 125MG	YUNG SHIN CO LTD	HK-57301	Unknown
1011	PORO TAB 500MG	YUNG SHIN CO LTD	HK-43508	Unknown
1012	POURKE CAP	APT PHARMA LIMITED	HK-54378	Unknown
1013	PRITAMOL CAPSULES 500MG	VAST RESOURCES PHARMACEUTICAL LTD	HK-66842	Unknown
1014	PRITAMOL TAB 500MG	LEAMYK INVESTMENT LIMITED	HK-56805	Unknown
1015	PROFESIN COLD CAP	NEOCHEM PHARMACEUTICAL LABORATORIES LTD.	HK-47580	Unknown
1016	PROFEXIN TABLET	EUROPHARM LAB CO LTD	HK-56409	No
1017	PROGESIC TAB 500MG	FP HEALTHCARE LIMITED	HK-34491	Unknown
1018	PROGESIC-250 SUSP 250MG	FP HEALTHCARE LIMITED	HK-35272	Unknown
1019	PROMOL TAB 500MG	MEDREICH FAR EAST LIMITED	HK-54596	Unknown
1020	PROSCO CAP	APT PHARMA LIMITED	HK-44238	Unknown
1021	PROTAPPZ CAP	APT PHARMA LIMITED	HK-44236	Unknown
1022	PUNORTOR F.C. TAB 500MG	VAST RESOURCES PHARMACEUTICAL LTD	HK-58351	Unknown
1023	PUNORTOR TABS 500MG	VAST RESOURCES PHARMACEUTICAL LTD	HK-59022	Unknown
1024	PYMADON TABLETS 500MG	HITPHARM PHARMACEUTICAL CO LTD	HK-63795	Unknown
1025	PYROGESIC TAB 500MG	NATIONAL PHARMACEUTICAL CO LTD	HK-36093	No
1026	PYRUDON TAB 300MG	VAST RESOURCES PHARMACEUTICAL LTD	HK-60660	Unknown
1027	Q-COLD TAB	UNICORN LABORATORIES O/B AMERICAN UNICORN LABORATORIES LIMITED	HK-50181	Unknown
1028	QUALI-COLD JUNIOR TAB	QUALITY PHARM LAB LTD	HK-49468	Unknown
1029	QUALIMOL SYRUP 125MG/5ML	QUALITY PHARM LAB LTD	HK-55658	Unknown
1030	QUICK COLD CAP	QUALITY PHARM LAB LTD	HK-49620	Unknown
1031	QUICK-ACT COLD AND COUGH CAP	NEOCHEM PHARMACEUTICAL LABORATORIES LTD.	HK-54765	Unknown
1032	QUICKTON-FORTE CAP	VICKMANS LABORATORIES LTD	HK-54520	No
1033	RACEFINE TAB	UNICORN LABORATORIES O/B AMERICAN UNICORN LABORATORIES LIMITED	HK-46221	Unknown
1034	RALICOLD TABLET	EUROPHARM LAB CO LTD	HK-56407	No
1035	RAMANCOSIN CAP	SYNCO (H.K.) LIMITED	HK-56267	No
1036	RAPIZED CAP	EUROPHARM LAB CO LTD	HK-52211	No
1037	RESET PLUS TABLETS	NOBLE HEALTH CO., LTD	HK-63164	Unknown
1038	RESET TABLETS 500MG	NOBLE HEALTH CO., LTD	HK-63880	Unknown
1039	RHINAL CAP	NEOCHEM PHARMACEUTICAL LABORATORIES LTD.	HK-45616	Unknown
1040	RHINOTON CAP	VICKMANS LABORATORIES LTD	HK-55029	No
1041	ROCIOCOSIN CAP	SYNCO (H.K.) LIMITED	HK-56266	No
1042	ROSUX CAP	UNICORN LABORATORIES O/B AMERICAN UNICORN LABORATORIES LIMITED	HK-46479	Unknown

1043	ROTER DISSOLVABLE PARACETAMOL GRANULES 500MG	VEMEDIA SHINE LIMITED	HK-64150	Unknown
1044	ROYAL-COLD CAP	EUROPHARM LAB CO LTD	HK-52205	No
1045	S-PARAMOL (W) TAB 500MG	ADVANCE PHARMACEUTICAL COMPANY LIMITED	HK-45402	Unknown
1046	SADICON TAB	EUROPHARM LAB CO LTD	HK-56176	No
1047	SAMIK ACETAMINOPHEN TABLETS 500MG	SB PHARMA LIMITED	HK-67003	Unknown
1048	SANBRON CAP	APT PHARMA LIMITED	HK-44239	Unknown
1049	SANCMAN TABLETS	WAI LUN TRADING CO	HK-62131	Unknown
1050	SANIDEX CAP	EUROPHARM LAB CO LTD	HK-52256	No
1051	SANLADOL CAPLET	ADVANCE PHARMACEUTICAL COMPANY LIMITED	HK-52947	Unknown
1052	SANSOMIN TAB	MEYER PHARMACEUTICALS LTD	HK-41806	Unknown
1053	SAPLINGTAN POWDER	KAREN LABORATORIES O/B KAREN PHARMACEUTICAL CO LTD	HK-00286	Unknown
1054	SAPLINGTAN TAB	KAREN LABORATORIES O/B KAREN PHARMACEUTICAL CO LTD	HK-26279	Unknown
1055	SAPLINGTAN-S POWDER 500MG	KAREN LABORATORIES O/B KAREN PHARMACEUTICAL CO LTD	HK-35077	Unknown
1056	SARIDON TAB	BAYER HEALTHCARE LIMITED	HK-24091	Unknown
1057	SARISIL TABLETS	WELLDONE PHARMACEUTICALS LIMITED	HK-62543	Unknown
1058	SATO [COLD GO!] TAB	SATO PHARMACEUTICAL (HK) CO LTD	HK-52251	Unknown
1059	SELSA-COLD JUNIOR TAB	QUALITY PHARM LAB LTD	HK-49469	Unknown
1060	SERIMOL TAB 650MG	HEALTHCARE PHARMASCIENCE LIMITED	HK-48754	Unknown
1061	SERVASE CAP	EUROPHARM LAB CO LTD	HK-52203	No
1062	SETAMOL TAB 500MG	HOVID LIMITED	HK-35624	Unknown
1063	SHIELDOMIN CAP	VICKMANS LABORATORIES LTD	HK-54522	No
1064	SHILING TAB	KAREN LABORATORIES O/B KAREN PHARMACEUTICAL CO LTD	HK-00284	Unknown
1065	SHOTAMIN CAP	VICKMANS LABORATORIES LTD	HK-54449	No
1066	SINCOFUL EXTRA CAP	MEYER PHARMACEUTICALS LTD	HK-48611	Unknown
1067	SINOCON TABLET	WELL FAVOURED LTD	HK-63296	Unknown
1068	SINOF FLU & COUGH CAP	EUROPHARM LAB CO LTD	HK-55860	No
1069	SINSOLAX TAB	SYNMOSA BIOPHARMA (HONG KONG) COMPANY LIMITED	HK-59231	Unknown
1070	SINTALIN COLD CAP	QUALITY PHARM LAB LTD	HK-49471	Unknown
1071	SINUZEP TAB	MEYER PHARMACEUTICALS LTD	HK-48049	Unknown
1072	SIROP PARACETAMOL SYRUP 125MG/5ML	HON MAN MEDICINE (WING LEE) COMPANY	HK-45824	Unknown
1073	SMARIDA CAP	EUROPHARM LAB CO LTD	HK-52321	No
1074	SMARK COLD TABLETS	WELLDONE PHARMACEUTICALS LIMITED	HK-66005	Unknown
1075	SNIP TAB	STAR MEDICAL SUPPLIES LTD	HK-39103	No
1076	SOLARON TAB	EUROPHARM LAB CO LTD	HK-56175	No
1077	SONIREX TAB	EUROPHARM LAB CO LTD	HK-56263	No
1078	SPEDA-COLD CAP	EUROPHARM LAB CO LTD	HK-38434	No
1079	SPEED CAP	VICKMANS LABORATORIES LTD	HK-55380	No
1080	SPEED-ACT COLD AND COUGH CAP	NEOCHEM PHARMACEUTICAL LABORATORIES LTD.	HK-54763	Unknown
1081	SPEEDAT CAP	VICKMANS LABORATORIES LTD	HK-53569	No
1082	SPEEDY COLD AND COUGH CAP	NEOCHEM PHARMACEUTICAL LABORATORIES LTD.	HK-54767	Unknown
1083	STEPIN-COLD CAP	EUROPHARM LAB CO LTD	HK-52324	No
1084	STOP-COLD TAB	UNICORN LABORATORIES O/B AMERICAN UNICORN LABORATORIES LIMITED	HK-50180	Unknown
1085	SUDA-COLD JUNIOR TAB	QUALITY PHARM LAB LTD	HK-49619	Unknown
1086	SUPER SEA HORSE CAP	APT PHARMA LIMITED	HK-44255	Unknown
1087	SUPREME-A CAP	VICKMANS LABORATORIES LTD	HK-55373	No
1088	SURE-RELIEF COLD AND COUGH CAP	UNICORN LABORATORIES O/B AMERICAN UNICORN LABORATORIES LIMITED	HK-46478	Unknown
1089	SUTA EXTRA TAB	WELLDONE PHARMACEUTICALS LIMITED	HK-52487	Unknown
1090	SYN-PARCEMOL FORTE SUSPENSION 250MG/5ML	EUROPHARM LAB CO LTD	HK-64151	No
1091	TAKCOLIN CAP	VICKMANS LABORATORIES LTD	HK-53851	No
1092	TAMINOL CAPLETS 500MG	WELLDONE PHARMACEUTICALS LIMITED	HK-65169	Unknown
1093	TAPLEM CAP	APT PHARMA LIMITED	HK-44241	Unknown
1094	TARACET TABLETS	PRUDENTLINK LIMITED	HK-62152	Unknown
1095	TARGET 500 TAB 500MG	UNICORN LABORATORIES O/B AMERICAN UNICORN LABORATORIES LIMITED	HK-51873	Unknown
1096	TARPHEN TAB 500MG	NICE LABORATORIES LTD	HK-47562	Unknown
1097	TEFERSIL COLD CAP	QUALITY PHARM LAB LTD	HK-49472	Unknown
1098	TIFFY 125 SUPP 125MG	STAR MEDICAL SUPPLIES LTD	HK-33256	No
1099	TIFFY SUPP 250MG	STAR MEDICAL SUPPLIES LTD	HK-33040	No
1100	TINTEN EXTRA TAB	KAI YUEN PHARMACEUTICAL CO	HK-52854	Unknown
1101	TIPEX COLD RELIEF CAP	JEAN-MARIE PHARMACAL CO LTD	HK-28061	No
1102	TIPS CAP	KAREN LABORATORIES O/B KAREN PHARMACEUTICAL CO LTD	HK-59360	Unknown
1103	TOCAN TAB	EUROPHARM LAB CO LTD	HK-61213	No
1104	TOTAL COLD & FLU TAB	UNICORN LABORATORIES O/B AMERICAN UNICORN LABORATORIES LIMITED	HK-50286	Unknown
1105	TOTAL EXTRA CAPLET	ADVANCE PHARMACEUTICAL COMPANY LIMITED	HK-52950	Unknown
1106	TOZY EXTRA TAB (CHINTENG)	HITPHARM PHARMACEUTICAL CO LTD	HK-58604	Unknown
1107	TRAMACET TABLETS	VAST RESOURCES PHARMACEUTICAL LTD	HK-64581	Unknown
1108	TSOISPAN COLD TABLETS	WELLDONE PHARMACEUTICALS LIMITED	HK-67146	Unknown
1109	TSOITAII COLD TABLETS	WELLDONE PHARMACEUTICALS LIMITED	HK-66006	Unknown
1110	TYGENOL TAB 500MG	APT PHARMA LIMITED	HK-45064	Unknown
1111	U-C SYRUP 250MG/5ML	NEOCHEM PHARMACEUTICAL LABORATORIES LTD.	HK-33613	Unknown
1112	U-CETOL TAB 500MG	NEOCHEM PHARMACEUTICAL LABORATORIES LTD.	HK-27985	Unknown
1113	U-CHU PLUS ANALGESIC CAPLETS	VAST RESOURCES PHARMACEUTICAL LTD	HK-63643	Unknown
1114	U-COLCAP TAB	UNICORN LABORATORIES O/B AMERICAN UNICORN LABORATORIES LIMITED	HK-50269	Unknown
1115	U-J TAB 300MG	NEOCHEM PHARMACEUTICAL LABORATORIES LTD.	HK-33602	Unknown
1116	U-NORCETOL TAB	SYNCO (H.K.) LIMITED	HK-28632	No
1117	ULTRACET TABLETS	JOHNSON & JOHNSON (HONG KONG) LTD.	HK-66417	Unknown
1118	ULTRACOLD TAB	EUROPHARM LAB CO LTD	HK-56172	No
1119	ULTRAN TABLET	JULIUS CHEN & CO (HK) LTD	HK-60822	Unknown
1120	UNI-500 COLD TAB	UNICORN LABORATORIES O/B AMERICAN UNICORN LABORATORIES LIMITED	HK-50179	Unknown
1121	UNI-COLD AND COUGH CAP	NEOCHEM PHARMACEUTICAL LABORATORIES LTD.	HK-54766	Unknown
1122	UNI-COLD TAB	THE UNITED LABORATORIES LTD	HK-46809	Unknown
1123	UNI-FEBRIN SYRUP 120MG/5ML	JEAN-MARIE PHARMACAL CO LTD	HK-12677	Yes
1124	UNI-FEBRIN TAB 500MG	NEOCHEM PHARMACEUTICAL LABORATORIES LTD.	HK-38571	Unknown
1125	UNI-PAINGETEX TABLET	SYNCO (H.K.) LIMITED	HK-62530	No
1126	UNI-PAMOL (ORANGE) TAB 500MG	JEAN-MARIE PHARMACAL CO LTD	HK-38318	No
1127	UNIGAN TAB	HEALTH ALLIANCE INTERNATIONAL CO LTD	HK-43954	Yes
1128	UPHAMOL SUSPENSION 250 (ORANGE) 250MG/5ML	VAST RESOURCES PHARMACEUTICAL LTD	HK-54440	Unknown
1129	VANECOLD TAB	EUROPHARM LAB CO LTD	HK-56171	No
1130	VICKCOLIN CAP	VICKMANS LABORATORIES LTD	HK-53612	No
1131	VICKMANS ACETAMOL (BLUE) TAB 500MG	NEOCHEM PHARMACEUTICAL LABORATORIES LTD.	HK-37112	Unknown
1132	VICKMANS ACETAMOL (YELLOW) TAB 500MG	VICKMANS LABORATORIES LTD	HK-37111	No
1133	VICKOFLU TABLETS	VICKMANS LABORATORIES LTD	HK-65201	No
1134	VIDA COLD TAB FOR CHILDREN	VICKMANS LABORATORIES LTD	HK-19392	No
1135	VIDA-COLD-PLUS CAP	VICKMANS LABORATORIES LTD	HK-19928	No
1136	VIDATAPP FORTE IMPROVED CAP	VICKMANS LABORATORIES LTD	HK-23966	No
1137	VIMIX-COLD CAP	EUROPHARM LAB CO LTD	HK-52200	No
1138	VISKZEP-CHILD COLD TAB	MEYER PHARMACEUTICALS LTD	HK-58776	Unknown
1139	VISONEX TAB	EUROPHARM LAB CO LTD	HK-56174	No
1140	WAIMIN FORTE CAP	KAREN LABORATORIES O/B KAREN PHARMACEUTICAL CO LTD	HK-46120	Unknown
1141	WEELGAC-S CAP	NATIONAL PHARMACEUTICAL CO LTD	HK-49583	No
1142	WELLCOMIN CAP	VICKMANS LABORATORIES LTD	HK-54451	No
1143	WENNEX CAP	EUROPHARM LAB CO LTD	HK-49247	No
1144	WENTICOLD TAB	EUROPHARM LAB CO LTD	HK-56177	No
1145	WINFLU CAP	NATIONAL PHARMACEUTICAL CO LTD	HK-49174	No
1146	WONTRAN TABLETS	HEALTHCARE PHARMASCIENCE LIMITED	HK-62389	Unknown
1147	X-COLD CAP	UNICORN LABORATORIES O/B AMERICAN UNICORN LABORATORIES LIMITED	HK-46476	Unknown
1148	XENIDEL TABLETS	WELLDONE PHARMACEUTICALS LIMITED	HK-63020	Unknown
1149	XENZEP CAP	KAREN LABORATORIES O/B KAREN PHARMACEUTICAL CO LTD	HK-59273	Unknown
1150	XICODINE EXTRA CAP	MEYER PHARMACEUTICALS LTD	HK-48911	Unknown
1151	XYKAA EXTEND PROLONGED RELEASE TABLET 650MG	EVERCARE PHARMACEUTICAL CO. LTD.	HK-61400	Unknown
1152	XYKAA RAPID TABLETS 500MG	EVERCARE PHARMACEUTICAL CO. LTD.	HK-64868	Unknown

1153		YES COLD & FLU TAB	UNICORN LABORATORIES O/B AMERICAN UNICORN LABORATORIES LIMITED	HK-50177	Unknown
1154		YUDRONG COLD TABLETS	WELLDONE PHARMACEUTICALS LIMITED	HK-66010	Unknown
1155		ZEN COLD FLU & COUGH CAP	EUROPHARM LAB CO LTD	HK-55809	No
1156		ZENECO CAP	MEYER PHARMACEUTICALS LTD	HK-48828	Unknown
1157		ZENIDONE CAP	KAREN LABORATORIES O/B KAREN PHARMACEUTICAL CO LTD	HK-58799	Unknown
1158		ZENNACO CAP	MEYER PHARMACEUTICALS LTD	HK-37210	Unknown
1159		ZERTICOLD CAP	EUROPHARM LAB CO LTD	HK-52207	No
1160		ZIMACO TABLETS	LAFARGE CO., LIMITED	HK-65425	Unknown
1161		ZITAR TABLETS	WELLDONE PHARMACEUTICALS LIMITED	HK-62544	Unknown
1162		ZUTAN TABLETS	EUROPHARM LAB CO LTD	HK-61488	No
1163	**CLOXACILLIN**	AMPICLOXA-500 CAP	NIDOWAY INVESTMENT LTD	HK-43969	Unknown
1164		ANSINI CAP	VICKMANS LABORATORIES LTD	HK-43607	No
1165		APT-AMPICLOXA CAP	VICKMANS LABORATORIES LTD	HK-41426	No
1166		CLOXACAP CAP 500MG	HOVID LIMITED	HK-41332	Unknown
1167		CLOXACILLIN CAP 250MG	VICKMANS LABORATORIES LTD	HK-20056	No
1168		CLOXACILLIN POWDER FOR SOLUTION FOR IM/IV INJECTION 500MG (NORTH CHINA PHARMACEUTICAL)	JINDUN PHARMA (H.K.) LIMITED	HK-61300	Unknown
1169		CLOXAMOX CAP	VICKMANS LABORATORIES LTD	HK-50448	No
1170		CLOXAMPI CAP	VICKMANS LABORATORIES LTD	HK-21260	No
1171		CLOXAMPICIN CAP	YAT SENG TRADING CO	HK-45681	Unknown
1172		COMPLICILLIN CAP	HANG LUNG TRADING (H.K.) CO	HK-55107	Unknown
1173		COMPLIPEN CAP	HANG LUNG TRADING (H.K.) CO	HK-35276	Unknown
1174		DICLOMOX CAP	BRIGHT FUTURE PHARMACEUTICALS FACTORY O/B BRIGHT FUTURE PHARMACEUTICAL LABORATORIES LIMITED	HK-53646	Unknown
1175		DICLONOX CAP 250MG	DELTAPHARM LIMITED	HK-45100	Unknown
1176		FLUCLOXACILLIN CAP 250MG (ATHLONE)	THE INTERNATIONAL MEDICAL COMPANY LIMITED	HK-51790	Unknown
1177		FLUCLOXACILLIN CAP 250MG (BRIGHT FUTURE)	BRIGHT FUTURE PHARMACEUTICALS FACTORY O/B BRIGHT FUTURE PHARMACEUTICAL LABORATORIES LIMITED	HK-42222	Unknown
1178		FLUCLOXACILLIN ORAL SOLUTION 125MG/5ML	THE INTERNATIONAL MEDICAL COMPANY LIMITED	HK-50542	Unknown
1179		LAMPICIN FORT CAP	BRIGHT FUTURE PHARMACEUTICALS FACTORY O/B BRIGHT FUTURE PHARMACEUTICAL LABORATORIES LIMITED	HK-37322	Unknown
1180		LOXAMP CAPSULES	SWEDISH TRADING COMPANY LIMITED	HK-63700	Unknown
1181		MONOCLOX CAP 250MG	STAR MEDICAL SUPPLIES LTD	HK-19365	No
1182		NEO A C CAP	VICKMANS LABORATORIES LTD	HK-17913	No
1183		NICE AMCLOX CAP	VICKMANS LABORATORIES LTD	HK-17895	No
1184		ODICOZA CAP 250MG	BRIGHT FUTURE PHARMACEUTICALS FACTORY O/B BRIGHT FUTURE PHARMACEUTICAL LABORATORIES LIMITED	HK-55749	Unknown
1185		PAN-CLOXACILLIN FOR INJ 500MG	DCH AURIGA (HONG KONG) LIMITED - UNIVERSAL DIVISION	HK-51842	Unknown
1186		REICHLOX CAP 500MG	APT PHARMA LIMITED	HK-53085	Unknown
1187		SYNPICLOX CAP	VICKMANS LABORATORIES LTD	HK-55753	No
1188		ZIEFMYCIN CAP 250MG	YUNG SHIN CO LTD	HK-34703	Unknown
1189	**ERYTHROMYCIN**	APO-ERYTHRO-BASE TAB 250MG	HIND WING CO LTD	HK-36578	Unknown
1190		E-MYCIN ERYTHROMYCIN TAB 400MG	LUEN CHEONG HONG LTD	HK-38817	Unknown
1191		ELOSONE SUSPENSION 125MG/5ML	QUALITY PHARM LAB LTD	HK-09258	Unknown
1192		ERISMYCINE CAP 250MG	CHRISTO PHARM LTD	HK-21108	No
1193		ERISMYCINE SUSP 125MG BASE/5ML	CHRISTO PHARM LTD	HK-20451	No
1194		ERISMYCINE TAB 250MG	CHRISTO PHARM LTD	HK-24156	Yes
1195		EROGRAN GRANULES FOR SYRUP 200MG/5ML	HOVID LIMITED	HK-38123	Unknown
1196		EROTAB 250 TAB 250MG	HOVID LIMITED	HK-35784	Unknown
1197		ERYSOL POWDER FOR ORAL SUSP 200MG/5ML	FP HEALTHCARE LIMITED	HK-30882	Unknown
1198		ERYTHROBASE TAB 250MG ENTERIC-COATED	EUROPHARM LAB CO LTD	HK-54317	Yes
1199		ERYTHROMYCIN	SUNRISE TRADING CO	HK-56042	Unknown
1200		ERYTHROMYCIN CAP 250MG	VAST RESOURCES PHARMACEUTICAL LTD	HK-52674	Unknown
1201		ERYTHROMYCIN CAP 250MG	VICKMANS LABORATORIES LTD	HK-35070	No
1202		ERYTHROMYCIN ESTOLATE	SUNRISE TRADING CO	HK-47497	Unknown
1203		ERYTHROMYCIN FOR SUSP 125MG/5ML	HON MAN MEDICINE (WING LEE) COMPANY	HK-46050	Unknown
1204		IROSIN CAP 250MG	VAST RESOURCES PHARMACEUTICAL LTD	HK-61467	Unknown
1205		NEO-THROCIN CAP 250MG	NEOCHEM PHARMACEUTICAL LABORATORIES LTD.	HK-06405	Unknown
1206		NICE-ERYCIN CAP 250MG	VICKMANS LABORATORIES LTD	HK-07399	No
1207		PORPHYROCIN TAB 250MG	STAR MEDICAL SUPPLIES LTD	HK-40181	No
1208		RYPED ORAL SUSP 125MG 5ML	MEDREICH FAR EAST LIMITED	HK-59507	Unknown
1209	**ALCOHOL**	ALCOHOL DE MENTHE DE RICQLES	THE INTERNATIONAL MEDICAL COMPANY LIMITED	HK-03206	Unknown
1210		BETACARE SORE THROAT HONEY & LEMON LOZENGES 0.6MG/1.2MG	MUNDIPHARMA (HONG KONG) LIMITED	HK-67390	Unknown
1211		COLDANGIN LOZENGES	FORTUNE NATIONAL (HONG KONG) LIMITED	HK-46071	Unknown
1212		LOGICIN RAPID RELIEF LOZ MENTHOL & EUCALYPTUS	ASPEN ASIA COMPANY LIMITED	HK-45249	Unknown
1213		MADAME PEARL'S ANTISEPTIC LOZENGES (HONEY LEMON FLAVOUR)	LUXEMBOURG MEDICINE CO LTD	HK-57961	No
1214		MILLAKOL LOZENGE	MEDILINE (HONG KONG) COMPANY LIMITED	HK-62426	Unknown
1215		STREPSILS + HONEY & LEMON ANTISEPTIC LOZ	RECKITT BENCKISER HONG KONG LTD	HK-19288	No
1216		THROATSIL LOZENGE - HONEY & LEMON	DELTAPHARM LIMITED	HK-54273	Unknown
1217	**ALLOPURINOL**	ADENO TABLETS 100MG	WELLDONE PHARMACEUTICALS LIMITED	HK-66894	Unknown
1218		ALLNOL PLUS TAB 300MG	SYNCO (H.K.) LIMITED	HK-44715	No
1219		ALLNOL TAB 100MG	JEAN-MARIE PHARMACAL CO LTD	HK-44702	No
1220		ALLO 100 TAB 100MG	WAI LUN TRADING CO	HK-58279	Unknown
1221		ALLOPIN TAB 100MG	TRENTON-BOMA LTD	HK-49693	No
1222		ALLOPRANOL TAB 100MG	JEAN-MARIE PHARMACAL CO LTD	HK-38604	No
1223		ALLOPURINOL TAB 100MG	CHRISTO PHARM LTD	HK-30121	No
1224		ALLOPURINOL TAB 100MG	YAT SENG TRADING CO	HK-61016	Unknown
1225		ALLOPURINOL TAB 100MG	HOVID LIMITED	HK-35846	Unknown
1226		ALLOPURINOL TAB 100MG	JEAN-MARIE PHARMACAL CO LTD	HK-08608	No
1227		ALLOPURINOL TAB 100MG	TEVA PHARMACEUTICAL HONG KONG O/B TEVA PHARMACEUTICAL HONG KONG LIMITED	HK-23004	Unknown
1228		ALLOPURINOL TAB 100MG (QUALITY)	QUALITY PHARM LAB LTD	HK-36267	Unknown
1229		ALLOPURINOL TAB 300MG	SYNCO (H.K.) LIMITED	HK-21789	No
1230		ALLOPURINOL TABLETS 100MG	KAI YUEN PHARMACEUTICAL CO	HK-62651	Unknown
1231		ALLOPURINOL TABLETS 100MG	PRUDENTLINK LIMITED	HK-65526	Unknown
1232		ALLOPURINOL TCK TABLETS 100MG	KAERU PHARMACEUTICAL (HK) LIMITED	HK-66571	Unknown
1233		APO-ALLOPURINOL TAB 100MG	HIND WING CO LTD	HK-02655	Unknown
1234		EFORMAT ALLOPURINOL TABLETS 300MG	WELLDONE PHARMACEUTICALS LIMITED	HK-66534	Unknown
1235		ENPUROL TABLETS 300MG	WELLDONE PHARMACEUTICALS LIMITED	HK-66630	Unknown
1236		ESLORIC 100 TAB 100MG	UNIPHARM (HONG KONG) LIMITED	HK-59653	Unknown
1237		MARINOL TABLET 300MG	SYNCO (H.K.) LIMITED	HK-51361	No
1238		PURIDE 300 TAB 300MG	NATURAL HEALTH RESOURCES COMPANY LIMITED	HK-50451	No
1239		SYNORID TAB 100MG	SYNMOSA BIOPHARMA (HONG KONG) COMPANY LIMITED	HK-52620	Unknown
1240		SYNPURINOL 300MG TAB	SYNCO (H.K.) LIMITED	HK-37157	No
1241		TOSPAN ALLOPURINOL TABLETS 100MG	WELLDONE PHARMACEUTICALS LIMITED	HK-66895	Unknown
1242		WILLIPO ALLOPURINOL TABLETS 300MG	WELLDONE PHARMACEUTICALS LIMITED	HK-66538	Unknown
1243		ZYLORIC 300 TAB 300MG	LUEN CHEONG HONG LTD	HK-10041	Unknown
1244	**CEFUROXIME**	ALKOXIME 250 TABLETS 250MG	NIDOWAY INVESTMENT LTD	HK-65350	Unknown
1245		APROKAM POWDER FOR SOLUTION FOR INJECTION 50MG	HONG KONG MEDICAL SUPPLIES LTD	HK-66973	No
1246		APT-CEFUROXIME TAB 250MG	VICKMANS LABORATORIES LTD	HK-57111	No
1247		AXACEF TAB 250MG	MEDREICH FAR EAST LIMITED	HK-57855	Unknown
1248		AXCEF TABLETS 250MG	LSB (HK) LIMITED	HK-61702	Unknown
1249		AXCEL CEFUROXIME-250 CAPSULES 250MG	KOTRA PHARMA (HONG KONG) COMPANY	HK-63784	Unknown
1250		AXETINE FOR INJ 750MG	STAR MEDICAL SUPPLIES LTD	HK-42357	No
1251		CEFUROXIM STADA 250 TAB 250MG	HONG KONG MEDICAL SUPPLIES LTD	HK-56122	Yes
1252		CEFUROXIME 750MG FOR INJ AND DEXTROSE INJ	B. BRAUN MEDICAL (HK) LTD	HK-59240	No
1253		CEFUROXIME AXETIL TAB 250MG	CEUTICAL TRADING COMPANY LIMITED	HK-53691	Unknown
1254		CEFUROXIME FOR INJECTION 0.75G (SHENZHEN LIJIAN)	CEUTICAL TRADING COMPANY LIMITED	HK-61701	Unknown
1255		CEFUROXIME SODIUM FOR INJ 750MG	JINDUN PHARMA (H.K.) LIMITED	HK-52841	Unknown
1256		CEFUROXIME-SALUBRIS POWDER FOR SOLUTION FOR INJECTION 750MG	I & C (HONG KONG) LIMITED	HK-63423	Unknown
1257		CEFUSAN TAB 250MG	EUGENPHARM INTERNATIONAL LIMITED	HK-54772	Unknown
1258		CEFUXIME 250 TAB 250MG	AUROBINDO PHARMA LIMITED	HK-59694	Unknown
1259		CMAXID-250 TABLET 250MG	HOVID LIMITED	HK-62283	Unknown

1260		FARMACEF TAB 250MG	TRENTON-BOMA LTD	HK-54616	Yes
1261		FUROCEF TABLETS 250MG	HEALTHCARE PHARMASCIENCE LIMITED	HK-61759	Unknown
1262		SEFUXIM TAB 250MG	CEUTICAL TRADING COMPANY LIMITED	HK-55257	Unknown
1263		VICK-CEFUROXIME TAB 250MG	VICKMANS LABORATORIES LTD	HK-57350	No
1264		XORIMAX POWDER FOR ORAL SUSP 125MG/5ML	NOVARTIS PHARMACEUTICALS (HK) LIMITED	HK-58622	No
1265		XORIMAX TAB 250MG	NOVARTIS PHARMACEUTICALS (HK) LIMITED	HK-58709	No
1266		XYLID TABLET 250MG	JACOBSON MARKETING LIMITED	HK-61818	No
1267		ZENATOP 250 TAB 250MG	JULIUS CHEN & CO (HK) LTD	HK-59549	Unknown
1268		ZINACEF FOR INJ 1.5G	GLAXOSMITHKLINE LIMITED	HK-07015	Unknown
1269		ZINNAT FOR SUSPENSION 125MG/5ML	GLAXOSMITHKLINE LIMITED	HK-48869	Unknown
1270		ZINNAT TAB 125MG	GLAXOSMITHKLINE LIMITED	HK-29695	Unknown
1271	**BENZYLPENICILLIN**	NORABEN FOR IM INJ 1200000IU	JINDUN PHARMA (H.K.) LIMITED	HK-60852	Unknown
1272		PAN-PENICILLIN G SOD FOR INJ 1M IU	DCH AURIGA (HONG KONG) LIMITED - UNIVERSAL DIVISION	HK-43500	Unknown
1273	**CIPROFLOXACIN**	ARISTIN-C SOLUTION FOR INFUSION 200MG/100ML	HONG KONG MEDICAL SUPPLIES LTD	HK-66096	No
1274		CIFLOX TABLETS 500MG	YUNG SHIN CO LTD	HK-67051	Unknown
1275		CIPIDE TAB 250MG	APT PHARMA LIMITED	HK-45024	Unknown
1276		CIPQUIN 500 TAB 500MG	KAI YUEN PHARMACEUTICAL CO	HK-53227	Unknown
1277		CIPROCINA INJ 0.2%W/V	BAXTER HEALTHCARE LTD	HK-55724	Unknown
1278		CIPROFLOXACIN CAPSULES 0.25G	HIND WING CO LTD	HK-61562	Unknown
1279		CIPROFLOXACIN HEC PHARM TABLETS 250 MG	CEUTICAL TRADING COMPANY LIMITED	HK-65566	Unknown
1280		CIPROFLOXACIN I&C SOLUTION FOR INFUSION 200MG/100ML	I & C (HONG KONG) LIMITED	HK-66956	Unknown
1281		CIPROFLOXACIN NORMON SOLUTION FOR INFUSION 400MG/200ML	JACOBSON MARKETING LIMITED	HK-66656	No
1282		CIPROFLOXACIN TAB 250MG	JEAN-MARIE PHARMACAL CO LTD	HK-50830	Yes
1283		CIPROFLOXACIN TAB 500MG	YAT SENG TRADING CO	HK-57939	Unknown
1284		CIPROFLOXACIN TABLETS USP 250MG	SWEDISH TRADING COMPANY LIMITED	HK-66887	Unknown
1285		CIPROFLOXACIN-HAMELN 2MG/ML SOLUTION FOR INFUSION 100ML	MEKIM LTD	HK-62345	Unknown
1286		CIPROFLOXACIN-TEVA TAB 250MG	TEVA PHARMACEUTICAL HONG KONG O/B TEVA PHARMACEUTICAL HONG KONG LIMITED	HK-55105	Unknown
1287		CIPROFLOXACINA FARMOZ TAB 500MG	TRENTON-BOMA LTD	HK-60494	Yes
1288		CIPROFLOXACINO CINFA TAB 250MG	REICH PHARM LIMITED	HK-51580	Unknown
1289		CIPROVICK CAP 250MG	VICKMANS LABORATORIES LTD	HK-53590	No
1290		CIPROXIN INFUSION IV 0.2%	BAYER HEALTHCARE LIMITED	HK-29934	Unknown
1291		CIPROXIN TAB 250MG	BAYER HEALTHCARE LIMITED	HK-29936	Unknown
1292		CIPROXYL 500 TAB 500MG	TRENTON-BOMA LTD	HK-50797	No
1293		CIROK TAB 250MG	YIK KWAN PHARMACEUTICALS CO LTD	HK-49883	Unknown
1294		CIVELL TABLETS 500MG	HEALTHCARE PHARMASCIENCE LIMITED	HK-61911	Unknown
1295		COBAY TABLET 250MG	MEDILINE (HONG KONG) COMPANY LIMITED	HK-62335	Unknown
1296		CYFLOXIN TAB 250MG	JEAN-MARIE PHARMACAL CO LTD	HK-50522	Yes
1297		CYPLOX TAB 500MG	MEDREICH FAR EAST LIMITED	HK-55718	Unknown
1298		FLONTIN TABLETS 250MG	HEALTHCARE PHARMASCIENCE LIMITED	HK-61761	Unknown
1299		GIROFLOX SOLUTION FOR INFUSION 200MG/100ML	TRENTON-BOMA LTD	HK-59033	No
1300		GONNING TAB 250MG	US NANO FOOD AND DRUG (HK) LIMITED	HK-47763	Unknown
1301		HIPPRO FORTE TAB 500MG	DELTAPHARM LIMITED	HK-45556	Unknown
1302		HIPPRO TAB 250MG	DELTAPHARM LIMITED	HK-41151	Unknown
1303		INTERFLOX 500 TAB 500MG	NATURAL HEALTH RESOURCES COMPANY LIMITED	HK-50313	Yes
1304		JMP CIPROFLOXACIN TAB 250MG	JEAN-MARIE PHARMACAL CO LTD	HK-53328	Yes
1305		KORUS-CIPRO TAB 500MG	LSB (HK) LIMITED	HK-61018	Unknown
1306		LOXIN TAB 250MG	JEAN-MARIE PHARMACAL CO LTD	HK-53688	Yes
1307		MEDOCIPRIN 250 TAB 250MG	MEDOCHEMIE (HONG KONG) LIMITED	HK-34680	Unknown
1308		MEDOCIPRIN 500 TAB 500MG	MEDOCHEMIE (HONG KONG) LIMITED	HK-34679	Unknown
1309		POLI-CIFLOXIN 250 TAB 250MG	NATURAL HEALTH RESOURCES COMPANY LIMITED	HK-47031	Yes
1310		PROXACIN TAB 250MG	MEDREICH FAR EAST LIMITED	HK-52875	Unknown
1311		QUINTOR-250 TAB 250MG	TRENTON-BOMA LTD	HK-60960	No
1312		TROXECIN TAB 250MG	LSB (HK) LIMITED	HK-61123	Unknown
1313		UFEXIL SOLUTION FOR IV INFUSION 200MG/100ML	HIND WING CO LTD	HK-61723	Unknown
1314		UROXIN TAB 250MG	HEALTH ALLIANCE INTERNATIONAL CO LTD	HK-44530	Yes
1315		UTAHZONE TAB 250MG	HEALTHCARE PHARMASCIENCE LIMITED	HK-42779	Unknown
1316		VESPROCIN-250 TABLETS 250MG	VAST RESOURCES PHARMACEUTICAL LTD	HK-63391	Unknown
1317		VIPROLOX 250 TAB 250MG	DCH AURIGA (HONG KONG) LIMITED - UNIVERSAL DIVISION	HK-52577	Unknown
1318		VOLINOL TAB 500MG	PERFECT GROUPS LTD	HK-42406	Unknown
1319		ZOXAN-250 TAB 250MG	STAR MEDICAL SUPPLIES LTD	HK-51940	Yes
1320	**CARBIMAZOLE**	CAMAZOL TABLETS 5MG	FP HEALTHCARE LIMITED	HK-65516	Unknown
1321		CARBIMAZOLE 5 TAB 5MG (REMEDICA)	HEALTHCARE PHARMASCIENCE LIMITED	HK-52760	Unknown
1322		CARBIMAZOLE TAB 5MG	CHRISTO PHARM LTD	HK-26101	No
1323		CARBIROID TAB 5MG	HOVID LIMITED	HK-35972	Unknown
1324	**CEPHALEXIN**	AMYSPORIN CAP 250MG	VICKMANS LABORATORIES LTD	HK-28762	No
1325		APO-CEPHALEX TAB 250MG	HIND WING CO LTD	HK-35607	Unknown
1326		APT-CEPHALEX CAPSULES 250MG	VICKMANS LABORATORIES LTD	HK-65322	No
1327		AXCEL CEPHALEXIN-125 POWDER FOR ORAL SUSPENSION 125MG/5ML	KOTRA PHARMA (HONG KONG) COMPANY	HK-64466	Unknown
1328		CEFACIN CAP 500MG	BRIGHT FUTURE PHARMACEUTICALS FACTORY O/B BRIGHT FUTURE PHARMACEUTICAL LABORATORIES LIMITED	HK-38178	Unknown
1329		CEFALEXIN 500 CAP 500MG	UNICORN LABORATORIES O/B AMERICAN UNICORN LABORATORIES LIMITED	HK-48904	Unknown
1330		CEFASTAD 500 CAP 500MG	STADA PHARMACEUTICALS (ASIA) LTD	HK-60641	Unknown
1331		CELEXIN GRANULES 125MG/5ML	HOVID LIMITED	HK-38728	Unknown
1332		CEPHALEXIN CAP 250MG	HEALTHCARE PHARMASCIENCE LIMITED	HK-44819	Unknown
1333		CEPHALEXIN CAP 250MG	VICKMANS LABORATORIES LTD	HK-26207	No
1334		CEPHALEXIN CAPSULES 500MG	WINGS PHARMACEUTICAL LTD	HK-65719	No
1335		CEPHALEXIN STADA CAP 250MG	STADA PHARMACEUTICALS (ASIA) LTD	HK-57266	Unknown
1336		CEPHALEXYL-250 CAP 250MG	TRENTON-BOMA LTD	HK-49783	No
1337		CEPHANMYCIN CAP 250MG (YUNG SHIN)	YUNG SHIN CO LTD	HK-34698	Unknown
1338		CEPHIN CAP 250MG	TRENTON-BOMA LTD	HK-49695	No
1339		CEPHIN DRY SYRUP 125MG/5ML	TRENTON-BOMA LTD	HK-49632	No
1340		IKODIN CAP 250MG	WILCOME PHARMACEUTICAL CO LTD	HK-17392	Unknown
1341		KOFLEX-250 CAP 250MG	NIDOWAY INVESTMENT LTD	HK-44815	Unknown
1342		MA-CEPHALEXIN CAP 250MG	APT PHARMA LIMITED	HK-47444	Unknown
1343		MA-CEPHALEXIN CAP 500MG	VICKMANS LABORATORIES LTD	HK-47447	No
1344		MEDICEPHAL CAP 500MG	LSB (HK) LIMITED	HK-60537	Unknown
1345		MEDOLEXIN 500 CAP 500MG	MEDOCHEMIE (HONG KONG) LIMITED	HK-22591	Unknown
1346		NEO-CEPHA CAP 250MG	VICKMANS LABORATORIES LTD	HK-25625	No
1347		NEO-CEPHA DRY SYRUP 250MG/5ML	VICKMANS LABORATORIES LTD	HK-43671	No
1348		NEOFLEXIN CAPSULES 250MG	VICKMANS LABORATORIES LTD	HK-65324	No
1349		NICE-FLEXIN CAP 500MG	VICKMANS LABORATORIES LTD	HK-26070	No
1350		ORA-C CAP 250MG	VICKMANS LABORATORIES LTD	HK-50831	No
1351		ORA-C GRANULES 250MG/5ML	VICKMANS LABORATORIES LTD	HK-40842	No
1352		PARAFLEX CAP 250MG	YAT SENG TRADING CO	HK-45680	Unknown
1353		SOFILEX CAP 250MG	FP HEALTHCARE LIMITED	HK-43916	Unknown
1354		SOFILEX FOR ORAL SUSPENSION 125MG/5ML	FP HEALTHCARE LIMITED	HK-44546	Unknown
1355		SOLULEXIN CAP 250MG	WINGS PHARMACEUTICAL LTD	HK-43400	No
1356		SOLULEXIN GRANULES FOR SYRUP 125MG/5ML	WINGS PHARMACEUTICAL LTD	HK-43399	No
1357		SYNLEXIN CAP 500MG	VICKMANS LABORATORIES LTD	HK-38657	No
1358		SYNTOLEXIN 250 CAP 250MG	CEUTICAL TRADING COMPANY LIMITED	HK-24629	Unknown
1359		UPHALEXIN CAP 250MG	VAST RESOURCES PHARMACEUTICAL LTD	HK-59184	Unknown
1360		VICKCEPHA CAPSULES 250MG	VICKMANS LABORATORIES LTD	HK-65999	No
1361	**LEVOFLOXACIN**	AMESOL TABLETS 250MG	MEDOCHEMIE (HONG KONG) LIMITED	HK-66425	Unknown
1362		APO-LEVOFLOXACIN TAB 250MG	HIND WING CO LTD	HK-61140	Unknown
1363		CRALEVO TAB 250MG	APT PHARMA LIMITED	HK-56929	Unknown
1364		CRAVIT IV SOLUTION FOR INFUSION 5MG/ML	DAIICHI SANKYO HONG KONG LIMITED	HK-45236	Unknown
1365		CRAVIT TAB 250MG	DAIICHI SANKYO HONG KONG LIMITED	HK-51495	Unknown
1366		JECEFARMA LEVOFLOXACIN TABLETS 250MG	JULIUS CHEN & CO (HK) LTD	HK-62957	Unknown
1367		L-STAFLOXIN 250 TABLETS 250MG	HONG KONG MEDICAL SUPPLIES LTD	HK-66419	Yes
1368		LEFELO TAB 250MG	EUGENPHARM INTERNATIONAL LIMITED	HK-58151	Unknown
1369		LEFLOX TAB 250MG	CHARIOT PHARMA LIMITED	HK-58105	Unknown

1370		LEFOVID-500 TABLETS 500MG	HOVID LIMITED	HK-65501	Unknown
1371		LEO 250 TABLET 250MG	DELTAPHARM LIMITED	HK-58705	Unknown
1372		LEVAXCIN 250 TABLETS 250MG	SWEDISH TRADING COMPANY LIMITED	HK-61474	Unknown
1373		LEVOCIN 250 TAB 250MG	LAFARGE CO., LIMITED	HK-59370	Unknown
1374		LEVOFLOXACIN HEC PHARM TABLETS 250MG	CEUTICAL TRADING COMPANY LIMITED	HK-65254	Unknown
1375		LEVOFLOXACIN KABI SOLUTION FOR INFUSION 250MG/50ML	FRESENIUS KABI HONG KONG LIMITED	HK-65006	Unknown
1376		LEVOFLOXACIN NORMON SOLUTION FOR INFUSION 500MG/100ML	JACOBSON MARKETING LIMITED	HK-66445	No
1377		LEVOFLOXACIN SANDOZ TAB 250MG	NOVARTIS PHARMACEUTICALS (HK) LIMITED	HK-60409	Yes
1378		LEVOFLOXACIN STADA TABLETS 250MG	HONG KONG MEDICAL SUPPLIES LTD	HK-63952	Yes
1379		LEVOFLOXACIN TABLETS 250MG	I & C (HONG KONG) LIMITED	HK-63089	Unknown
1380		LEVOFLOXACIN-HAMELN SOLUTION FOR INFUSION 5MG/ML	MEKIM LTD	HK-62897	Unknown
1381		LEVOFLOXACIN-PHARMATHEN SOLUTION FOR INFUSION 500MG/100ML	I & C (HONG KONG) LIMITED	HK-63088	Unknown
1382		LEVOFLOXACINA FARMOZ SOLUTION FOR INF 5MG/ML	TRENTON-BOMA LTD	HK-60112	No
1383		LEVOKILZ 250 TABLETS 250MG	HANG LUNG TRADING (H.K.) CO	HK-67268	Unknown
1384		LEVORAN 250 TABLETS 250MG	R. MANSTIEN (AUSTRALIA) LIMITED	HK-66438	Unknown
1385		LEVOSTA TAB 100MG	JULIUS CHEN & CO (HK) LTD	HK-51269	Unknown
1386		LEVOXA TAB 250MG	TEVA PHARMACEUTICAL HONG KONG O/B TEVA PHARMACEUTICAL HONG KONG LIMITED	HK-60187	Unknown
1387		LEVROXIN TABLETS 100MG	HEALTHCARE PHARMASCIENCE LIMITED	HK-62227	Unknown
1388		LEXA CAPLET 500MG	HANG LUNG TRADING (H.K.) CO	HK-57050	Unknown
1389		LEXACIN TAB 100MG	LSB (HK) LIMITED	HK-60928	Unknown
1390		VICK-LEVOXA TAB 100MG	VICKMANS LABORATORIES LTD	HK-56369	No
1391		VOCIN 500 TABLETS 500MG	HEALTH ALLIANCE INTERNATIONAL CO LTD	HK-65662	Yes
1392		ZILEE 250 TAB 250MG	STAR MEDICAL SUPPLIES LTD	HK-56960	Yes
1393	**METRONIDAZOLE**	APO-METRONIDAZOLE TAB 250MG	HIND WING CO LTD	HK-40682	Unknown
1394		AXCEL METRONIDAZOLE-200 TABLETS 200MG	KOTRA PHARMA (HONG KONG) COMPANY	HK-63015	Unknown
1395		DYNIN TAB 250MG	SWISS PHARMACEUTICAL CO. LIMITED	HK-17390	Unknown
1396		FILMET 200 TABLETS 200MG	EUGENPHARM INTERNATIONAL LIMITED	HK-66235	Unknown
1397		FITGYL TAB 200MG	CHRISTO PHARM LTD	HK-37012	No
1398		FLAGYL TAB 200MG	SANOFI HONG KONG LIMITED	HK-31150	Unknown
1399		FLAGYL-S SUSP 200MG/5ML	SANOFI HONG KONG LIMITED	HK-31148	Unknown
1400		FROTIN E.S.C. TAB 250MG	YUNG SHIN CO LTD	HK-34638	Unknown
1401		FUZUIN S.C. TAB 250MG	HITPHARM PHARMACEUTICAL CO LTD	HK-49226	Unknown
1402		JECEFARMA METRONIDAZOLE TABLETS 200MG	JULIUS CHEN & CO (HK) LTD	HK-63914	Unknown
1403		KLONT TAB 200MG	STAR MEDICAL SUPPLIES LTD	HK-30198	No
1404		MEGYL TAB 200MG	MEYER PHARMACEUTICALS LTD	HK-38030	Unknown
1405		METOLE TAB 200MG	NEOCHEM PHARMACEUTICAL LABORATORIES LTD.	HK-44563	Unknown
1406		METROGYL 200 TAB 200MG	LUEN CHEONG HONG LTD	HK-33005	Unknown
1407		METROMILL SOLUTION FOR INFUSION 500MG/100ML	CHEMILL PHARMA LIMITED	HK-66809	Unknown
1408		METRONIDAZOLE CAP 200MG	NEOCHEM PHARMACEUTICAL LABORATORIES LTD.	HK-33041	Unknown
1409		METRONIDAZOLE CAP 200MG	VICKMANS LABORATORIES LTD	HK-36789	No
1410		METRONIDAZOLE INJ 0.5%	B. BRAUN MEDICAL (HK) LTD	HK-28023	No
1411		METRONIDAZOLE INJ 5MG/ML	PFIZER CORPORATION HONG KONG LIMITED	HK-31092	Unknown
1412		METRONIDAZOLE IV INFUSION 0.5% (BAXTER)	BAXTER HEALTHCARE LTD	HK-40240	Unknown
1413		METRONIDAZOLE TAB 200MG	NEOCHEM PHARMACEUTICAL LABORATORIES LTD.	HK-19402	Unknown
1414		METRONIDAZOLE TAB 200MG	VAST RESOURCES PHARMACEUTICAL LTD	HK-61292	Unknown
1415		METRONIL CAP 200MG	VICKMANS LABORATORIES LTD	HK-34348	No
1416		METROZOLE CAP 200MG	VICKMANS LABORATORIES LTD	HK-50326	No
1417		METROZOLE TAB 200MG	VICKMANS LABORATORIES LTD	HK-38355	No
1418		NIZOLE TAB 200MG	HOVID LIMITED	HK-35636	Unknown
1419		PHARMANIAGA METRONIDAZOLE TAB 200MG	HEALTHCARE PHARMASCIENCE LIMITED	HK-52934	Unknown
1420		SYNEODALIN TAB 200MG	NEOCHEM PHARMACEUTICAL LABORATORIES LTD.	HK-28848	Unknown
1421		TICOMONA-200 TAB 200MG	JEAN-MARIE PHARMACAL CO LTD	HK-21153	No
1422		TOLIZOLE CAPSULES 250MG	HITPHARM PHARMACEUTICAL CO LTD	HK-65308	Unknown
1423		TRICHAZOLE-200 TAB 200MG	JULIUS CHEN & CO (HK) LTD	HK-38685	Unknown
1424		VAGICIN TAB 200MG	EUROPHARM LAB CO LTD	HK-31381	No
1425	**PHENYTOIN**	DILANTIN CAP 100MG	VIATRIS HEALTHCARE HONG KONG LIMITED	HK-07909	Unknown
1426		DILANTIN INJ 250MG/5ML	VIATRIS HEALTHCARE HONG KONG LIMITED	HK-19932	Unknown
1427		DILANTIN SUSP 125MG/5ML	VIATRIS HEALTHCARE HONG KONG LIMITED	HK-07913	Unknown
1428		PHENYTOIN INJ 250MG/5ML	PFIZER CORPORATION HONG KONG LIMITED	HK-47301	Unknown
1429	**ETORICOXIB**	APTOXIA TABLETS 120MG	SYNCO (H.K.) LIMITED	HK-65902	Yes
1430		ARCOXIA TAB 120MG	ORGANON HONG KONG LIMITED	HK-51226	Unknown
1431		EBERIL 120 TABLETS 120MG	HEALTH ALLIANCE INTERNATIONAL CO LTD	HK-66215	Yes
1432		ETORICOXIB ALVOGEN TABLETS 30 MG	LOTUS PHARMACEUTICAL HK LIMITED	HK-65439	No
1433		ETORICOXIB FARMOZ TABLETS 120MG	TRENTON-BOMA LTD	HK-66070	No
1434		ETORICOXIB MYLAN TABLETS 120MG	MYLAN PHARMACEUTICAL HONG KONG LIMITED	HK-66666	Unknown
1435		ETORICOXIB STADA TABLETS 120MG	HONG KONG MEDICAL SUPPLIES LTD	HK-66165	No
1436		ETORICOXIB TEVA TABLETS 120MG	TEVA PHARMACEUTICAL HONG KONG O/B TEVA PHARMACEUTICAL HONG KONG LIMITED	HK-66582	Unknown
1437		ETOXIB TABLETS 120MG	SINO PACIFIC PHARMA COMPANY LIMITED	HK-65576	Unknown
1438		EUROXIA TABLETS 120MG	SYNCO (H.K.) LIMITED	HK-65907	Yes
1439		EXIB TABLETS 120MG	MEDILINE (HONG KONG) COMPANY LIMITED	HK-67058	Unknown
1440		FACOXIA TABLETS 120MG	DKSH HONG KONG LIMITED	HK-66798	Unknown
1441		JEANOXIA TABLETS 120MG	SYNCO (H.K.) LIMITED	HK-65960	Yes
1442		MEXIB 120 TABLETS 120MG	R. MANSTIEN (AUSTRALIA) LIMITED	HK-66451	Unknown
1443		RIBOX-120 TABLETS 120MG	EUGENPHARM INTERNATIONAL LIMITED	HK-65465	Unknown
1444		SYNCOXIA TABLETS 120MG	SYNCO (H.K.) LIMITED	HK-65784	Yes
1445		TEREXIB TABLETS 120MG	NOVARTIS PHARMACEUTICALS (HK) LIMITED	HK-64992	No
1446		VICKOXIA TABLETS 120MG	SYNCO (H.K.) LIMITED	HK-65905	Yes
1447	**CLARITHROMYCIN**	ACADIN TAB 250MG	JEAN-MARIE PHARMACAL CO LTD	HK-64683	Yes
1448		APT-CLARICID TABLETS 250MG	APT PHARMA LIMITED	HK-61870	Unknown
1449		AVEXUS TAB 250MG	FP HEALTHCARE LIMITED	HK-53836	Unknown
1450		BINOCLAR TAB 250MG	NOVARTIS PHARMACEUTICALS (HK) LIMITED	HK-49648	No
1451		CLACIN PLUS TAB 500MG	APT PHARMA LIMITED	HK-54294	Unknown
1452		CLACIN POWDER FOR ORAL SUSPENSION 125MG/5ML	APT PHARMA LIMITED	HK-54264	Unknown
1453		CLARAC TAB 250MG	UNITED ITALIAN CORP (HK) LTD	HK-55793	Yes
1454		CLAREM TABLETS 250MG	HEALTHCARE PHARMASCIENCE LIMITED	HK-66855	Unknown
1455		CLARICIN TABLETS 250MG	JCS PHARMACEUTICAL LTD	HK-64813	Unknown
1456		CLARIE 250 TAB 250MG	UNIPHARM (HONG KONG) LIMITED	HK-56751	Unknown
1457		CLARITH 250 TAB 250MG	NATURAL HEALTH RESOURCES COMPANY LIMITED	HK-56531	Yes
1458		CLARITHROMYCIN POWDER FOR CONCENTRATE FOR SOLUTION FOR INFUSION 500MG	THE INTERNATIONAL MEDICAL COMPANY LIMITED	HK-66605	Unknown
1459		CLARITHROMYCIN STELLA TABLETS 250MG	HONG KONG MEDICAL SUPPLIES LTD	HK-67144	No
1460		CLARITRON TAB 500MG	CHEMILLENNIUM INTERNATIONAL (HK) LIMITED	HK-60240	Unknown
1461		CLARITROX 500 TAB 500MG	KAI YUEN PHARMACEUTICAL CO	HK-54164	Unknown
1462		CLERON 250 TAB 250MG	DCH AURIGA (HONG KONG) LIMITED - UNIVERSAL DIVISION	HK-51079	Unknown
1463		JMP CLARITHROMYCIN TAB 250MG	JEAN-MARIE PHARMACAL CO LTD	HK-52825	Yes
1464		KARICIN TAB 250MG	EUROPHARM LAB CO LTD	HK-52581	Yes
1465		KLACID FORTE TAB 500MG	ABBOTT LAB LTD	HK-43890	No
1466		KLACID GRANULES FOR ORAL SUSP 125MG/5ML	ABBOTT LAB LTD	HK-36468	No
1467		KLACID IV FOR INJ 500MG/VIAL	ABBOTT LAB LTD	HK-38143	No
1468		KLACID TAB 250MG	ABBOTT LAB LTD	HK-35866	No
1469		KLAR 250 TABLETS 250MG	HANG LUNG TRADING (H.K.) CO	HK-66974	Unknown
1470		KLARITH TABLETS 250MG	CONTROLLED MEDICATIONS LTD.	HK-65132	Unknown
1471		KLERIMED TAB 250MG	STAR MEDICAL SUPPLIES LTD	HK-49963	No
1472		KORUS-CLARITHROMYCIN TAB 250MG	LSB (HK) LIMITED	HK-61270	Unknown
1473		MAXILIN POWDER FOR SOLUTION FOR INFUSION 500MG	HONG KONG MEDICAL SUPPLIES LTD	HK-65860	No
1474		MAXILIN TABLETS 500MG	HONG KONG MEDICAL SUPPLIES LTD	HK-65623	No
1475		MST -CLARITHROMYCIN TAB 250MG	HONG KONG MEDICAL SUPPLIES LTD	HK-51060	No
1476		PHARMANIAGA CLARITHROMYCIN TAB 250MG	HEALTHCARE PHARMASCIENCE LIMITED	HK-52623	Unknown
1477		ROLACIN 500 TAB 500MG	EUGENPHARM INTERNATIONAL LIMITED	HK-55605	Unknown
1478		SYNCLAR-250 TAB 250MG	CONTROLLED MEDICATIONS LTD.	HK-49863	Unknown
1479		VICK-CLARCID TAB 250MG	VICKMANS LABORATORIES LTD	HK-54399	No

1480	**NAPROXEN**	ALEVE TAB 220 MG	BAYER HEALTHCARE LIMITED	HK-51278	Unknown
1481		APO-NAPRO-NA TAB 275MG	HIND WING CO LTD	HK-44411	Unknown
1482		CP-NAPROXEN TAB 250MG	CHRISTO PHARM LTD	HK-47306	Yes
1483		INZA 250 TAB 250MG	LUEN CHEONG HONG LTD	HK-33326	Unknown
1484		NAPOSIN TABLETS 250MG	HITPHARM PHARMACEUTICAL CO LTD	HK-64205	Unknown
1485		NAPROREX 250 TAB 250MG	DCH AURIGA (HONG KONG) LIMITED - UNIVERSAL DIVISION	HK-41617	Unknown
1486		NAPROXEN TAB 250MG	TEVA PHARMACEUTICAL HONG KONG O/B TEVA PHARMACEUTICAL HONG KONG LIMITED	HK-35500	Unknown
1487		NAPROXEN TAB 250MG	UNITED ITALIAN CORP (HK) LTD	HK-50090	No
1488		NAPROXEN TAB 250MG	VICKMANS LABORATORIES LTD	HK-51733	No
1489		NAPROXEN TAB 250MG (SINPHAR)	SUNTOL MEDICAL LIMITED	HK-48288	Unknown
1490		NAPROXEN TABLETS 250MG	PRUDENTLINK LIMITED	HK-65797	Unknown
1491		NAPXEN TAB 250MG	APT PHARMA LIMITED	HK-44905	Unknown
1492		POINT TAB 275MG	GAILY PHARMACEUTICAL COMPANY LIMITED	HK-52304	Unknown
1493		SAFROSYN S TABLET 275MG	HEALTHCARE PHARMASCIENCE LIMITED	HK-61799	Unknown
1494		SINTON TAB 375MG	HITPHARM PHARMACEUTICAL CO LTD	HK-49259	Unknown
1495		SNOFIN TABLETS 275MG	LSB (HK) LIMITED	HK-62355	Unknown
1496		SODEN TAB 275MG	TEVA PHARMACEUTICAL HONG KONG O/B TEVA PHARMACEUTICAL HONG KONG LIMITED	HK-45671	Unknown
1497		SOREN TAB 275MG	HEALTHCARE PHARMASCIENCE LIMITED	HK-43951	Unknown
1498		SYN-NAPROXEN TAB 250MG	SYNCO (H.K.) LIMITED	HK-56618	No
1499	**CHLORAMPHENICOL**	ANLINA VAGINAL TAB	DELTAPHARM LIMITED	HK-43469	Unknown
1500		CHLOTIN VAGINAL SUPP	YUNG SHIN CO LTD	HK-06879	Unknown
1501	**CELECOXIB**	APO-CELECOXIB CAPSULES 100MG	HIND WING CO LTD	HK-64176	Unknown
1502		APT-CELECOXIB CAPSULES 100MG	EUROPHARM LAB CO LTD	HK-64340	No
1503		CECOLEX CAPSULES 200MG	SUNTOL MEDICAL LIMITED	HK-67125	Unknown
1504		CELCOX CAP 100MG	GAILY PHARMACEUTICAL COMPANY LIMITED	HK-52808	Unknown
1505		CELCOXX CAP 100MG	CHARIOT PHARMA LIMITED	HK-56855	Unknown
1506		CELEBREX CAP 100MG	VIATRIS HEALTHCARE HONG KONG LIMITED	HK-44730	Unknown
1507		CELECOLEN CAPSULES 200MG	WILCOME PHARMACEUTICAL CO LTD	HK-65035	Unknown
1508		CELECOXIB CAPSULES 200MG	JULIUS CHEN & CO (HK) LTD	HK-64895	Unknown
1509		CELECOXIB CAPSULES 200MG	MEYER BPC PHARMACEUTICALS LIMITED	HK-65678	Unknown
1510		CELECOXIB CAPSULES 200MG	R. MANSTIEN (AUSTRALIA) LIMITED	HK-66325	Unknown
1511		CELECOXIB CAPSULES 200MG	CONTROLLED MEDICATIONS LTD.	HK-66388	Unknown
1512		CELECOXIB FARMOZ CAPSULES 100MG	TRENTON-BOMA LTD	HK-65135	No
1513		CELECOXIB SANDOZ CAPSULES 100MG	NOVARTIS PHARMACEUTICALS (HK) LIMITED	HK-61704	No
1514		CELXIB 200 CAPSULES 200MG	HEALTH ALLIANCE INTERNATIONAL CO LTD	HK-66622	No
1515		CENEX CAPSULES 100MG	STAR MEDICAL SUPPLIES LTD	HK-66327	Yes
1516		COBIX-200 CAP 200MG	CONTROLLED MEDICATIONS LTD.	HK-58909	Unknown
1517		ECOPAIN CAPSULES 200MG	YUNG SHIN CO LTD	HK-67240	Unknown
1518		EUROBREX CAPSULES 100MG	EUROPHARM LAB CO LTD	HK-64343	No
1519		FAVOCOX CAP 200MG	DKSH HONG KONG LIMITED	HK-60270	Unknown
1520		HOVID CELECOXIB CAPSULES 200MG	HOVID LIMITED	HK-67377	Unknown
1521		KELTREX CAPSULES 100MG	LSB (HK) LIMITED	HK-66698	Unknown
1522		NUMED CELECOXIB CAPSULES 200MG	WAI FAT PHARMACEUTICAL COMPANY LIMITED	HK-66326	Unknown
1523		PINBREX CAPSULES 200MG	JULIUS CHEN & CO (HK) LTD	HK-66003	Unknown
1524		RELECOS CAPSULES 200MG	WELLDONE PHARMACEUTICALS LIMITED	HK-66237	Unknown
1525		STADLORIC 200 CAPSULES 200MG	HONG KONG MEDICAL SUPPLIES LTD	HK-64865	No
1526		SUMITA CAPSULES 200MG	WELLDONE PHARMACEUTICALS LIMITED	HK-66192	Unknown
1527		UNIBREX CAPSULES 100MG	EUROPHARM LAB CO LTD	HK-64341	No
1528		ZOBREX CAPSULES 200MG	NATURAL HEALTH RESOURCES COMPANY LIMITED	HK-66189	No
1529	**TRAMADOL**	ACUGESIC CAP 50MG	WINGS PHARMACEUTICAL LTD	HK-45547	No
1530		ACUGESIC INJ 100MG/2ML (AMPOULE)	WINGS PHARMACEUTICAL LTD	HK-45549	No
1531		ACUGESIC INJ 50MG/ML (VIAL)	WINGS PHARMACEUTICAL LTD	HK-45550	No
1532		ACUGESIC TAB 50MG	WINGS PHARMACEUTICAL LTD	HK-45548	No
1533		APO-TRAMADOL/ACET TAB 37.5/325MG	HIND WING CO LTD	HK-61588	Unknown
1534		EURO-TRAMADOL CAPSULES 50MG	JEAN-MARIE PHARMACAL CO LTD	HK-63928	No
1535		HYCEPHEN TABLET	LSB (HK) LIMITED	HK-61954	Unknown
1536		KORIDOL CAPSULES 50MG	LSB (HK) LIMITED	HK-61722	Unknown
1537		LUCIDOL CAP 50MG	EUGENPHARM INTERNATIONAL LIMITED	HK-55355	Unknown
1538		MABRON CAP 50MG	STAR MEDICAL SUPPLIES LTD	HK-35707	No
1539		MABRON INJ 100MG	STAR MEDICAL SUPPLIES LTD	HK-35130	No
1540		MYOTRAM 100 SOLUTION FOR INJECTION/INFUSION 100MG/2ML	HANG LUNG TRADING (H.K.) CO	HK-66387	Unknown
1541		PAINDOL CAPSULES 50MG	HEALTHCARE PHARMASCIENCE LIMITED	HK-65127	Unknown
1542		PENGESIC SR TAB 100MG	HOVID LIMITED	HK-59354	Unknown
1543		PINOREC CAPSULES 50MG	HIND WING CO LTD	HK-60872	Unknown
1544		SEFMAL CAP 50MG	HEALTH ALLIANCE INTERNATIONAL CO LTD	HK-44533	No
1545		SKUDEXA TABLETS 75MG/25MG	A. MENARINI HONG KONG LIMITED	HK-65491	Unknown
1546		SYN-TRAMADOL CAPSULE 50MG	JEAN-MARIE PHARMACAL CO LTD	HK-63427	No
1547		TAMADOL SOLUTION FOR INJECTION 50MG/ML	PRUDENTLINK LIMITED	HK-63103	Unknown
1548		TARACET TABLETS	PRUDENTLINK LIMITED	HK-62152	Unknown
1549		TRABILIN-100 INJ 100MG/2ML	CEUTICAL TRADING COMPANY LIMITED	HK-53386	Unknown
1550		TRADOLGESIC CAP 50MG	TRENTON-BOMA LTD	HK-58224	No
1551		TRADYL CAP 50MG	NATURAL HEALTH RESOURCES COMPANY LIMITED	HK-46375	No
1552		TRAMACET TABLETS	VAST RESOURCES PHARMACEUTICAL LTD	HK-64581	Unknown
1553		TRAMADA CAP 50MG	DELTAPHARM LIMITED	HK-55394	Unknown
1554		TRAMADOL 50 STADA CAP 50MG	STADA PHARMACEUTICALS (ASIA) LTD	HK-41704	Unknown
1555		TRAMADOL CINFA CAP 50MG	REICH PHARM LIMITED	HK-51696	Unknown
1556		TRAMADOL HYDROCHLORIDE CAPSULES 50MG	TEVA PHARMACEUTICAL HONG KONG O/B TEVA PHARMACEUTICAL HONG KONG LIMITED	HK-61553	Unknown
1557		TRAMADOL HYDROCHLORIDE TABLETS USP 50MG	CONTROLLED MEDICATIONS LTD.	HK-66984	Unknown
1558		TRAMADOL NORMON CAPSULES 50MG	HIND WING CO LTD	HK-61867	Unknown
1559		TRAMADOL SANDOZ CAPSULES 50MG	NOVARTIS PHARMACEUTICALS (HK) LIMITED	HK-61770	No
1560		TRAMADOL STADA DROPS 100MG/ML	STADA PHARMACEUTICALS (ASIA) LTD	HK-41706	Unknown
1561		TRAMADOL STADA INJ 100MG/2ML	STADA PHARMACEUTICALS (ASIA) LTD	HK-41705	Unknown
1562		TRAMADOL-HAMELN INJ 50 MG/ML	MEKIM LTD	HK-58629	Unknown
1563		TRAMO CAP 50MG	JEAN-MARIE PHARMACAL CO LTD	HK-38308	No
1564		TRASIC CAP 50MG	NIDOWAY INVESTMENT LTD	HK-42968	Unknown
1565		ULTRACET TAB	JOHNSON & JOHNSON (HONG KONG) LTD.	HK-50587	Unknown
1566		ULTRAN TABLET	JULIUS CHEN & CO (HK) LTD	HK-60822	Unknown
1567		UNI-TRAMADOL CAPSULES 50MG	JEAN-MARIE PHARMACAL CO LTD	HK-64086	No
1568		VOLCIDOL TAB 50MG	STAR MEDICAL SUPPLIES LTD	HK-61480	No
1569		WONTRAN TABLETS	HEALTHCARE PHARMASCIENCE LIMITED	HK-62389	Unknown
1570		ZIMACO TABLETS	LAFARGE CO., LIMITED	HK-65425	Unknown
1571	**KETOROLAC**	KETO ENTERIC-MICROENCAPSULATED CAP 10MG	YUNG SHIN CO LTD	HK-53935	Unknown
1572		KETO INJ 30MG/ML	YUNG SHIN CO LTD	HK-49503	Unknown
1573		KETOROLAC KABI SOLUTION FOR INJECTION 30MG/ML	FRESENIUS KABI HONG KONG LIMITED	HK-66788	Unknown
1574		KETOROLAC TROMETAMOL NORMON INJ SOLN 30MG/ML	HIND WING CO LTD	HK-60656	Unknown
1575		KETRO TABLET 10MG	JULIUS CHEN & CO (HK) LTD	HK-62136	Unknown
1576		TORADOL INJ 3%	DKSH HONG KONG LIMITED	HK-35309	Unknown
1577	**HYOSCINE**	AIRMIT TAB (TAIWAN)	SATO PHARMACEUTICAL (HK) CO LTD	HK-60329	Unknown
1578		ANTISPA SOLUTION FOR INJECTION 20MG/ML	DELTAPHARM LIMITED	HK-64350	Unknown
1579		BF-HORLOTEN TAB 10MG	BRIGHT FUTURE PHARMACEUTICALS FACTORY O/B BRIGHT FUTURE PHARMACEUTICAL LABORATORIES LIMITED	HK-48396	Unknown
1580		BUSCO S.C. TAB 10MG	VAST RESOURCES PHARMACEUTICAL LTD	HK-52686	Unknown
1581		BUSCOPALAMIN SYRUP 5MG/5ML (GREEN)	NEOCHEM PHARMACEUTICAL LABORATORIES LTD.	HK-12413	Unknown
1582		BUSCOPALAMIN TAB	SYNCO (H.K.) LIMITED	HK-37251	No
1583		BUSCOPAN INJ 20MG/ML	SANOFI HONG KONG LIMITED	HK-02712	Unknown
1584		BUSCOPAN TAB 10MG	SANOFI HONG KONG LIMITED	HK-02721	Unknown
1585		BUSOPIN SYRUP 5MG/5ML	JEAN-MARIE PHARMACAL CO LTD	HK-21167	No
1586		BUSOPIN TAB 10MG	JEAN-MARIE PHARMACAL CO LTD	HK-08598	Yes
1587		BUSPAN TAB 10MG	STAR MEDICAL SUPPLIES LTD	HK-53879	No
1588		CENCOPAN S TAB 10MG	STAR MEDICAL SUPPLIES LTD	HK-60604	Yes
1589		COLOSPAN TAB 10MG	HOVID LIMITED	HK-35986	Unknown
1590		DETTACKS S.C. TAB 10MG	VAST RESOURCES PHARMACEUTICAL LTD	HK-60442	Unknown

1591		DHACOPAN SYRUP 5MG/5ML	TEVA PHARMACEUTICAL HONG KONG O/B TEVA PHARMACEUTICAL HONG KONG LIMITED	HK-45670	Unknown
1592		DHACOPAN TAB 10MG	TEVA PHARMACEUTICAL HONG KONG O/B TEVA PHARMACEUTICAL HONG KONG LIMITED	HK-31555	Unknown
1593		DIVIDOL TABLETS 10MG	HEALTHCARE PHARMASCIENCE LIMITED	HK-66755	Unknown
1594		EPILON TAB	YUNG SHIN CO LTD	HK-10161	Unknown
1595		EUROPON TAB 10MG	EUROPHARM LAB CO LTD	HK-42313	Yes
1596		FUCON INJ 20MG/ML	YUNG SHIN CO LTD	HK-46154	Unknown
1597		HOLOMINE TAB 1MG	SYNCO (H.K.) LIMITED	HK-50327	No
1598		HYOSCINE BUTYLBROMIDE	SUNRISE TRADING CO	HK-53021	Unknown
1599		HYOSCINE BUTYLBROMIDE INJ BP 20MG/ML	STAR MEDICAL SUPPLIES LTD	HK-55614	No
1600		HYOSCINE BUTYLBROMIDE TAB 10MG	ORIENTAL INT'L HEALTH PRODUCTS CO LTD	HK-43641	Unknown
1601		HYOSCINE METHOBROMIDE SYR 1MG/5ML	NEOCHEM PHARMACEUTICAL LABORATORIES LTD.	HK-35649	Unknown
1602		HYOSCINE SYRUP 5MG/5ML (VICKMANS)	VICKMANS LABORATORIES LTD	HK-39990	No
1603		HYOSCINE TAB 10MG	VICKMANS LABORATORIES LTD	HK-06333	No
1604		HYOSPAN TAB 10MG	NATURAL HEALTH RESOURCES COMPANY LIMITED	HK-47716	Yes
1605		HYSPAN INJ 20MG/ML	JULIUS CHEN & CO (HK) LTD	HK-50032	Unknown
1606		HYSPAN TAB 10MG	JULIUS CHEN & CO (HK) LTD	HK-49885	Unknown
1607		JECEFARMA HYOSCINE BUTYLBROMIDE TABLETS 10MG	JULIUS CHEN & CO (HK) LTD	HK-63916	Unknown
1608		L-SCOPOLAMINE-N-METHYLBROMIDE TAB 1MG	SYNCO (H.K.) LIMITED	HK-04978	No
1609		NEO BUSCO TAB 10MG	SYNCO (H.K.) LIMITED	HK-05433	No
1610		NEO-COPAN TAB 1MG	NICE LABORATORIES LTD	HK-11459	Unknown
1611		PHARMANIAGA HYOSCINE TAB 10MG	HEALTHCARE PHARMASCIENCE LIMITED	HK-55156	Unknown
1612		SCOLOPON TAB 1MG	SYNCO (H.K.) LIMITED	HK-47719	No
1613		SCOPALAMIN SYRUP 5MG/5ML	CHRISTO PHARM LTD	HK-27133	No
1614		SCOPAM S.C. TAB 10MG (JEN SHENG)	LANWAY LIMITED	HK-55061	No
1615		SCOPOLAMINE METHYLBROMIDE TAB 1MG	SYNCO (H.K.) LIMITED	HK-11452	No
1616		SCOPOLAMINE METHYLBROMIDE TAB 1MG	CHRISTO PHARM LTD	HK-37156	No
1617		SCOPOLAMINE SYRUP 1MG/5ML	NEOCHEM PHARMACEUTICAL LABORATORIES LTD.	HK-28940	Unknown
1618		SCOPOLAMINE-BUTYL BROMIDE TAB 10MG	CHRISTO PHARM LTD	HK-13372	No
1619		SPANI TABLETS 10MG	EUGENPHARM INTERNATIONAL LIMITED	HK-66236	Unknown
1620		SPASCOPAN TAB 10MG	TRENTON-BOMA LTD	HK-50644	No
1621		SPASMOLIV TAB 10MG	FP HEALTHCARE LIMITED	HK-29286	Unknown
1622		U-METAMINE TAB 1MG	SYNCO (H.K.) LIMITED	HK-33198	No
1623		UNI-SCOPOL SYRUP 5MG/5ML	UNIVERSAL PHARMACEUTICAL LABORATORIES, LIMITED	HK-37830	Unknown
1624		UNIGAN TAB	HEALTH ALLIANCE INTERNATIONAL CO LTD	HK-43954	Yes
1625		VESUTON TAB 10MG	HITPHARM PHARMACEUTICAL CO LTD	HK-50014	Unknown
1626	**PROCHLORPERAZINE**	METIL TAB 5MG	VICKMANS LABORATORIES LTD	HK-06335	No
1627		NAUTISOL INJ 12.5MG	STAR MEDICAL SUPPLIES LTD	HK-35835	No
1628		NAUTISOL TAB 5MG	STAR MEDICAL SUPPLIES LTD	HK-25480	No
1629		PEMAZIL TAB 5MG	SYNCO (H.K.) LIMITED	HK-07137	No
1630		PROCHLOR TABLETS 5MG	WAI LUN TRADING CO	HK-61940	Unknown
1631		PROCHLORPERAZINE MALEATE TAB 5MG	JEAN-MARIE PHARMACAL CO LTD	HK-01679	No
1632		PROCHLORPERAZINE MALEATE TABLETS 5MG	PRUDENTLINK LIMITED	HK-65834	Unknown
1633		PROCHLORPERAZINE TAB 5MG	SYNCO (H.K.) LIMITED	HK-07368	No
1634		PROCHLORPERAZINE TAB 5MG	NEOCHEM PHARMACEUTICAL LABORATORIES LTD.	HK-05448	Unknown
1635		PROCHLORPERAZINE TAB 5MG	TEVA PHARMACEUTICAL HONG KONG O/B TEVA PHARMACEUTICAL HONG KONG LIMITED	HK-38810	Unknown
1636		PROTIL TAB 5MG	MEYER PHARMACEUTICALS LTD	HK-08162	Unknown
1637		SERATIL TAB 5MG	CHRISTO PHARM LTD	HK-21027	No
1638		STEMETIL TAB 5MG	SANOFI HONG KONG LIMITED	HK-31117	Unknown
1639	**METOCLOPRAMIDE**	BALON S.C. TAB 5MG	VAST RESOURCES PHARMACEUTICAL LTD	HK-52495	Unknown
1640		CHIAOWELGEN INJ 10MG/2ML	SWISS PHARMACEUTICAL CO. LIMITED	HK-17388	Unknown
1641		CHIAOWELGEN S C TAB 5MG	SWISS PHARMACEUTICAL CO. LIMITED	HK-17394	Unknown
1642		CLOPERAN 10 TABLETS 10MG	HEALTHCARE PHARMASCIENCE LIMITED	HK-64279	Unknown
1643		MARIL INJ 10MG/2ML	ATLANTIC PHARMACEUTICAL LIMITED	HK-47468	Unknown
1644		MARIL TAB 10MG	ATLANTIC PHARMACEUTICAL LIMITED	HK-47467	Unknown
1645		METOCLOPRAMIDE TAB 10MG	TEVA PHARMACEUTICAL HONG KONG O/B TEVA PHARMACEUTICAL HONG KONG LIMITED	HK-36811	Unknown
1646		METOCLOPRAMIDE TAB 5MG	NEOCHEM PHARMACEUTICAL LABORATORIES LTD.	HK-17905	Unknown
1647		METOLON TAB 10MG	HOVID LIMITED	HK-35582	Unknown
1648		METOMIDE TAB 10MG	CHRISTO PHARM LTD	HK-22284	No
1649		METOMIDE TAB 10MG	VICKMANS LABORATORIES LTD	HK-20751	No
1650		METRAM TAB 10MG	SYNCO (H.K.) LIMITED	HK-44619	No
1651		PIRALEN SYRUP 5MG/5ML	HANG LUNG TRADING (H.K.) CO	HK-55182	Unknown
1652		PRIMPERAN INJ 10MG/2ML	SANOFI HONG KONG LIMITED	HK-07173	Unknown
1653		PROMERAN INJ 3.84MG/ML	KAI YUEN PHARMACEUTICAL CO	HK-16825	Unknown
1654		SINPRIM F.C. TAB 10MG	HITPHARM PHARMACEUTICAL CO LTD	HK-49285	Unknown
1655		SYNTOMIDE SYRUP 5MG/5ML	SYNCO (H.K.) LIMITED	HK-37155	No
1656		SYNTOMIDE TAB 10MG	SYNCO (H.K.) LIMITED	HK-29060	No
1657		TABS MA-5 TAB 5MG	NEOCHEM PHARMACEUTICAL LABORATORIES LTD.	HK-21341	Unknown
1658	**OFLOXACIN**	AFLOCIN CAP 200MG	NEOCHEM PHARMACEUTICAL LABORATORIES LTD.	HK-50468	Unknown
1659		KORUS OFLOXACIN TAB 200MG	LSB (HK) LIMITED	HK-60966	Unknown
1660		NOVECIN TAB 400MG	AUSTRALIAN MEDIC-CARE COMPANY LTD	HK-61671	Unknown
1661		OFLOXACIN TAB 200MG	HEALTHCARE PHARMASCIENCE LIMITED	HK-52655	Unknown
1662		OFLOXACINA SOLN FOR INF 2MG/ML	TRENTON-BOMA LTD	HK-59923	No
1663		OFLOXAL CAP 200MG	EUROPHARM LAB CO LTD	HK-48900	No
1664		OFLOXAL TAB 200MG	EUROPHARM LAB CO LTD	HK-48423	Yes
1665		OXACIN F.C. TAB 100MG	HITPHARM PHARMACEUTICAL CO LTD	HK-49282	Unknown
1666		PUIRITOL TAB 100MG	US NANO FOOD AND DRUG (HK) LIMITED	HK-47706	Unknown
1667		SINFLO TABLETS 200MG	TOP HARVEST PHARMACEUTICALS COMPANY LIMITED	HK-64416	Unknown
1668		ZO 200 TAB 200MG	STAR MEDICAL SUPPLIES LTD	HK-50565	Yes
1669	**AZITHROMYCIN**	APO-AZITHROMYCIN TAB 250MG	HIND WING CO LTD	HK-57627	Unknown
1670		AZ-1 CAP 250MG	NIDOWAY INVESTMENT LTD	HK-43917	Unknown
1671		AZIBACT-250 TABLETS 250MG	SWEDISH TRADING COMPANY LIMITED	HK-62320	Unknown
1672		AZICINE CAP 250MG	HONG KONG MEDICAL SUPPLIES LTD	HK-51372	No
1673		AZIMAX-250 TABLET 250MG	HOVID LIMITED	HK-62519	Unknown
1674		AZIN 250 CAP 250MG	DELTAPHARM LIMITED	HK-53157	Unknown
1675		AZITHMAX TABLET 250MG	VICKMANS LABORATORIES LTD	HK-62597	No
1676		AZITHROCIN 500 TAB 500MG	EUGENPHARM INTERNATIONAL LIMITED	HK-55422	Unknown
1677		AZITHROCIN CAP 250MG	EUGENPHARM INTERNATIONAL LIMITED	HK-55606	Unknown
1678		AZITHROMYCIN 250 - 1A PHARMA TAB 250MG	NOVARTIS PHARMACEUTICALS (HK) LIMITED	HK-59814	Yes
1679		AZITHROMYCIN HEC TABLETS 250MG	CEUTICAL TRADING COMPANY LIMITED	HK-64945	Unknown
1680		AZITROMICINA FARMOZ FOR INJ 500MG	TRENTON-BOMA LTD	HK-59530	No
1681		AZITROMICINA FARMOZ TAB 500MG (SOFARIMEX)	TRENTON-BOMA LTD	HK-60110	Yes
1682		AZITROMICINA FARMOZ TAB 500MG (WEST PHARMA)	TRENTON-BOMA LTD	HK-60111	Yes
1683		AZTRIN 250 CAP 250MG	VIEWBEST HOLDINGS LIMITED	HK-56104	Unknown
1684		BINOZYT POWDER FOR SUSP 200MG/5ML	NOVARTIS PHARMACEUTICALS (HK) LIMITED	HK-59622	No
1685		CLINDAL AZ TAB 500MG	APT PHARMA LIMITED	HK-49916	Unknown
1686		EUZIMAX TABLETS 250MG	VICKMANS LABORATORIES LTD	HK-62396	No
1687		FLOCTIL CAP 250MG	HEALTH ALLIANCE INTERNATIONAL CO LTD	HK-60123	No
1688		IMEXA FILM-COATED TABLETS 250MG	FP HEALTHCARE LIMITED	HK-62024	Unknown
1689		IMEXA POWDER FOR ORAL SUSPENSION 200MG/5ML	FP HEALTHCARE LIMITED	HK-62503	Unknown
1690		MARZOMAX TABLETS 250MG	VICKMANS LABORATORIES LTD	HK-62397	No
1691		NIFOMAX TABLETS 250MG	VICKMANS LABORATORIES LTD	HK-62598	No
1692		SUMAMED CAP 250MG	LEE'S PHARMACEUTICAL (H.K) LIMITED	HK-51610	Unknown
1693		SUMAMED TAB 500MG	LEE'S PHARMACEUTICAL (H.K) LIMITED	HK-51888	Unknown
1694		SYN-AZITHROMYCIN TABLETS 250MG	VICKMANS LABORATORIES LTD	HK-62600	No
1695		VICK-AZITHRO TABLET 250MG	VICKMANS LABORATORIES LTD	HK-62599	No
1696		ZAROM 250 CAP 250MG	PERFECT GROUPS LTD	HK-42363	Unknown
1697		ZATHRIN 250 TAB 250MG	STAR MEDICAL SUPPLIES LTD	HK-51586	Yes
1698		ZETRO CAP 250MG	CHARIOT PHARMA LIMITED	HK-59293	Unknown
1699		ZIMAX 500 TAB 500MG	UNIPHARM (HONG KONG) LIMITED	HK-57780	Unknown
1700		ZITHRIN TABLET 250MG	HEALTHCARE PHARMASCIENCE LIMITED	HK-61760	Unknown
1701		ZITHROMAX FOR INJ IV 500MG/VIAL	PFIZER CORPORATION HONG KONG LIMITED	HK-45614	Unknown
1702		ZITHROMAX POWDER FOR ORAL SUSP 200MG/5ML	PFIZER CORPORATION HONG KONG LIMITED	HK-36432	Unknown
1703		ZITHROMAX TAB 250MG	PFIZER CORPORATION HONG KONG LIMITED	HK-44360	Unknown

1704		ZITHROTEL POWDER FOR SOLUTION FOR INFUSION 500MG	HONG KONG MEDICAL SUPPLIES LTD	HK-65743	No
1705		ZITHROTEL TABLETS 250MG	HONG KONG MEDICAL SUPPLIES LTD	HK-65665	No
1706		ZYCIN 500 TAB 500MG	NATURAL HEALTH RESOURCES COMPANY LIMITED	HK-59803	Yes
1707		ZYCIN CAP 250MG	NATURAL HEALTH RESOURCES COMPANY LIMITED	HK-47682	No
1708	**VANCOMYCIN**	CELOVAN FOR INJ 500MG	LUEN CHEONG HONG LTD	HK-60209	Unknown
1709		VANCOCIN CP FOR INJ 500MG IV	A. MENARINI HONG KONG LIMITED	HK-20495	Unknown
1710		VANCOMYCIN HCL FOR INJ 1G IV	PFIZER CORPORATION HONG KONG LIMITED	HK-33908	Unknown
1711		VANCOMYCIN HCL FOR INJ 500MG IV	PFIZER CORPORATION HONG KONG LIMITED	HK-33907	Unknown
1712		VANCOMYCIN KABI POWDER FOR CONCENTRATE FOR SOLUTION FOR INFUSION 500MG	FRESENIUS KABI HONG KONG LIMITED	HK-64106	Unknown
1713		VANCOMYCIN POWDER FOR SOLUTION FOR INFUSION 500MG	HONG KONG MEDICAL SUPPLIES LTD	HK-63663	No
1714		VONDEM POWDER FOR CONCENTRATE FOR SOLUTION FOR INFUSION 500MG	HIND WING CO LTD	HK-65454	Unknown
1715	**CEFTRIAXONE**	BROADCED FOR INJ 1G	HANG LUNG TRADING (H.K.) CO	HK-55267	Unknown
1716		CEF-3 INJ IM 1G	KAI YUEN PHARMACEUTICAL CO	HK-58306	Unknown
1717		CEFAX FOR INJ 500MG	JULIUS CHEN & CO (HK) LTD	HK-57414	Unknown
1718		CEFIN FOR I.V. INJ 0.25G	YAT SENG TRADING CO	HK-47177	Unknown
1719		CEFIN FOR INJ 1G	YAT SENG TRADING CO	HK-49630	Unknown
1720		CEFTRIAXONE 1G FOR INJ AND DEXTROSE INJ	B. BRAUN MEDICAL (HK) LTD	HK-59631	No
1721		CEFTRIAXONE POWDER FOR SOLUTION FOR INJ 1G	CEUTICAL TRADING COMPANY LIMITED	HK-61060	Unknown
1722		CEFTRIAXONE SODIUM FOR INJ 1G-UNITED LAB	THE UNITED LABORATORIES LTD	HK-51901	Unknown
1723		CEFTRIAXONE SODIUM STERILE	DAWNRAYS PHARMA (HONG KONG) LTD	HK-58116	Unknown
1724		CEFXONE FOR INTRAVENOUS INJ 1G	MAIN LIFE CORP LTD	HK-56661	Unknown
1725		KORIXAL POWDER FOR SOLUTION FOR INJECTION 0.5G	LSB (HK) LIMITED	HK-64250	Unknown
1726		MEDAXONUM FOR 1M INJ 1G (WITH SOLVENT)	STAR MEDICAL SUPPLIES LTD	HK-49795	No
1727		MEDAXONUM INJ 250MG	STAR MEDICAL SUPPLIES LTD	HK-56644	No
1728		MESPORIN-500 FOR IM INJ	CEUTICAL TRADING COMPANY LIMITED	HK-47013	Unknown
1729		MILLOSEF POWDER AND SOLVENT FOR SOLUTION FOR INJECTION 1G	MEDILINE (HONG KONG) COMPANY LIMITED	HK-61566	Unknown
1730		ROCEPHIN FOR INJ 1G IM STERILE POWDER	ROCHE HONG KONG LIMITED	HK-20192	No
1731		ROCEPHIN FOR INJ 1G IV STERILE POWDER	ROCHE HONG KONG LIMITED	HK-20190	No
1732		ROCEPHIN FOR INJ 250MG IM STERILE POWDER	ROCHE HONG KONG LIMITED	HK-20182	No
1733		ROCEPHIN FOR INJ 2G STERILE PDR FOR INF	ROCHE HONG KONG LIMITED	HK-20195	No
1734		ROCEPHIN FOR INJ 500MG IM STERILE POWDER	ROCHE HONG KONG LIMITED	HK-20187	No
1735		ROCEPHIN FOR INJ 500MG IV STERILE POWDER	ROCHE HONG KONG LIMITED	HK-20188	No
1736		ROCEPHIN POWDER & SOLVENT FOR INJ 250MG IV	ROCHE HONG KONG LIMITED	HK-52844	No
1737		SIACEF POWDER AND SOLVENT FOR SOLUTION FOR INJECTION 500MG	KAI YUEN PHARMACEUTICAL LIMITED	HK-66923	Unknown
1738		UNOCEF INJ 1000MG	WINGS PHARMACEUTICAL LTD	HK-58898	No
1739	**PIPERACILLIN + TAZOBACTAM**	ALVOTAZ POWDER FOR SOLUTION FOR INJECTION 4/0.5G	HONG KONG MEDICAL SUPPLIES LTD	HK-64548	Unknown
1740		AUROTAZ-P 2.25 FOR INJ 2.25GM	AUROBINDO PHARMA LIMITED	HK-58457	No
1741		AUROTAZ-P 4.5 FOR INJ 4.5GM	AUROBINDO PHARMA LIMITED	HK-58458	No
1742		PIPERACILLIN AND TAZOBACTAM POWDER FOR SOLUTION FOR INFUSION 4.5G	JINDUN PHARMA (H.K.) LIMITED	HK-66929	Unknown
1743		PIPERACILLIN SODIUM AND TAZOBACTAM SODIUM FOR I.V. INJ 4.5G	JINDUN PHARMA (H.K.) LIMITED	HK-57127	Unknown
1744		PIPERACILLIN SODIUM FOR INJ. 4G	JINDUN PHARMA (H.K.) LIMITED	HK-56958	Unknown
1745		PIPERACILLIN/TAZOBACTAM KABI POWDER FOR SOLUTION FOR INFUSION 2G/0.25G	FRESENIUS KABI HONG KONG LIMITED	HK-65421	No
1746		PIPERACILLIN/TAZOBACTAM KABI POWDER FOR SOLUTION FOR INFUSION 4G/0.5G	FRESENIUS KABI HONG KONG LIMITED	HK-65419	No
1747		PIPERACILLIN/TAZOBACTAM SANDOZ POWDER FOR SOLUTION FOR INFUSION 4.5G	SANDOZ HONG KONG LIMITED	HK-64861	No
1748		PITAMYCIN FOR INJ 2G	YUNG SHIN CO LTD	HK-57325	Unknown
1749		PYBACTAM 4.5G POWDER FOR SOLN FOR INJ/INF	SANDOZ HONG KONG LIMITED	HK-60314	No
1750		TAZOBACTAM/PIPERACILLIN SOD F/ INJ 2.25G (ZHUHAI UNITED LAB)	THE UNITED LABORATORIES LTD	HK-52054	Unknown
1751		TAZOCIN FOR INJ 4.5G	PFIZER CORPORATION HONG KONG LIMITED	HK-49703	No
1752		TAZOCIN FOR INJ 4.5G	PFIZER CORPORATION HONG KONG LIMITED	HK-58094	No
1753		TAZOREX POWDER FOR SOLUTION FOR INFUSION 4.5G/VIAL	HIND WING CO LTD	HK-62632	No
1754		TAZPEN POWDER FOR SOLUTION FOR INJECTION 4.5G	LUEN CHEONG HONG LTD	HK-61520	No
1755	**DOXYCYCLINE**	AMERMYCIN CAP 100MG	HEALTH ALLIANCE INTERNATIONAL CO LTD	HK-50372	No
1756		APO-DOXY CAP 100MG	HIND WING CO LTD	HK-34988	Unknown
1757		APO-DOXY-TAB 100MG	HIND WING CO LTD	HK-52492	Unknown
1758		BRONMYCIN CAP 100MG	GREAT EASTERN HEALTHCARE LTD.	HK-28867	Unknown
1759		DOXITAB TAB 100MG	APT PHARMA LIMITED	HK-44649	Unknown
1760		DOXY CAP 100MG	CHRISTO PHARM LTD	HK-42036	No
1761		DOXY CAP 100MG	HEALTHCARE PHARMASCIENCE LIMITED	HK-38107	Unknown
1762		DOXYCAP CAP 100MG	HOVID LIMITED	HK-38125	Unknown
1763		DOXYCILLIN CAP 100MG	VAST RESOURCES PHARMACEUTICAL LTD	HK-54833	Unknown
1764		DOXYCILLIN TAB 100MG	VAST RESOURCES PHARMACEUTICAL LTD	HK-55715	Unknown
1765		DOXYCYCLINE CAP 100MG	NEOCHEM PHARMACEUTICAL LABORATORIES LTD.	HK-18626	Unknown
1766		DOXYCYCLINE CAP 100MG	VICKMANS LABORATORIES LTD	HK-18625	No
1767		DOXYCYCLINE HYCLATE	SUNRISE TRADING CO	HK-61981	Unknown
1768		DOXYCYCLINE TAB 100MG	JEAN-MARIE PHARMACAL CO LTD	HK-59614	No
1769		DOXYMYCIN - 100 CAP 100MG	WAI LUN TRADING CO	HK-59635	Unknown
1770		DOXYNIN CAP 100MG	WILCOME PHARMACEUTICAL CO LTD	HK-60107	Unknown
1771		MEDOMYCIN CAP 100MG	STAR MEDICAL SUPPLIES LTD	HK-35035	No
1772		PHARMANIAGA DOXYCYCLINE CAPSULES 100MG	HEALTHCARE PHARMASCIENCE LIMITED	HK-64152	Unknown
1773		POLICYCLINE 100 CAP 100MG	NATURAL HEALTH RESOURCES COMPANY LIMITED	HK-54502	No
1774		REMYCIN 100 CAP 100MG	HEALTHCARE PHARMASCIENCE LIMITED	HK-44279	Unknown
1775		SYNVIBRA-100 CAP	VICKMANS LABORATORIES LTD	HK-37163	No
1776		TOLEXINE 50 TAB 50MG	CNW FAR EAST LIMITED	HK-42754	Unknown
1777		U-M TAB 100MG	NEOCHEM PHARMACEUTICAL LABORATORIES LTD.	HK-33605	Unknown
1778		VIBRAMYCIN FILM COATED TAB 100MG	PFIZER CORPORATION HONG KONG LIMITED	HK-05038	Unknown
1779		VICK-DOXYCYCLINE CAP 100MG	VICKMANS LABORATORIES LTD	HK-53705	No
1780	**NIFEDIPINE**	ADALAT GITS TAB 30MG	BAYER HEALTHCARE LIMITED	HK-37008	Unknown
1781		APO-NIFED CAP 10MG	HIND WING CO LTD	HK-39896	Unknown
1782		CORDIPIN RETARD TAB 20MG	UNIPHARM (HONG KONG) LIMITED	HK-46471	Unknown
1783		FENAMON SR TAB 20MG	MEDOCHEMIE (HONG KONG) LIMITED	HK-40799	Unknown
1784		NIFECARD XL TAB 30MG	NOVARTIS PHARMACEUTICALS (HK) LIMITED	HK-49884	Yes
1785		NIFEDI-DENK 10 RETARD 10MG	STAR MEDICAL SUPPLIES LTD	HK-38941	Yes
1786		NIFELAT RETARD TAB 20MG (SUSTAINED-REL)	CONTROLLED MEDICATIONS LTD.	HK-51195	Unknown
1787		VASDALAT RETARD TABLET 20MG	HANG LUNG TRADING (H.K.) CO	HK-41532	Unknown
1788	**LISINOPRIL**	APO-LISINOPRIL (TYPE Z) TAB 5MG	HIND WING CO LTD	HK-47577	Unknown
1789		APO-LISINOPRIL/HCTZ TAB 20/12.5MG	HIND WING CO LTD	HK-59725	Unknown
1790		BF-LISINOPRIL TABLETS 10MG	BRIGHT FUTURE PHARMACEUTICALS FACTORY O/B BRIGHT FUTURE PHARMACEUTICAL LABORATORIES LIMITED	HK-63565	Unknown
1791		DAPRIL TAB 10MG	STAR MEDICAL SUPPLIES LTD	HK-51882	No
1792		INTERPRIL 10 TAB 10MG	NATURAL HEALTH RESOURCES COMPANY LIMITED	HK-53846	No
1793		LISINOPRIL ACTAVIS TABLETS 10MG	TEVA PHARMACEUTICAL HONG KONG O/B TEVA PHARMACEUTICAL HONG KONG LIMITED	HK-64627	Unknown
1794		LISINOPRIL STADA TAB 10MG	HONG KONG MEDICAL SUPPLIES LTD	HK-47999	No
1795		LISIPRIL TAB 10MG	APT PHARMA LIMITED	HK-54319	Unknown
1796		LISORIL-10 TABLETS 10MG	SWEDISH TRADING COMPANY LIMITED	HK-62158	Unknown
1797		LIZNORIL TABLETS 10MG	APT PHARMA LIMITED	HK-67090	Unknown
1798		PERENAL TAB 20MG	JULIUS CHEN & CO (HK) LTD	HK-39998	Unknown
1799		PMS-LISINOPRIL TABLETS 10MG	TRENTON-BOMA LTD	HK-61775	Yes
1800		TRUPRIL TAB 10MG	CHARIOT PHARMA LIMITED	HK-56658	Unknown
1801		ZESTRIL TAB 10MG	ASTRAZENECA HONG KONG LTD	HK-30516	Unknown
1802	**CEFACLOR**	AXCEL CEFACLOR-250 CAPSULES 250MG	KOTRA PHARMA (HONG KONG) COMPANY	HK-64116	Unknown
1803		CASTAL FOR ORAL SUSPENSION 125MG/5ML	VICKMANS LABORATORIES LTD	HK-48943	No
1804		CECLOR CAP 250MG	A. MENARINI HONG KONG LIMITED	HK-20110	Unknown
1805		CECLOR FOR ORAL SUSP 125MG/5ML	A. MENARINI HONG KONG LIMITED	HK-20158	Unknown
1806		CECLOR MR TAB 375MG	A. MENARINI HONG KONG LIMITED	HK-37919	Unknown
1807		CEFACLOR 500 CAP 500MG	HOVID LIMITED	HK-56642	Unknown

1808		CEFALOR CAP 250MG	VICKMANS LABORATORIES LTD	HK-45153	No
1809		CLEANCEF CAP 250MG	JULIUS CHEN & CO (HK) LTD	HK-40129	Unknown
1810		CLORTRIN DRY SYRUP FOR SUSP 125MG/5ML	VICKMANS LABORATORIES LTD	HK-48837	No
1811		KORUS CEFACLOR CAPSULES 250MG	LSB (HK) LIMITED	HK-64431	Unknown
1812		MEDOCLOR CAP 250MG	STAR MEDICAL SUPPLIES LTD	HK-41396	No
1813		MEDOCLOR FORTE FOR SUSP 250MG/5ML	STAR MEDICAL SUPPLIES LTD	HK-41627	No
1814		MOCLE CAPSULES 250MG	LSB (HK) LIMITED	HK-62143	Unknown
1815		NEO-CLORA DRY SYRUP FOR SUSP 125MG/5ML	VICKMANS LABORATORIES LTD	HK-48690	No
1816		PHACLOR CAP 250MG	BRIGHT FUTURE PHARMACEUTICALS FACTORY O/B BRIGHT FUTURE PHARMACEUTICAL LABORATORIES LIMITED	HK-38652	Unknown
1817		SOFICLOR FOR ORAL SUSPENSION 125MG/5ML	FP HEALTHCARE LIMITED	HK-47851	Unknown
1818		SYNTOCLOR 250 CAP 250MG	CEUTICAL TRADING COMPANY LIMITED	HK-46041	Unknown
1819		SYNTOCLOR FOR SUSPENSION 125MG/5ML	CEUTICAL TRADING COMPANY LIMITED	HK-46887	Unknown
1820		VICKCLOR CAP 250MG	VICKMANS LABORATORIES LTD	HK-54415	No
1821	**AMLODIPINE**	A-PHINE TAB 5MG	HEALTHCARE PHARMASCIENCE LIMITED	HK-55432	Unknown
1822		ACERYCAL TAB 10MG/10MG	SERVIER HONG KONG LTD	HK-60215	No
1823		ACERYCAL TAB 5MG/5MG	SERVIER HONG KONG LTD	HK-60213	No
1824		ACTAPIN TAB 10MG	TEVA PHARMACEUTICAL HONG KONG O/B TEVA PHARMACEUTICAL HONG KONG LIMITED	HK-56475	Unknown
1825		AFITEN TABLETS 10MG	MEDOCHEMIE (HONG KONG) LIMITED	HK-66679	Unknown
1826		AFITEN TABLETS 5MG	MEDOCHEMIE (HONG KONG) LIMITED	HK-66680	Unknown
1827		ALOPINE TAB 5MG	VAST RESOURCES PHARMACEUTICAL LTD	HK-59321	Unknown
1828		AMCOPINE TAB 10MG	VAST RESOURCES PHARMACEUTICAL LTD	HK-57615	Unknown
1829		AMDIVASC TABLETS 10MG	EUROPHARM LAB CO LTD	HK-63419	No
1830		AMDOCAL-10 TAB 10MG	EUGENPHARM INTERNATIONAL LIMITED	HK-55424	Unknown
1831		AMDOL TAB 10MG	APT PHARMA LIMITED	HK-50602	Unknown
1832		AMEDIN 10 TAB 10MG	MEDREICH FAR EAST LIMITED	HK-53728	Unknown
1833		AMLO-DENK 5 TAB 5MG	STAR MEDICAL SUPPLIES LTD	HK-60787	No
1834		AMLOBIN TAB 5MG "STANDARD"	KAI YUEN PHARMACEUTICAL CO	HK-60212	Unknown
1835		AMLOCOR TABLETS 5MG	HEALTHCARE PHARMASCIENCE LIMITED	HK-65870	Unknown
1836		AMLOD 5 TAB 5MG	HEALTH ALLIANCE INTERNATIONAL CO LTD	HK-59056	No
1837		AMLODIGAMMA TAB 5MG	KERRY PHARMA (HONG KONG) LIMITED	HK-58208	Unknown
1838		AMLODIPINA FARMOZ TAB 10MG	TRENTON-BOMA LTD	HK-56592	No
1839		AMLODIPINE BESYLATE TAB 5MG (YANGTZE RIVER)	JINDUN PHARMA (H.K.) LIMITED	HK-58707	Unknown
1840		AMLODIPINE GP TABLETS 5MG	WINGS PHARMACEUTICAL LTD	HK-62982	No
1841		AMLODIPINE SANDOZ TAB 10MG	NOVARTIS PHARMACEUTICALS (HK) LIMITED	HK-60285	No
1842		AMLODIPINE TABLETS 10MG	I & C (HONG KONG) LIMITED	HK-61778	Unknown
1843		AMLODIPINE-TEVA TAB 5MG	TEVA PHARMACEUTICAL HONG KONG O/B TEVA PHARMACEUTICAL HONG KONG LIMITED	HK-56581	Unknown
1844		AMLODIPINE/VALSARTAN TEVA TABLETS 5MG/80MG	TEVA PHARMACEUTICAL HONG KONG O/B TEVA PHARMACEUTICAL HONG KONG LIMITED	HK-67204	Unknown
1845		AMLODIPINO CINFA TAB 10MG	REICH PHARM LIMITED	HK-58781	Unknown
1846		AMLOGARD TAB 5MG	VIATRIS HEALTHCARE HONG KONG LIMITED	HK-60476	Unknown
1847		AMLONG TABLETS 5MG	DCH AURIGA (HONG KONG) LIMITED - HEALTHCARE DIVISION	HK-66559	Unknown
1848		AMLOPIN 5 TAB 5MG	DELTAPHARM LIMITED	HK-52419	Unknown
1849		AMLOPRES-5 TAB 5MG	CONTROLLED MEDICATIONS LTD.	HK-51036	Unknown
1850		AMLORINE 5 TABLETS 5MG	HEALTHCARE PHARMASCIENCE LIMITED	HK-61303	Unknown
1851		AMLOTENS TAB 5MG	RICH PLAN INTERNATIONAL LTD	HK-58071	Unknown
1852		AMLOVAS TAB 10MG	HEALTHCARE PHARMA LIMITED	HK-54820	Unknown
1853		AMLOVASC TABLETS 5MG	NATURAL HEALTH RESOURCES COMPANY LIMITED	HK-66427	No
1854		AMLOZEN TAB 10MG	DAWNRAYS PHARMA (HONG KONG) LTD	HK-55812	Unknown
1855		AMNDILINE TAB 5MG	KAI YUEN PHARMACEUTICAL CO	HK-58119	Unknown
1856		AMODEP 5 TAB 5MG	STAR MEDICAL SUPPLIES LTD	HK-57510	No
1857		AMPIN TAB 5MG	YUNG SHIN CO LTD	HK-57952	Unknown
1858		AMTAS-10 TAB 10MG	JACOBSON MARKETING LIMITED	HK-56154	No
1859		AMVAS TABLETS 5MG	MEDILINE (HONG KONG) COMPANY LIMITED	HK-62574	Unknown
1860		ANLODIN TABLETS 5MG	YUNG SHIN CO LTD	HK-67239	Unknown
1861		APO-AMLODIPINE TAB 5MG	HIND WING CO LTD	HK-57468	Unknown
1862		APO-AMLODIPINE-ATORVASTATIN TABLETS 10MG/10MG	HIND WING CO LTD	HK-64574	Unknown
1863		AZOREN TAB 40MG/5MG	PFIZER CORPORATION HONG KONG LIMITED	HK-59800	Unknown
1864		BF-AMLODIPINE TABLETS 10MG	BRIGHT FUTURE PHARMACEUTICALS FACTORY O/B BRIGHT FUTURE PHARMACEUTICAL LABORATORIES LIMITED	HK-63331	Unknown
1865		BF-AMLODIPINE TABLETS 5MG	BRIGHT FUTURE PHARMACEUTICALS FACTORY O/B BRIGHT FUTURE PHARMACEUTICAL LABORATORIES LIMITED	HK-63330	Unknown
1866		CADUET TAB 10MG/10MG	VIATRIS HEALTHCARE HONG KONG LIMITED	HK-53721	Unknown
1867		CINOPEN TABLETS 5 MG	WILCOME PHARMACEUTICAL CO LTD	HK-65376	Unknown
1868		CODUWON TAB 5MG/10MG	HEALTHCARE PHARMASCIENCE LIMITED	HK-62028	Unknown
1869		CONCOR AMLO TABLETS 5MG/5MG	MERCK PHARMACEUTICAL (HK) LIMITED	HK-66322	Unknown
1870		CP-LOVAC 5 TAB 5MG	CHRISTO PHARM LTD	HK-54533	No
1871		DIP 10 TABLETS 10MG	NIDOWAY INVESTMENT LTD	HK-65155	Unknown
1872		DUOTRESTAR TABLETS 40MG/5MG	JACOBSON MARKETING LIMITED	HK-67052	No
1873		EXFORGE HCT TAB 10MG/160MG/12.5MG	NOVARTIS PHARMACEUTICALS (HK) LIMITED	HK-60139	Yes
1874		EXFORGE TAB 5MG/80MG (SPAIN)	NOVARTIS PHARMACEUTICALS (HK) LIMITED	HK-60452	Yes
1875		EXNORTAN TABLETS 10MG/160MG	ABBOTT LAB LTD	HK-67395	No
1876		HIFORGE TABLETS 5MG/160MG	SINO PACIFIC PHARMA COMPANY LIMITED	HK-65521	Unknown
1877		HOVASC TAB 5MG	HOVID LIMITED	HK-59080	Unknown
1878		HYPRESS TAB 5MG	UNIPHARM (HONG KONG) LIMITED	HK-53337	Unknown
1879		INTERVASK 5 TAB 5MG	NATURAL HEALTH RESOURCES COMPANY LIMITED	HK-57502	No
1880		KARDAM 5 TABLETS 5MG	AUROBINDO PHARMA LIMITED	HK-66897	Unknown
1881		LODIPINE TAB 5MG	VAST RESOURCES PHARMACEUTICAL LTD	HK-56533	Unknown
1882		LOFRAL-5 TAB 5MG	CEUTICAL TRADING COMPANY LIMITED	HK-55700	Unknown
1883		LOMAKLINE TABLETS 5MG	CHEMILLENNIUM INTERNATIONAL (HK) LIMITED	HK-60503	Unknown
1884		LOPICARD TAB 5MG	CHARIOT PHARMA LIMITED	HK-57879	Unknown
1885		NATRIXAM MODIFIED-RELEASE TABLETS 1.5MG/5MG	SERVIER HONG KONG LTD	HK-65207	Yes
1886		NIKP-AMLODIPINE/VALSARTAN TABLETS 5MG/80MG	DKSH HONG KONG LIMITED	HK-65240	Unknown
1887		NORMODIN TABLETS 5MG	FORTUNE NATIONAL (HONG KONG) LIMITED	HK-65451	Unknown
1888		NORVAN TABLETS 5MG/80MG	JACOBSON MARKETING LIMITED	HK-66378	Yes
1889		NORVASC TABLETS 2.5MG	VIATRIS HEALTHCARE HONG KONG LIMITED	HK-65946	Unknown
1890		PMS-AMLODIPINE TAB 5MG	TRENTON-BOMA LTD	HK-55770	No
1891		STADOVAS 10 TAB 10MG	HONG KONG MEDICAL SUPPLIES LTD	HK-58070	No
1892		STADOVAS 5 CAPSULES 5MG	HONG KONG MEDICAL SUPPLIES LTD	HK-67108	No
1893		STADOVAS 5 TAB 5MG	HONG KONG MEDICAL SUPPLIES LTD	HK-56532	No
1894		TRIPLIXAM TABLETS 5MG/1.25MG/5MG	SERVIER HONG KONG LTD	HK-65989	Yes
1895		TWYNSTA TAB 40MG/10MG	BOEHRINGER INGELHEIM (HK) LTD	HK-60511	Unknown
1896		UNASC TAB 5MG	YIK KWAN PHARMACEUTICALS CO LTD	HK-58703	Unknown
1897		VALSAM TABLETS 5MG/80MG	MEYER BPC PHARMACEUTICALS LIMITED	HK-65537	Unknown
1898		VIACORAM TABLETS 7MG/5MG	SERVIER HONG KONG LTD	HK-65793	No
1899		ZYNOR TAB 10MG	FP HEALTHCARE LIMITED	HK-57900	Unknown
1900	**SIMVASTATIN**	APO-SIMVASTATIN TAB 10MG	HIND WING CO LTD	HK-51718	Unknown
1901		AVASTINEE TAB 40MG	APT PHARMA LIMITED	HK-52397	Unknown
1902		BF-SIMVASTATIN TABLETS 20MG	BRIGHT FUTURE PHARMACEUTICALS FACTORY O/B BRIGHT FUTURE PHARMACEUTICAL LABORATORIES LIMITED	HK-64551	Unknown
1903		CHOLESTAT TAB 10MG	HANG LUNG TRADING (H.K.) CO	HK-50353	Unknown
1904		CHOLIB TABLETS 145MG/40MG	ABBOTT LAB LTD	HK-66375	No
1905		CORSTAT 20 TAB 20MG	MEDREICH FAR EAST LIMITED	HK-57389	Unknown
1906		COVASTIN TAB 40MG	FP HEALTHCARE LIMITED	HK-50289	Unknown
1907		EKERD SIMVASTATIN TABLETS 20MG	WILSON TRADING COMPANY LIMITED	HK-62003	Unknown
1908		JMP SIMVASTATIN TAB 20MG	JEAN-MARIE PHARMACAL CO LTD	HK-56763	Yes
1909		JUBSIMVAS TABLETS 10MG	CONTROLLED MEDICATIONS LTD.	HK-65133	Unknown
1910		KARDAK 80 TAB 80MG	AUROBINDO PHARMA LIMITED	HK-58984	Unknown
1911		LOCHOL TAB 20MG	TOP HARVEST PHARMACEUTICALS COMPANY LIMITED	HK-60556	Unknown
1912		LONQS SIM TABLETS 20MG	WILSON TRADING COMPANY LIMITED	HK-61922	Unknown
1913		PEAUSOIN SIMVASTATIN TAB 80MG	TREASURE MOUNTAIN DEVELOPMENT CO LTD	HK-58383	Unknown
1914		PHARMANIAGA SIMVASTATIN TAB 10MG	HEALTHCARE PHARMASCIENCE LIMITED	HK-52630	Unknown
1915		PMS-SIMVASTATIN TAB 80MG	TRENTON-BOMA LTD	HK-54530	No
1916		PRIACIN TABLETS 40MG	STAR MEDICAL SUPPLIES LTD	HK-65152	No
1917		SIMCARD-20 TAB 20MG	CONTROLLED MEDICATIONS LTD.	HK-49920	Unknown

1918		SIMOTIN TAB 20MG	EUROPHARM LAB CO LTD	HK-52045	No
1919		SIMPLAQOR TAB 40MG	NOVARTIS PHARMACEUTICALS (HK) LIMITED	HK-58991	No
1920		SIMVA-DENK 20 TAB 20MG	STAR MEDICAL SUPPLIES LTD	HK-60918	No
1921		SIMVACOR TAB 20MG	YUNG SHIN CO LTD	HK-55334	Unknown
1922		SIMVARET 20 TABLETS 20MG	SWEDISH TRADING COMPANY LIMITED	HK-61754	Unknown
1923		SIMVASTATIN GP TABLETS 20MG	CNW (HK) LTD	HK-65582	Unknown
1924		SIMVASTATINA CINFA TAB 40MG	REICH PHARM LIMITED	HK-53172	Unknown
1925		SIMVATADIN TAB 20MG	WILSON TRADING COMPANY LIMITED	HK-61255	Unknown
1926		SIMVATEROL TAB 20MG	HANG LUNG TRADING (H.K.) CO	HK-53767	Unknown
1927		SIMVATIN 20 TAB 20MG	DELTAPHARM LIMITED	HK-53321	Unknown
1928		SIMVELL TAB 10MG	HEALTHCARE PHARMASCIENCE LIMITED	HK-57425	Unknown
1929		SIMVOGET TAB 20MG	CHARIOT PHARMA LIMITED	HK-57274	Unknown
1930		SINTY F.C. TAB 20MG	KAI YUEN PHARMACEUTICAL CO	HK-58252	Unknown
1931		SINVASTATINA FARMOZ TAB 40MG	TRENTON-BOMA LTD	HK-57499	Yes
1932		STAVID TAB 20MG	HOVID LIMITED	HK-55052	Unknown
1933		VAS TAB 40MG	UNICORN LABORATORIES O/B AMERICAN UNICORN LABORATORIES LIMITED	HK-55637	Unknown
1934		VASITIMB TABLETS 10MG/40MG	SINO PACIFIC PHARMA COMPANY LIMITED	HK-67112	Unknown
1935		VATATIN F.C. TAB 20MG 'STANDARD'	KAI YUEN PHARMACEUTICAL CO	HK-57870	Unknown
1936		VIDASTAT TAB 20MG	UNAM CORPORATION LTD	HK-52036	Unknown
1937		VYTORIN TAB 10MG/40MG	ORGANON HONG KONG LIMITED	HK-53017	Unknown
1938		ZOCOR TAB 20MG	ORGANON HONG KONG LIMITED	HK-34336	Unknown
1939		ZOSTATIN F.C. TAB 20MG "S.C."	STAR MEDICAL SUPPLIES LTD	HK-57010	Yes
1940	**CARBAMAZEPINE**	APO-CARBAMAZEPINE TAB 200MG	HIND WING CO LTD	HK-40924	Unknown
1941		CARBAMAZEPINE-TEVA TAB 200MG	TEVA PHARMACEUTICAL HONG KONG O/B TEVA PHARMACEUTICAL HONG KONG LIMITED	HK-53796	Unknown
1942		CARZEPIN TAB 200MG	HOVID LIMITED	HK-35635	Unknown
1943		TAVER TAB 200MG	STAR MEDICAL SUPPLIES LTD	HK-40975	No
1944		TEGRETOL CR 200 TAB 200MG	NOVARTIS PHARMACEUTICALS (HK) LIMITED	HK-44773	Yes
1945		TEGRETOL SYRUP 2%	NOVARTIS PHARMACEUTICALS (HK) LIMITED	HK-35117	Yes
1946		TEGRETOL TAB 200MG	NOVARTIS PHARMACEUTICALS (HK) LIMITED	HK-44772	No
1947	**INDOMETHACIN**	ANATON CAPSULE (NEW FORMULA)	EUROPHARM LAB CO LTD	HK-62894	No
1948		APT-INDOMETHACIN 25 CAP 25MG	SYNCO (H.K.) LIMITED	HK-42175	No
1949		ARTHREXIN CAP 25MG	LUEN CHEONG HONG LTD	HK-33392	Unknown
1950		ATROFIN-A (NEW FORMULA) CAPSULES	EUROPHARM LAB CO LTD	HK-62428	No
1951		BONE ZONE CAPSULES	ADVANCE PHARMACEUTICAL COMPANY LIMITED	HK-63566	Unknown
1952		BUFFORI (NEW FORMULA) CAPSULES	EUROPHARM LAB CO LTD	HK-62433	No
1953		CASSULIE (NEW FORMULA) CAPSULES	EUROPHARM LAB CO LTD	HK-62442	No
1954		CERVICON (NEW FORMULA) CAPSULES	EUROPHARM LAB CO LTD	HK-62430	No
1955		DIFOMIN (NEW FORMULA) CAPSULES	EUROPHARM LAB CO LTD	HK-62443	No
1956		DONAPAIN CAPSULE (NEW FORMULA)	EUROPHARM LAB CO LTD	HK-62896	No
1957		EFORMAT INDOMETHACIN CAPSULES 25MG	WELLDONE PHARMACEUTICALS LIMITED	HK-67280	Unknown
1958		ETROFIN (NEW FORMULA) CAPSULES	EUROPHARM LAB CO LTD	HK-62435	No
1959		FLUCLOX (NEW FORMULA) CAPSULES	EUROPHARM LAB CO LTD	HK-62438	No
1960		GOLDIS (NEW FORMULA) CAPSULES	EUROPHARM LAB CO LTD	HK-62439	No
1961		GOPAINLIN CAPSULE (NEW FORMULA)	EUROPHARM LAB CO LTD	HK-62893	No
1962		INDOCID CAP 25MG	ASPEN ASIA COMPANY LIMITED	HK-01934	Unknown
1963		INDOME CAPSULES 25MG	WELLDONE PHARMACEUTICALS LIMITED	HK-67281	Unknown
1964		INDOMETHACIN CAP "SC" 25MG	STAR MEDICAL SUPPLIES LTD	HK-57190	No
1965		INDOMETHACIN CAP 25MG	MEYER PHARMACEUTICALS LTD	HK-23627	Unknown
1966		INDOMETHACIN CAP 25MG	CHRISTO PHARM LTD	HK-23977	No
1967		INDOMETHACIN CAP 25MG	VICKMANS LABORATORIES LTD	HK-21046	No
1968		INDOMETHACIN CAP 25MG	NATIONAL PHARMACEUTICAL CO LTD	HK-15122	No
1969		INDOMETHACIN CAP 25MG	NICE LABORATORIES LTD	HK-11500	Unknown
1970		INDOMETHACIN CAP 25MG	SYNCO (H.K.) LIMITED	HK-06856	No
1971		INDOMETHACIN CAP 25MG	UNICORN LABORATORIES O/B AMERICAN UNICORN LABORATORIES LIMITED	HK-53989	Unknown
1972		INDOMETHACIN CAP 25MG O/W	MEYER PHARMACEUTICALS LTD	HK-37529	Unknown
1973		INDYLON CAP 25MG	MEDOCHEMIE (HONG KONG) LIMITED	HK-27094	Unknown
1974		INDYLON SUPP 100MG	STAR MEDICAL SUPPLIES LTD	HK-32463	No
1975		LOGIFUL (NEW FORMULA) CAPSULES	EUROPHARM LAB CO LTD	HK-62444	No
1976		MARRINIS (NEW FORMULA) CAPSULES	EUROPHARM LAB CO LTD	HK-62429	No
1977		MELPAIN S CAPSULES	ADVANCE PHARMACEUTICAL COMPANY LIMITED	HK-63567	Unknown
1978		METHACIN CAP 25MG	HOVID LIMITED	HK-35630	Unknown
1979		PAINGOSIC S CAPSULES	ADVANCE PHARMACEUTICAL COMPANY LIMITED	HK-63568	Unknown
1980		PARLOVIA (NEW FORMULA) CAPSULES	EUROPHARM LAB CO LTD	HK-62434	No
1981		PHILDAX (NEW FORMULA) CAPSULES	EUROPHARM LAB CO LTD	HK-62431	No
1982		PRODEXCAM PRO CAPSULES	ADVANCE PHARMACEUTICAL COMPANY LIMITED	HK-63569	Unknown
1983		RAPOSID (NEW FORMULA) CAPSULES	EUROPHARM LAB CO LTD	HK-62446	No
1984		RHEUMAXIN CAP 50MG	VICKMANS LABORATORIES LTD	HK-46461	No
1985		ROLESS CAPSULE (NEW FORMULA)	EUROPHARM LAB CO LTD	HK-62895	No
1986		SANROX (NEW FORMULA) CAPSULES	EUROPHARM LAB CO LTD	HK-62432	No
1987		SAPTOPING CAPSULES	ADVANCE PHARMACEUTICAL COMPANY LIMITED	HK-63570	Unknown
1988		SENTERLIC CAPSULES	ADVANCE PHARMACEUTICAL COMPANY LIMITED	HK-63571	Unknown
1989		SILPATONE CAPSULES	ADVANCE PHARMACEUTICAL COMPANY LIMITED	HK-63573	Unknown
1990		SONIMIN (NEW FORMULA) CAPSULES	EUROPHARM LAB CO LTD	HK-62441	No
1991		STAMFORD (NEW FORMULA) CAPSULES	EUROPHARM LAB CO LTD	HK-62437	No
1992		SUMITON CAPSULE (NEW FORMULA)	EUROPHARM LAB CO LTD	HK-62892	No
1993		TOSPAN INDOMETHACIN CAPSULES 25MG	WELLDONE PHARMACEUTICALS LIMITED	HK-67282	Unknown
1994		TOTALPAIN PRO CAPSULES	ADVANCE PHARMACEUTICAL COMPANY LIMITED	HK-63572	Unknown
1995		TRANKAL (NEW FORMULA) CAPSULES	EUROPHARM LAB CO LTD	HK-62447	No
1996		TRIDEXCIN PRO CAPSULES	ADVANCE PHARMACEUTICAL COMPANY LIMITED	HK-63574	Unknown
1997		TUROSKA-E (NEW FORMULA) CAPSULES	EUROPHARM LAB CO LTD	HK-62436	No
1998		VESONIC (NEW FORMULA) CAPSULES	EUROPHARM LAB CO LTD	HK-62445	No
1999		WATIONAL (NEW FORMULA) CAPSULES	EUROPHARM LAB CO LTD	HK-62448	No
2000		WHARNIS (NEW FORMULA) CAPSULES	EUROPHARM LAB CO LTD	HK-62440	No

**Unknown PEG status:**	1271	63.6%
**Confirmed NOT to have PEG:**	620	31.0%
**Confirmed to have PEG:**	109	5.5%
